# Complete σ* intramolecular aromatic hydroxylation mechanism through O_2_ activation by a Schiff base macrocyclic dicopper(I) complex

**DOI:** 10.3762/bjoc.9.63

**Published:** 2013-03-20

**Authors:** Albert Poater, Miquel Solà

**Affiliations:** 1 Institut de Química Computacional i Catàlisi and Departament de Química, Universitat de Girona, Campus de Montilivi, E-17071 Girona, Spain; 2 Catalan Institute for Water Research (ICRA), H2O Building, Scientific and Technological Park of the University of Girona, Emili Grahit 101, E-17003 Girona, Spain

**Keywords:** aromatic hydroxylation, C–H bond activation, C–H functionalization, copper, DFT calculations, mechanism, Schiff base

## Abstract

In this work we analyze the whole molecular mechanism for intramolecular aromatic hydroxylation through O_2_ activation by a Schiff hexaazamacrocyclic dicopper(I) complex, [Cu^I^_2_(bsH2m)]^2+^. Assisted by DFT calculations, we unravel the reaction pathway for the overall intramolecular aromatic hydroxylation, i.e., from the initial O_2_ reaction with the dicopper(I) species to first form a Cu^I^Cu^II^-superoxo species, the subsequent reaction with the second Cu^I^ center to form a μ-η^2^:η^2^-peroxo-Cu^II^_2_ intermediate, the concerted peroxide O–O bond cleavage and C–O bond formation, followed finally by a proton transfer to an alpha aromatic carbon that immediately yields the product [Cu^II^_2_(bsH2m-O)(μ-OH)]^2+^.

## Introduction

Bearing in mind the key role of dioxygen in biology, in particular toward Cu and Fe metal centers, being involved in the catalytic cycle of proteins, including dinuclear copper-active sites, such as hemocyanin, tyrosinase and catechol oxidases [1–7], either transporting or activating O_2_, its comprehension is still underway. Efforts in biomimetics have been made to understand the interaction of such prototypical metalloenzymes with dinuclear Cu^I^ complexes with molecular O_2_ [8–10], in particular by modifying the nature of the ligands bonded to the metals [11–14]. On the other hand, a hot topic is still to unravel, either experimentally or by calculations, which of the side-on μ-η^2^:η^2^-peroxo and bis(μ-oxo) isomeric Cu_2_O_2_^2+^ cores are present, and in the case that they exist, to study the feasibility of their interconversion [15–19], tuning either the metallic complex or the reaction conditions [20–23]. Moreover, both Cu_2_O_2_^2+^ cores have been proposed to be the active species in the aromatic hydroxylation process. Indeed, this question still remains controversial [24–26].

Among the recent studies in the field of oxidation of Cu systems, tyrosinase model systems that selectively produce aromatic hydroxylation products [27–32] and methane monooxygenase (MMO) models that yield stable aliphatic hydroxylation compounds [33–34] are the subject of interest, and both aliphatic and aromatic hydroxylations have been analyzed theoretically. In particular, there are detailed studies of pMMO complexes [35–36], showing why they are suitable for the conversion of methane to methanol [37]. On the other hand, several theoretical studies have analyzed the inter- and intramolecular hydroxylation of aromatic rings [38–46]. Most of these studies agree that the aromatic hydroxylation takes place through a peroxo group side-on to the Cu_2_O_2_ core.

**Figure 1:**
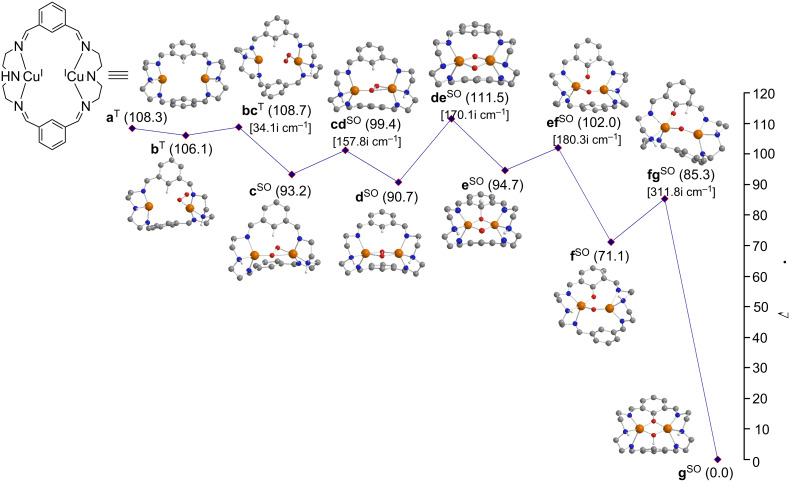
Stationary points located along the reaction path of the aromatic hydroxylation mechanism (some H atoms omitted for clarity). Gibbs energies relative to the product (in kcal·mol^−1^) in solution are given in parentheses. Calculated imaginary frequencies for transition structures are given in brackets. Superindexes SO (open-shell singlet) and T (triplet) refer to the multiplicity of the ground state.

Although from the hexaazamacrocyclic dinuclear Cu^I^ complex [Cu^I^_2_(bsH2m)]^2+^ (**a**) [14] the μ-phenoxo-μ-hydroxo product [Cu^II^_2_(bsH2m-O)(μ-OH)]^2+^ (**g**) was characterized experimentally, it was not possible to trap or detect any intermediate in the path from **a** + O_2_ → **g**. Here, by means of density functional theory (DFT) calculations, we search for the whole reaction pathway (Figure 1). The results are compared with those obtained in a similar previous study in which the hexaazamacrocyclic ligand used (H3m) was more flexible [40]. Crystallographic data on related copper compounds by using the same ligand suggest that complex **a** may present many conformations of rather similar energy [47]; however, the optimized geometries of similar complexes was found to be in perfect agreement with the X-ray structures [40,48–56].

## Computational details

All geometry optimizations, as described in [40], were performed with the Gaussian03 package [57], by using the B3LYP functional [58–60] and the standard 6-31G(d) basis set [61–62]. The geometries obtained at the B3LYP/6-31G(d) level were used to perform single-point energy calculations with a larger basis set, the 6-311G(d,p) basis set [63], and the same functional (B3LYP/6-311G(d,p)//B3LYP/6-31G(d)). Intrinsic reaction pathways were calculated to confirm that the located transition states connected the expected minima. Analytical Hessians were computed to determine the nature of all the stationary points we located, and to calculate zero-point energies (ZPEs) and thermodynamic properties at 298 K.

For open-shell states, the geometry optimizations were performed within the broken-symmetry unrestricted methodology, while for the closed-shell singlet states the restricted formalism was used. Theoretical treatment of biradical singlet species requires multiconfigurational or multireference methods due to strong static electron correlation. Unfortunately, these methods can only be applied to relatively small systems because computationally they are extremely demanding. As an alternative, we have used the unrestricted UB3LYP method in broken symmetry (BS, using GUESS = MIX) [64]. This method improves the modeling of biradical singlet states at the expense of introducing some spin contamination from higher spin states [65–73].

Solvent effects including contributions of non-electrostatic terms have been estimated in single-point calculations on the gas-phase-optimized structures, based on the polarizable continuous solvation model (PCM) with CH_3_CN as a solvent [74–75], i.e., the same solvent used experimentally.

The relative Gibbs energies reported in this work include energies computed using the B3LYP/6-311G(d,p)//B3LYP/6-31G(d) method together with solvent effects obtained at the B3LYP/6-31G(d) level, and zero-point energies, thermal corrections, and entropy effects calculated at 298 K with the B3LYP/6-31G(d) method.

## Results and Discussion

Bearing in mind the easy transformation of **a** to **g**, done at low temperature and atmospheric pressure [14], the coordination of O_2_ gives as a result the formation of a Cu^I^Cu^II^-superoxo species **b** switching the singlet ground state to a triplet, in a barrierless process checked by means of several reaction coordinate linear transits between one or both oxygen atoms and the Cu atoms. The rotation of about 180° of the O_2_ moiety in order to facilitate that the non-bonded oxygen atom points towards the still free Cu atom costs just 2.6 kcal·mol^−1^, evolving to the μ-η^1^:η^2^-peroxo isomer **c** with an energetic stabilization of 12.9 kcal·mol^−1^ with respect to the preceding complex **b**. Furthermore, this step also requires change to a biradical singlet ground state, although the triplet state is only 1 kcal·mol^−1^ higher as a result of the long distance between both Cu atoms that allocate both unpaired electrons [76–77]. To achieve the μ-η^2^:η^2^-peroxo-Cu^II^_2_ isomer **d** only the formation of Cu–O is necessary, the later step having a barrier of 6.2 kcal·mol^−1^ to overcome. Before the formation of this peroxo intermediate **d** the side-on Cu^I^Cu^II^-superoxo isomeric species was not located, probably due the higher rigidity that is imposed by the Schiff bases bsH2m with respect to the similar, previously described system H3m [40].

**Figure 2:**
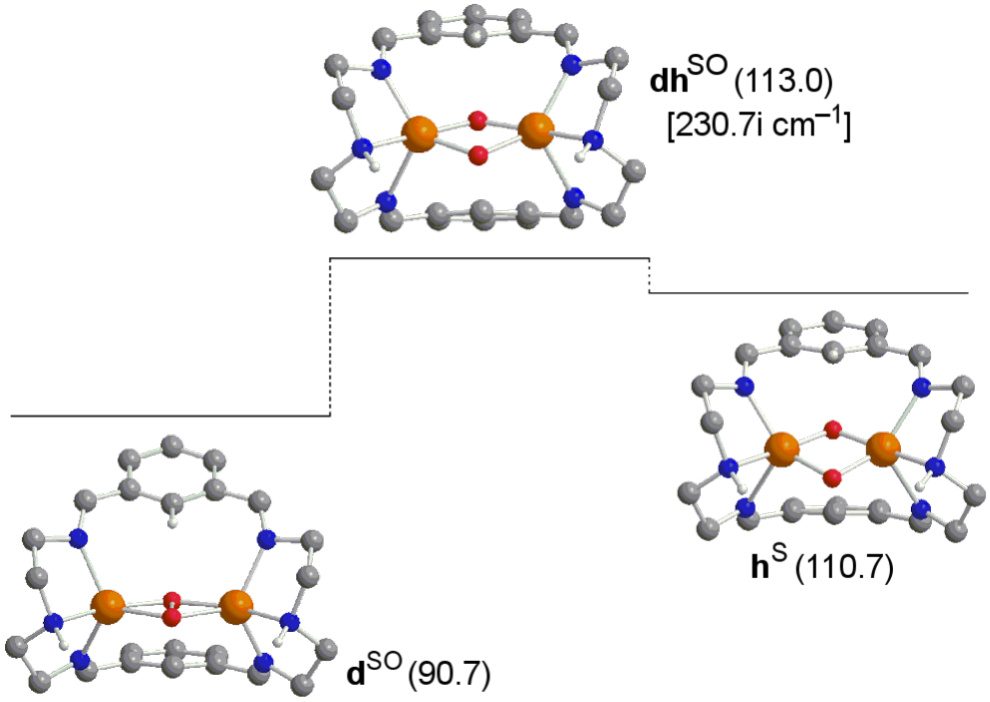
Computed structures of the potential equilibrium between the peroxo and bis-μ-oxo intermediates (some H atoms omitted for clarity). Gibbs energies relative to product **g** (in kcal·mol^−1^) in solution are given in parentheses. Calculated imaginary frequencies for transition structures are given in brackets. Superindexes SO (open-shell singlet) and S (closed-shell singlet) refer to the multiplicity of the ground state.

The two possible routes from **d** to **e** (C–O bond formation) corresponding to the attacks on the two phenyl rings are basically identical, and consequently, only one of them has been analyzed. This step leads to the cleavage of the O–O bond and consists of a direct and concerted attack on the closest carbon atom of the aromatic ring to form species **e** through a barrier of 20.8 kcal·mol^−1^. In an alternative route in Figure 2, the μ-η^2^:η^2^-peroxo-Cu^II^_2_ intermediate **d** might evolve first to the closed-shell singlet bis(μ-oxo)-Cu^III^_2_ isomer (**h**), but this bis(μ-oxo) species is 20.0 kcal·mol^−1^ higher in energy with respect to the peroxo form [40]. Apart from the high instability with respect to the peroxo intermediate, from **d** it is necessary to overcome a barrier of 22.3 kcal·mol^−1^, which rules out the role of **h** in the reaction pathway **a→g**. However, as reported by Cramer [11–12,78], it is necessary to point out that the equilibrium μ-η^2^:η^2^-peroxo/bis(μ-oxo) is artificially displaced towards the peroxo species by hybrid functionals, such as the B3LYP functional, due to unbalanced correlation corrections [11–12]. In spite of that, previous calculations agree in considering that the μ-η^2^:η^2^-peroxo species is the active species in the hydroxylation process studied here [38–40,79–80].

This step from **d** to **e** corresponds to the beginning of the so-called σ* electrophilic mechanism described for *ortho*-hydroxylation towards phenolate [5]. It is worth noting that in the next reaction step, the aromatic H atom in the activated C–H bond of **e** is transferred as a proton to one neighboring aromatic carbon. Amazingly this step requires only 7.3 kcal·mol^−1^. It is necessary to point out that this small barrier comes in part from the breaking of the nearest Cu–O to this proton, which facilitates the electronic arrangement. Finally, overcoming a barrier of 14.2 kcal·mol^−1^ the product is reached when transferring the proton to the other oxygen and rebuilding the broken Cu–O bond.

**Figure 3:**
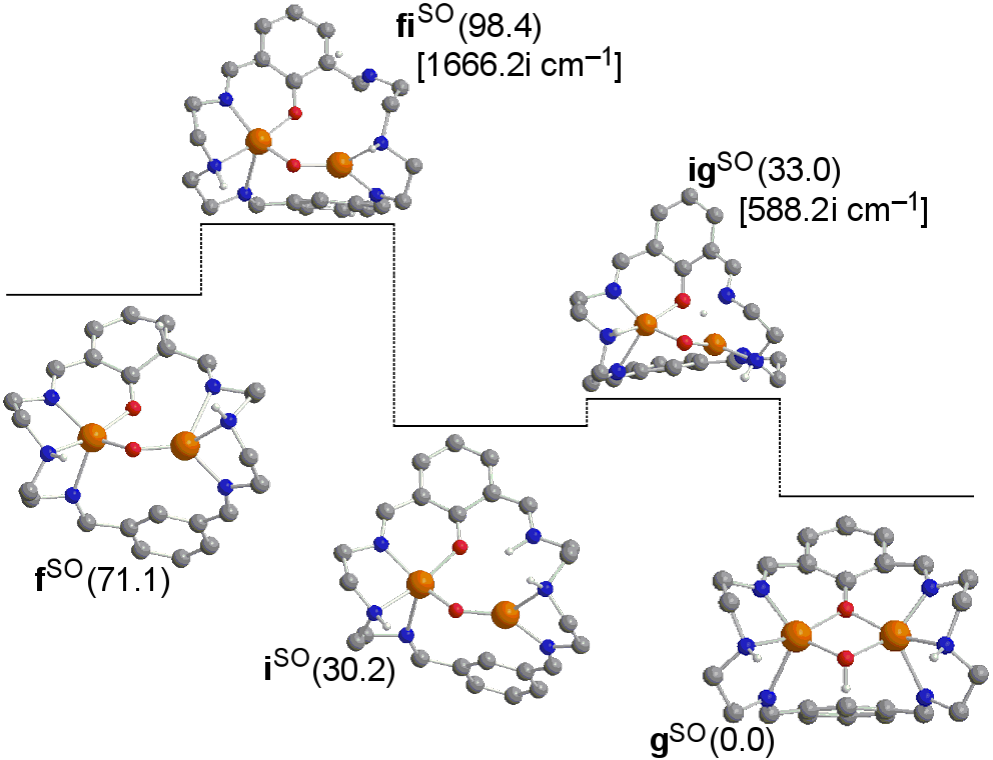
Computed structures for a potential alternative pathway **f→g** of the σ* mechanism (some H atoms omitted for clarity). Gibbs energies relative to product **g** (in kcal·mol^−1^) in solution are given in parentheses. Calculated imaginary frequencies for transition structures are given in brackets. Superindex SO (open-shell singlet) refers to the multiplicity of the ground state.

There are different parallel reactions and competitive intermediates that might be present in the reaction pathway. In Figure 3 the **f→g** step is compared with the migration of the hydrogen to the nearest nitrogen first, and then this nitrogen atom easily throws it to the oxygen bonded to the aromatic ring overcoming a barrier of 2.8 kcal·mol^−1^ in the **i→g** step. However, the upper barrier of 27.3 kcal·mol^−1^ of the step **f→i** in the **f→g** process in Figure 3 must be compared to 14.2 kcal·mol^−1^ of step **f→g** in Figure 1. Thus the migration to the nitrogen atom first is discarded.

**Figure 4:**
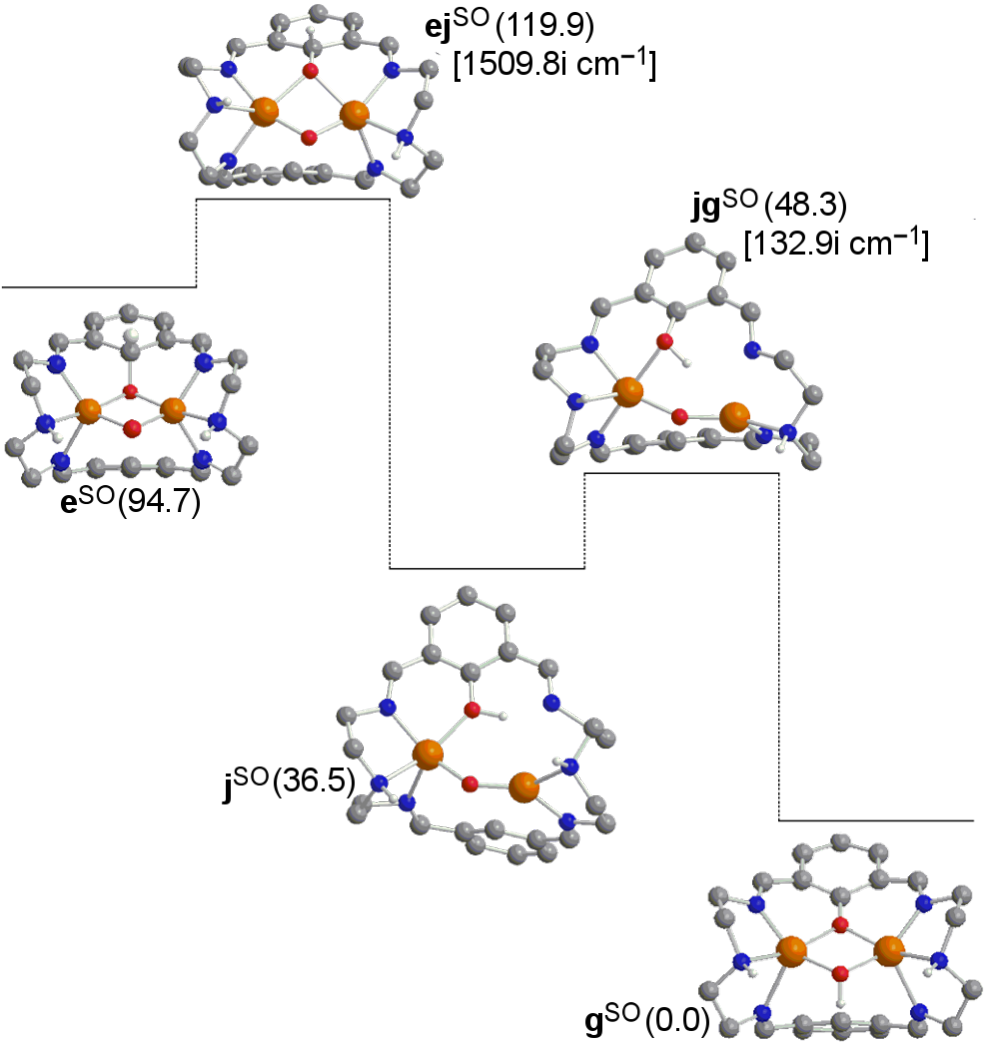
Computed structures for a potential alternative pathway **e→g** of the σ* mechanism (some H atoms omitted for clarity). Gibbs energies relative to product **g** (in kcal·mol^−1^) in solution are given in parentheses. Calculated imaginary frequencies for transition structures are given in brackets. Superindexes SO (open-shell singlet) and T (triplet) refer to the multiplicity of the ground state.

**Figure 5:**
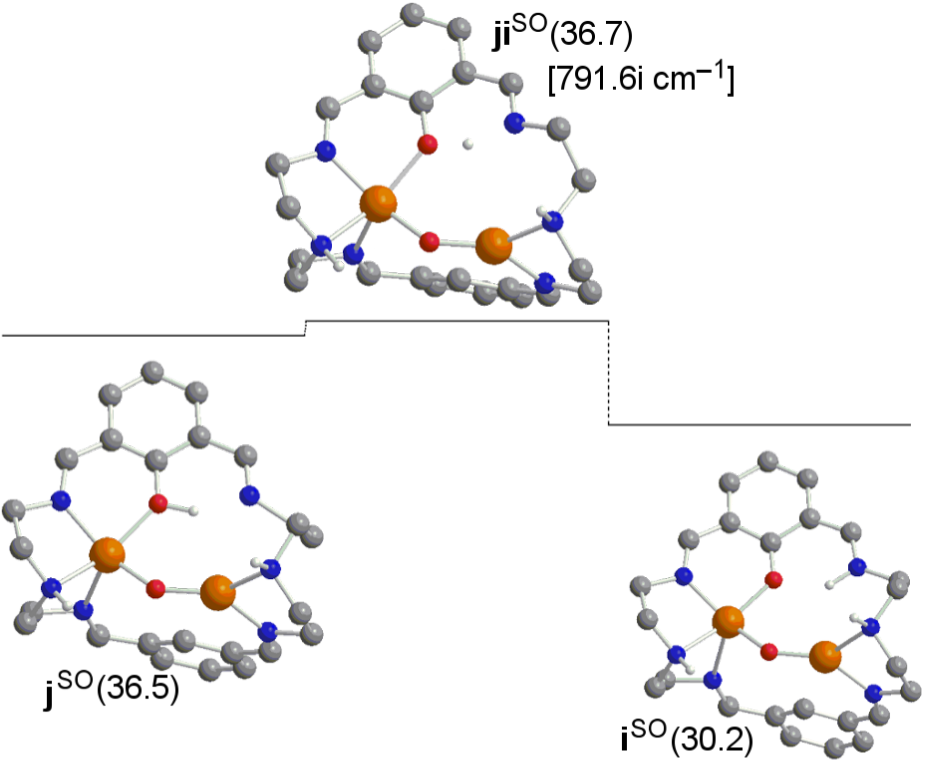
Computed structures for a potential alternative pathway **j→i** of the σ* mechanism (Gibbs energies in kcal·mol^−1^). Superindex SO (open-shell singlet) refers to the multiplicity of the ground state.

In Figure 4, from species **e** the donation of the hydrogen atom to the oxygen bonded to the aromatic carbon would be possible through a barrier of 25.2 kcal·mol^−1^, thus extremely disfavored with respect to 7.3 kcal·mol^−1^ when migrating this hydrogen to one alpha aromatic carbon in the **e→f** step in Figure 1. Then, if this alternative mechanism is taken into account, the subsequent formation of the product from intermediate **j** requires 11.8 kcal·mol^−1^. However, in Figure 5 it is shown that species **j** can evolve towards species **i** overcoming a negligible barrier of 0.2 kcal·mol^−1^. From the Gibbs energies obtained in these alternative pathways, one can conclude that the role played by species **i** and **j** in the whole reaction mechanism is irrelevant.

After the formation of the C–O bond in species **e**, the previously described ligand H3m showed that the other aromatic ring could assist the aromatic proton transfer to the nearer oxygen atom with an upper barrier of only 1.4 kcal·mol^−1^ [40]. However, for bsH2m the distance between the two aromatic rings is always too large for them to help each other. Indeed bsH2m is significantly more rigid, and this factor reduces the degrees of free rotation. However, the upper barrier for bsH2m is only 7.3 kcal·mol^−1^. Thus, the most favored mechanism might change depending on the nature of the chains between the N atoms of the hexaaza ligand. However, the affinity of the peroxo species **d** to interact with either of the aromatic rings is the key factor that decides whether the intramolecular hydroxylation will take place or not [13–26].

**Figure 6:**
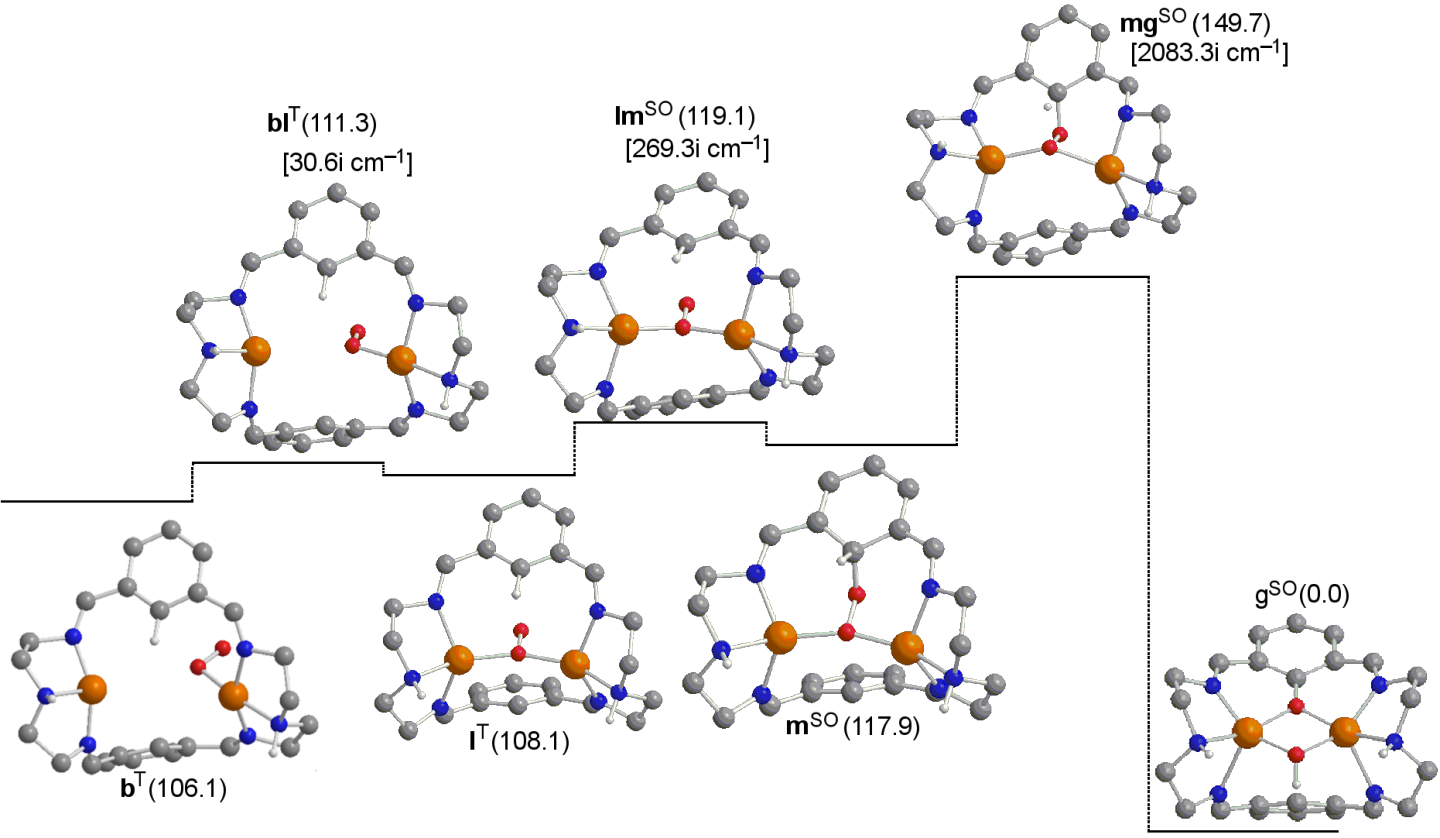
Computed structures for a potential alternative pathway **b→g** of the σ* mechanism (some H atoms omitted for clarity). Gibbs energies relative to product **g** (in kcal·mol^−1^) in solution are given in parentheses. Calculated imaginary frequencies for transition structures are given in brackets. Superindexes SO (open-shell singlet) and T (triplet) refer to the multiplicity of the ground state.

Indeed, intermediates found here are also very different from those found in an aliphatic hydroxylation reaction studied by Holthausen [39]. Thus, in terms of comparison, to broaden the scope of this study, in Figure 6 the study of a different mechanism starting from species **b** was envisaged. Intermediate **l** represents a valid option as a potential reactive intermediate for the direct attack to the aromatic ring by one of the oxygen atoms. The formation of **l** requires that a barrier of only 2.6 kcal·mol^−1^ higher in energy with respect to the formation of species **c** be overcome. And species **l** is 2.0 kcal·mol^−1^ less stable with respect to species **c**. However, although species **l** needs to overcome a barrier of only 11.0 kcal·mol^−1^ to create a C–O bond after the interaction of an oxygen atom with an aromatic ring, the upper barrier from species **b** to the product **g** requires 43.6 kcal·mol^−1^, which is 38.2 kcal·mol^−1^ higher than the upper barrier of the reaction pathway in Figure 1. Thus, the aliphatic hydroxylation scheme is not reproducible here, and thus we can confirm that the aromatic rings play a key role in intramolecular aromatic hydroxylation reactions through O_2_ activation.

**Figure 7:**
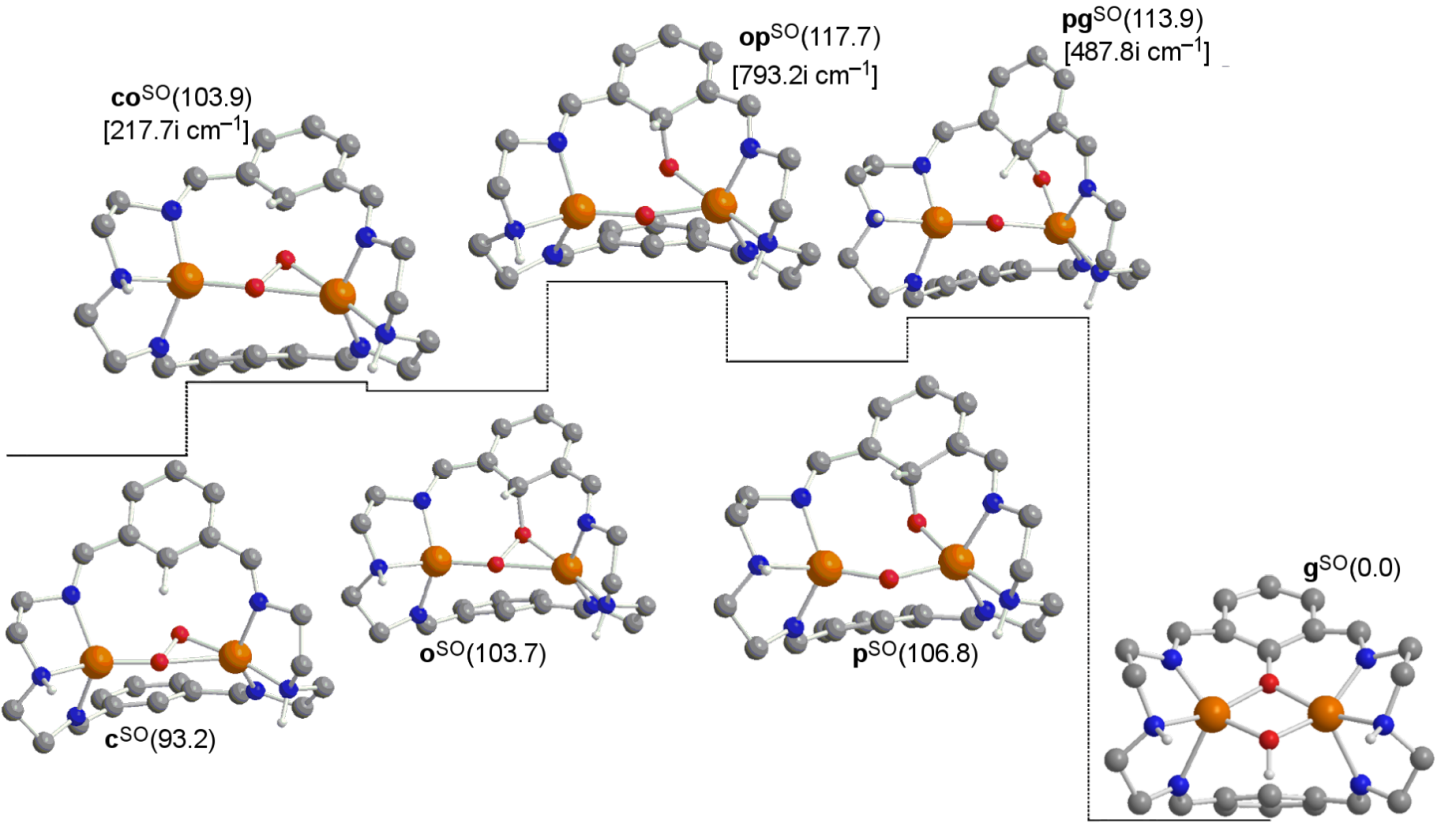
Computed structures for a potential alternative pathway **c→g** of the σ* mechanism (some H atoms omitted for clarity). Gibbs energies relative to product **g** (in kcal·mol^−1^) in solution are given in parentheses. Calculated imaginary frequencies for transition structures are given in brackets. Superindex SO (open-shell singlet) refers to the multiplicity of the ground state.

In Figure 7 the attack on the aromatic ring from species **c** instead of species **d** is displayed. This alternative mechanism reveals an upper energy barrier of 24.5 kcal·mol^−1^ instead of the 18.3 kcal·mol^−1^ described in the mechanism in Figure 1. Thus, the reactivity towards the aromatic rings of the intermediate trans-peroxo (**c**) is worse with respect to the intermediate with a peroxo core (**d**). Finally, comparison of the Gibbs energy profiles of Figure 1 in the present work with those in reference [40], show that energy barriers present in the H3m reaction mechanism [40] are somewhat lower than those found in the more rigid bsH2m ligand, and therefore, the [Cu^I^_2_(H3m)]^2+^ catalyst is expected to be slightly more efficient than the [Cu^I^_2_(bsH2m)]^2+^ one.

Bearing in mind that Mayer Bond Order (MBO) theory gives insight into the strength of the bonds [81–89], MBOs between two atoms A and B were calculated through Equation 1 [90–91], where S is the atomic orbital overlap matrix and P is the density matrix. The sums run over the basis set functions belonging to a given atom A or B.

[1]
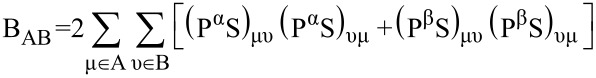


A first glance at Table 1 shows that the in study of the **d→e** step in complexes containing bsH2m and H3m ligands, the MBOs are quite similar. There is a slight difference between the MBOs of the new O–C bond in **e** in the transition state **de**, with values of 0.069 and 0.122 for the [Cu^I^_2_(bsH2m)]^2+^ and [Cu^I^_2_(H3m)]^2+^ systems, respectively. This might help to explain why the barrier for the [Cu^I^_2_(H3m)]^2+^ system is lower than for [Cu^I^_2_(bsH2m)]^2+^ by 8.8 kcal·mol^−1^. However, the differences between the MBOs are small, but this study of the MBOs is not meaningless because it confirms that structurally both systems are similar. On the other hand, to explain the **d→e** step the O···C distance in the peroxo intermediate **d** is key, being 2.601 Å for [Cu^I^_2_(bsH2m)]^2+^ but 2.350 Å for [Cu^I^_2_(H3m)]^2+^, which explains why for the latter system the energy barrier for the **d→e** step is lower. Indeed, for the Cu^I^_2_(bsH2m)]^2+^ system this step displays the upper barrier of the overall reaction pathway **a→g**.

**Table 1:** MBOs for **d→e** step for the [Cu^I^_2_(H3m)]^2+^ and the [Cu^I^_2_(bsH2m)]^2+^ catalysts.

	Intermediate	Cu1–O1	Cu1–O2	Cu2–O1	Cu2–O2	O1–O2	O2–C

[Cu^I^_2_(bsH2m)]^2+^	**d**	0.401	0.382	0.403	0.382	0.889	0.015
	**de**	0.644	0.629	0.684	0.657	0.416	0.069
	**e**	0.558	0.386	0.801	0.462	0.027	0.856
[Cu^I^_2_(H3m)]^2+^	**d**	0.391	0.378	0.391	0.379	0.878	0.017
	**de**	0.640	0.678	0.640	0.678	0.412	0.122
	**e**	0.505	0.822	0.395	0.614	0.040	0.843

## Conclusion

To sum up, the intramolecular hydroxylation of a Schiff base hexaazamacrocyclic dicopper(I) complex (**a**) by means of O_2_ to finally yield the μ-phenoxo-μ-hydroxo product (**g**) occurs thanks to a σ*-mechanism that proceeds through a μ-η^2^:η^2^-peroxo species. Bearing in mind the DFT calculations for the full reaction pathway, it is feasible to explain why it is difficult to characterize experimentally any intermediate, particularly for two reasons: first the lack of high energy barriers, and second the cascade of the energy decay to the product. Furthermore, we provide a detailed analysis of potential alternative reaction pathways to reach product (**g**) [40]; however, these different explored paths between intermediates, in all cases, involve higher energy barriers or are not feasible. Finally, comparison of the reaction mechanisms involving hexaazamacrocyclic bsH2m and H3m ligands indicates that the energy barriers present in the H3m reaction mechanism are somewhat lower than those found in the more rigid bsH2m ligand.

## Supporting Information

File 1 Complete computational methods used and xyz coordinates; ChemDraw and full 3D drawings of all stationary points found.
