# Supplementary material for: Complete σ* intramolecular aromatic hydroxylation mechanism through O2 activation by a Schiff base macrocyclic dicopper(I) complex
Source: Beilstein J Org Chem. 2013 Mar 20;9:585–93. doi: 10.3762/bjoc.9.63 (PMC3628990; doi:10.3762/bjoc.9.63)
Supplement: File 1 — Complete computational methods used and xyz coordinates; ChemDraw and full 3D drawings of all stationary points found. [file Beilstein_J_Org_Chem-09-585-s001.pdf]

# Supporting Information

# for

# Complete σ* intramolecular aromatic hydroxylation mechanism through $O_2$ activation by a Schiff base macrocyclic dicopper(I) complex

Albert Poater[1,2*] and Miquel Solà[1]

Address: [1]Institut de Química Computacional i Catàlisi and Departament de Química, Universitat de Girona, Campus de Montilivi, E-17071 Girona, Spain and [2]Catalan Institute for Water Research (ICRA), $H_2O$ Building, Scientific and Technological Park of the University of Girona, Emili Grahit 101, E-17003 Girona, Spain.

Email: Albert Poater - albert.poater@udg.edu

*Corresponding author

**Complete computational methods used and xyz coordinates; ChemDraw and full 3D drawings of all stationary points found**

## COMPUTATIONAL DETAILS

All geometry optimizations were performed at the B3LYP level,[1-3] using the standard 6-31G(d) basis set[4] with the Gaussian03 package.[5] We did single-point energy calculations with the larger basis set 6-311G(d,p). The geometry optimizations were performed without symmetry constraints, and the nature of extrema was checked by analytical frequency calculations. Furthermore, all extrema were confirmed by calculation of the intrinsic reaction paths. The energies discussed throughout the text contain ZPE corrections.

Solvent effects including contributions of nonelectrostatic terms were estimated in single-point calculations on the gas-phase-optimized structures, based on the polarizable continuous solvation model (PCM) with $CH_3CN$ as a solvent. The solvent effect was introduced by the conductor polarizable calculation model (CPCM).[6] The cavity is created by a series of overlapping spheres.

Based on the optimized geometries of complexes **a-g** spin-restricted and spin-unrestricted DFT calculations were performed. The singlet, the biradical singlet, the triplet and the quintuplet multiplicity states have been thoroughly studied. The quintuplet is farther in energy, as is the closed-shell singlet. Then the energy difference between the biradical singlet and the triplet state was studied in detail, because differences no larger than 5 kcal·mol$^{-1}$ were found for all the extrema of the whole mechanism.

**Table S1:** xyz coordinate data sets, Lewis and 3D representation for DFT (B3LYP/6-31G(d) method using Gaussian03) optimized complexes.

## a

| | | | |
|---|---|---|---|
| H | -5.611548 | -1.510197 | -0.049298 |
| H | -4.366760 | -1.704064 | -2.181893 |
| C | -4.529443 | -1.590497 | -0.039701 |
| H | -4.405213 | -1.712050 | 2.104660 |
| C | -3.825823 | -1.680602 | -1.239685 |
| C | -3.847509 | -1.685107 | 1.172374 |
| C | -2.421620 | -1.769838 | -1.237634 |
| C | -2.443504 | -1.774702 | 1.195188 |
| H | -2.234655 | -2.797572 | -3.145158 |
| C | -1.733077 | -1.775115 | -0.014971 |
| H | -2.294304 | -2.806480 | 3.103604 |
| C | -1.711465 | -2.103474 | -2.477698 |
| C | -1.756311 | -2.113871 | 2.446449 |
| H | -0.658409 | -1.924688 | -0.005561 |
| N | -0.518648 | -1.722884 | -2.786654 |
| N | -0.566760 | -1.740413 | 2.776107 |
| H | -0.684974 | -2.973199 | 4.523256 |
| H | -0.599857 | -2.960837 | -4.532385 |
| C | 0.065743 | -2.462439 | 3.908666 |
| C | 0.136471 | -2.437670 | -3.910833 |
| H | 0.713813 | -3.232243 | 3.469782 |
| H | 0.791107 | -3.196854 | -3.463324 |
| C | 0.324052 | 2.849606 | -2.514837 |
| H | 0.392824 | 3.678298 | -3.227016 |
| C | 0.319372 | 2.823055 | 2.536959 |
| H | 0.391684 | 3.646170 | 3.255270 |
| C | 0.171384 | 2.634810 | 0.009663 |
| C | -0.180746 | 3.254141 | -1.200728 |
| C | -0.183230 | 3.240724 | 1.226001 |
| H | -1.197912 | 4.926266 | -2.117994 |
| C | -0.945975 | 4.438232 | -1.180268 |
| C | -0.948237 | 4.425206 | 1.216765 |
| H | -1.201866 | 4.903128 | 2.159231 |
| Cu | 0.414113 | -0.155108 | 2.455038 |
| Cu | 0.442885 | -0.128936 | -2.450679 |
| C | -1.364676 | 4.999531 | 0.020961 |
| H | -1.962041 | 5.905204 | 0.025349 |
| N | 0.692154 | 1.681842 | -2.916299 |
| N | 0.681468 | 1.650683 | 2.930603 |
| H | 0.288216 | -0.767702 | -5.256343 |
| H | 0.228043 | -0.793148 | 5.253727 |
| C | 0.963081 | -1.474833 | -4.764123 |
| C | 0.897526 | -1.510355 | 4.768819 |
| N | 1.850729 | -0.764694 | 3.916875 |
| N | 1.897472 | -0.714429 | -3.904154 |
| H | 1.480412 | -2.026895 | -5.558803 |
| H | 1.399117 | -2.068761 | 5.569160 |
| H | 2.658861 | -1.352522 | 3.720372 |
| H | 2.713762 | -1.289061 | -3.702444 |
| H | 0.383374 | 1.386174 | -4.973931 |
| H | 0.365848 | 1.357603 | 4.987750 |
| C | 1.220386 | 1.629676 | -4.308061 |
| C | 2.307190 | 0.529997 | 4.461505 |
| C | 1.207451 | 1.588438 | 4.323181 |
| C | 2.336548 | 0.588060 | -4.445021 |
| H | 3.183118 | 0.836419 | 3.881490 |
| H | 3.205822 | 0.906683 | -3.861612 |
| H | 2.608351 | 0.465643 | 5.516290 |
| H | 2.642083 | 0.529853 | -5.498874 |
| H | 1.602690 | 2.607783 | -4.617925 |
| H | 1.604496 | 2.560227 | 4.634263 |
| H | 0.797841 | 1.747655 | 0.005116 |

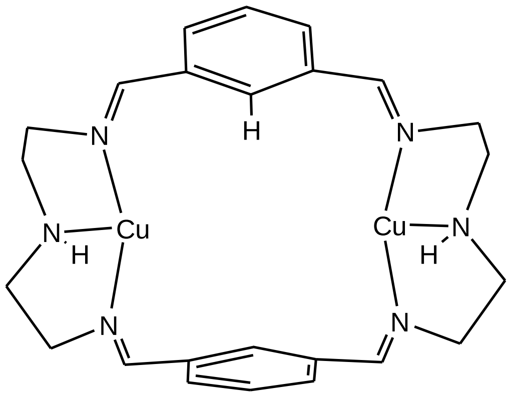


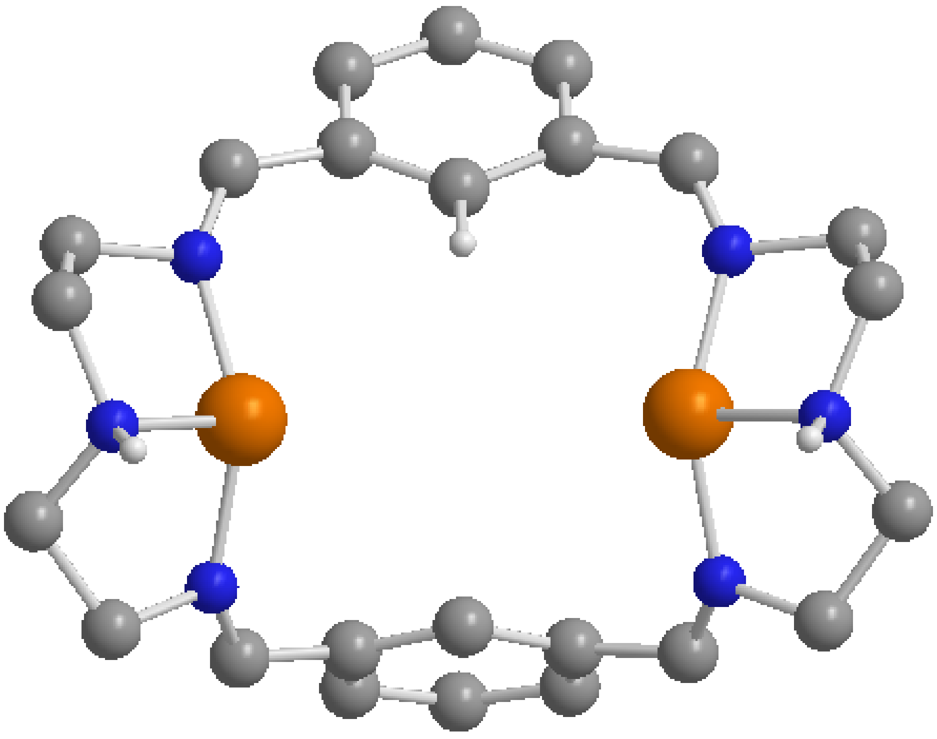

**b**

| | | | |
|---|---|---|---|
| H | -5.408240 | -2.733676 | 0.716141 |
| H | -4.779206 | -2.314189 | -1.644638 |
| C | -4.374941 | -2.527243 | 0.457660 |
| H | -3.648452 | -2.856010 | 2.453446 |
| C | -4.020949 | -2.276720 | -0.867113 |
| C | -3.385949 | -2.578524 | 1.435994 |
| C | -2.684542 | -2.006209 | -1.210893 |
| C | -2.044608 | -2.284936 | 1.116248 |
| H | -3.064564 | -2.419750 | -3.302791 |
| C | -1.706001 | -1.973648 | -0.203411 |
| H | -1.151607 | -3.428287 | 2.727997 |
| C | -2.330084 | -1.964282 | -2.628654 |
| C | -1.011186 | -2.516862 | 2.135812 |
| H | -0.668160 | -1.801230 | -0.467661 |
| N | -1.220877 | -1.534666 | -3.119657 |
| N | 0.027481 | -1.789168 | 2.370787 |
| H | 0.663761 | -3.311135 | 3.735360 |
| H | -1.785144 | -1.754865 | -5.174494 |
| C | 1.002304 | -2.344325 | 3.345791 |
| C | -0.915631 | -1.893570 | -4.521752 |
| H | 1.942322 | -2.513706 | 2.806395 |
| H | -0.647436 | -2.957730 | -4.546459 |
| C | 1.172344 | 2.756362 | -2.371725 |
| H | 1.646193 | 3.510354 | -3.010980 |
| C | -0.337627 | 2.998338 | 2.462900 |
| H | -0.595945 | 3.770821 | 3.193821 |
| C | 0.322369 | 2.743584 | 0.038310 |
| C | 0.500617 | 3.349585 | -1.213647 |
| C | -0.260582 | 3.475260 | 1.084219 |
| H | 0.248959 | 5.163689 | -2.364330 |
| C | 0.079968 | 4.683920 | -1.404029 |
| C | -0.707337 | 4.791863 | 0.855931 |
| H | -1.156987 | 5.351413 | 1.671823 |
| Cu | 0.326299 | 0.079368 | 2.277434 |
| Cu | 0.078129 | -0.114968 | -2.499175 |
| C | -0.555285 | 5.390356 | -0.389466 |
| H | -0.894936 | 6.406763 | -0.557675 |
| N | 1.256038 | 1.528063 | -2.756626 |
| N | -0.088631 | 1.812163 | 2.896288 |
| H | -0.060021 | -0.040324 | -5.256882 |
| H | 0.269760 | -1.190201 | 5.005785 |
| C | 0.266043 | -1.058251 | -5.020892 |
| C | 1.223464 | -1.357833 | 4.493732 |
| N | 1.703017 | -0.065733 | 3.958782 |
| N | 1.270221 | -0.984931 | -3.928260 |
| H | 0.685925 | -1.491122 | -5.936699 |
| H | 1.913536 | -1.786749 | 5.231198 |
| H | 2.714003 | -0.107267 | 3.847274 |
| H | 1.587661 | -1.931004 | -3.714256 |
| H | 1.411488 | 1.655796 | -4.855003 |
| H | -0.821315 | 0.913612 | 4.653430 |
| C | 2.035494 | 1.340166 | -4.009541 |
| C | 1.348738 | 1.121280 | 4.769127 |
| C | -0.044957 | 1.632850 | 4.366125 |
| C | 2.444969 | -0.119082 | -4.167047 |
| H | 2.088121 | 1.900161 | 4.560721 |
| H | 3.194526 | -0.380426 | -3.413643 |
| H | 1.371929 | 0.916864 | 5.847658 |
| H | 2.885962 | -0.284672 | -5.157907 |
| H | 2.929679 | 1.971442 | -4.012457 |
| H | -0.260671 | 2.570368 | 4.890884 |
| H | 0.669524 | 1.733273 | 0.234935 |
| O | -1.218014 | 0.762439 | -1.421180 |
| O | -1.954511 | 1.352352 | -2.312224 |

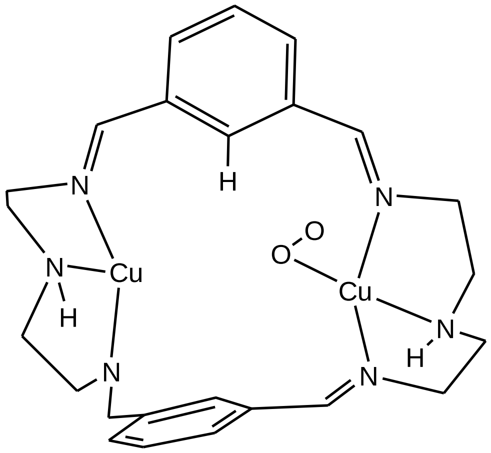


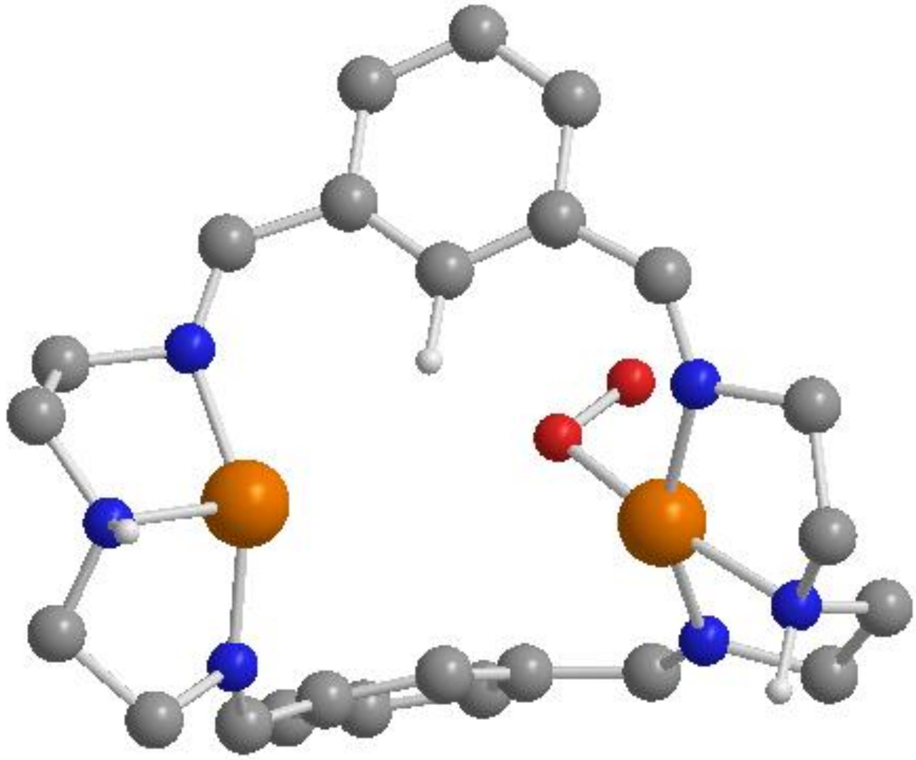

## TS-bc

| | | | |
|---|---|---|---|
| H | -5.174581 | -3.283258 | 0.807699 |
| H | -4.756465 | -2.630255 | -1.547011 |
| C | -4.195459 | -2.924281 | 0.508403 |
| H | -3.305000 | -3.279379 | 2.431034 |
| C | -3.961364 | -2.543634 | -0.811413 |
| C | -3.147398 | -2.905028 | 1.423017 |
| C | -2.696680 | -2.074909 | -1.207872 |
| C | -1.877946 | -2.413730 | 1.052872 |
| H | -3.187739 | -2.389643 | -3.290519 |
| C | -1.666552 | -1.975821 | -0.257467 |
| H | -0.809385 | -3.504036 | 2.585029 |
| C | -2.455115 | -1.899450 | -2.639568 |
| C | -0.785328 | -2.562762 | 2.024607 |
| H | -0.681612 | -1.638509 | -0.565601 |
| N | -1.434970 | -1.331845 | -3.178534 |
| N | 0.167930 | -1.732074 | 2.277615 |
| H | 0.976815 | -3.221956 | 3.585252 |
| H | -2.039569 | -1.183611 | -5.223820 |
| C | 1.187693 | -2.198794 | 3.254127 |
| C | -1.207464 | -1.568915 | -4.623667 |
| H | 2.157423 | -2.207203 | 2.741973 |
| H | -1.140178 | -2.649202 | -4.797963 |
| C | 1.380492 | 2.662688 | -2.299121 |
| H | 1.992960 | 3.349299 | -2.895353 |
| C | -0.447536 | 3.047615 | 2.408823 |
| H | -0.762185 | 3.815930 | 3.121755 |
| C | 0.386688 | 2.750767 | 0.049918 |
| C | 0.714214 | 3.333902 | -1.181251 |
| C | -0.208144 | 3.531133 | 1.051403 |
| H | 0.743806 | 5.168197 | -2.328084 |
| C | 0.452883 | 4.705445 | -1.388814 |
| C | -0.507993 | 4.884696 | 0.800560 |
| H | -0.973133 | 5.481981 | 1.580245 |
| Cu | 0.282818 | 0.155746 | 2.223609 |
| Cu | -0.082251 | -0.038042 | -2.514153 |
| C | -0.187109 | 5.469751 | -0.419160 |
| H | -0.406556 | 6.516217 | -0.602295 |
| N | 1.321160 | 1.435966 | -2.696570 |
| N | -0.266942 | 1.849285 | 2.841279 |
| H | -0.060581 | 0.178075 | -5.210132 |
| H | 0.240241 | -1.218427 | 4.918142 |
| C | 0.104635 | -0.891480 | -5.043225 |
| C | 1.231761 | -1.248075 | 4.453159 |
| N | 1.576189 | 0.112739 | 3.993077 |
| N | 1.083722 | -1.041751 | -3.938733 |
| H | 0.471338 | -1.313643 | -5.986647 |
| H | 1.932605 | -1.625638 | 5.208395 |
| H | 2.588855 | 0.195217 | 3.933313 |
| H | 1.258073 | -2.034320 | -3.782175 |
| H | 1.639415 | 1.585306 | -4.775016 |
| H | -1.087830 | 0.850641 | 4.502043 |
| C | 2.151728 | 1.162899 | -3.901622 |
| C | 1.043134 | 1.222399 | 4.816983 |
| C | -0.348740 | 1.635547 | 4.303240 |
| C | 2.368861 | -0.333871 | -4.088614 |
| H | 1.724868 | 2.071579 | 4.713128 |
| H | 3.035833 | -0.715649 | -3.309201 |
| H | 0.988072 | 0.966222 | 5.882996 |
| H | 2.841373 | -0.522460 | -5.061457 |
| H | 3.123029 | 1.661430 | -3.824779 |
| H | -0.684071 | 2.539343 | 4.824669 |
| H | 0.619878 | 1.711378 | 0.266893 |
| O | -1.273021 | 1.176492 | -1.625314 |
| O | -2.493149 | 0.862982 | -1.389266 |

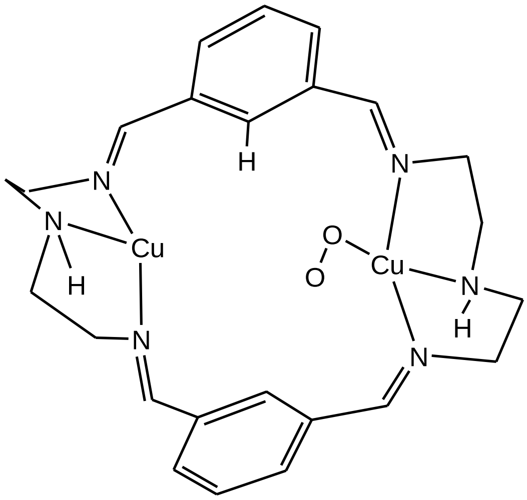


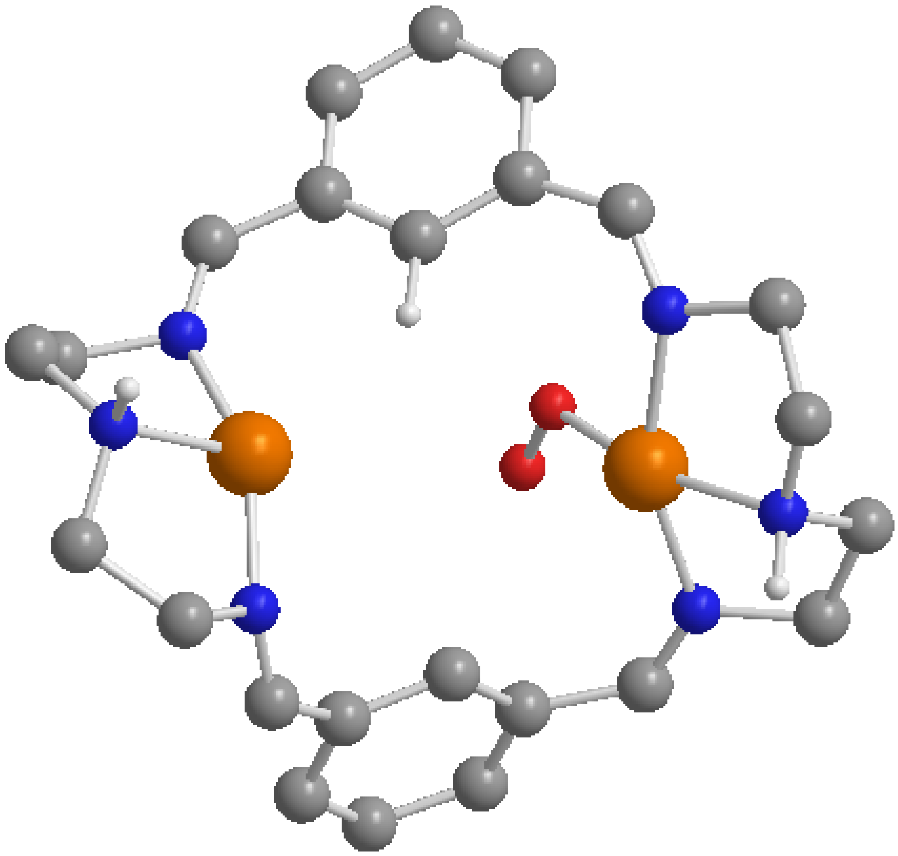

**c**

| | | | |
|---|---|---|---|
| H | -5.617084 | -2.071054 | 0.583983 |
| H | -4.764573 | -1.755389 | -1.720100 |
| C | -4.548191 | -2.031499 | 0.402548 |
| H | -4.029959 | -2.435103 | 2.449930 |
| C | -4.064857 | -1.842969 | -0.892858 |
| C | -3.653646 | -2.224842 | 1.452109 |
| C | -2.681113 | -1.789876 | -1.139244 |
| C | -2.264059 | -2.159853 | 1.222731 |
| H | -2.906238 | -2.160251 | -3.261502 |
| C | -1.783959 | -1.933491 | -0.071168 |
| H | -1.671412 | -3.260583 | 3.005478 |
| C | -2.199267 | -1.771444 | -2.518018 |
| C | -1.330841 | -2.493002 | 2.300091 |
| H | -0.718962 | -1.953715 | -0.251935 |
| N | -1.015087 | -1.442491 | -2.910568 |
| N | -0.148722 | -2.000996 | 2.454577 |
| H | 0.196580 | -3.409345 | 4.026945 |
| H | -1.484154 | -1.826565 | -4.969336 |
| C | 0.716851 | -2.616669 | 3.476391 |
| C | -0.638461 | -1.874380 | -4.272365 |
| H | 1.563654 | -3.079298 | 2.952850 |
| H | -0.327179 | -2.925844 | -4.208244 |
| C | 0.919860 | 2.924098 | -2.582446 |
| H | 1.201217 | 3.682774 | -3.323684 |
| C | -0.215880 | 2.872692 | 2.292261 |
| H | -0.495251 | 3.593962 | 3.069162 |
| C | 0.326706 | 2.838695 | -0.157066 |
| C | 0.200063 | 3.433154 | -1.420339 |
| C | -0.357577 | 3.386253 | 0.936425 |
| H | -0.649801 | 5.070809 | -2.550628 |
| C | -0.583501 | 4.593024 | -1.576410 |
| C | -1.153045 | 4.536622 | 0.758005 |
| H | -1.665860 | 4.971873 | 1.611971 |
| Cu | 0.635782 | -0.220286 | 1.927526 |
| Cu | 0.407541 | -0.124502 | -2.183552 |
| C | -1.275989 | 5.131480 | -0.494501 |
| H | -1.884447 | 6.020556 | -0.621300 |
| N | 1.250040 | 1.696765 | -2.805925 |
| N | 0.207953 | 1.714270 | 2.673585 |
| H | 0.165838 | -0.033051 | -5.058216 |
| H | 0.366212 | -1.161008 | 5.021122 |
| C | 0.522299 | -1.030080 | -4.785716 |
| C | 1.209811 | -1.537738 | 4.435129 |
| N | 1.764524 | -0.402004 | 3.648874 |
| N | 1.517528 | -0.878449 | -3.690298 |
| H | 0.970752 | -1.480802 | -5.678979 |
| H | 1.950407 | -1.939202 | 5.136003 |
| H | 2.731729 | -0.623504 | 3.408648 |
| H | 1.905304 | -1.798513 | -3.473765 |
| H | 1.522882 | 1.680866 | -4.908696 |
| H | -0.426205 | 0.921584 | 4.519757 |
| C | 2.102694 | 1.483527 | -3.998429 |
| C | 1.736199 | 0.894718 | 4.380712 |
| C | 0.382547 | 1.555067 | 4.131317 |
| C | 2.632489 | 0.053771 | -3.991008 |
| H | 2.534043 | 1.527772 | 3.982134 |
| H | 3.375123 | -0.067572 | -3.196606 |
| H | 1.916230 | 0.751958 | 5.452641 |
| H | 3.111950 | -0.185714 | -4.947879 |
| H | 2.948902 | 2.178234 | -3.998949 |
| H | 0.329627 | 2.514065 | 4.658978 |
| H | 1.009948 | 2.015337 | -0.009618 |
| O | -0.407973 | 0.379903 | -0.606340 |
| O | 0.705814 | -0.215435 | 0.076383 |

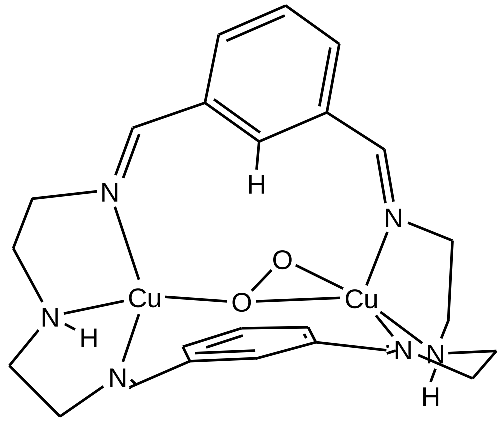


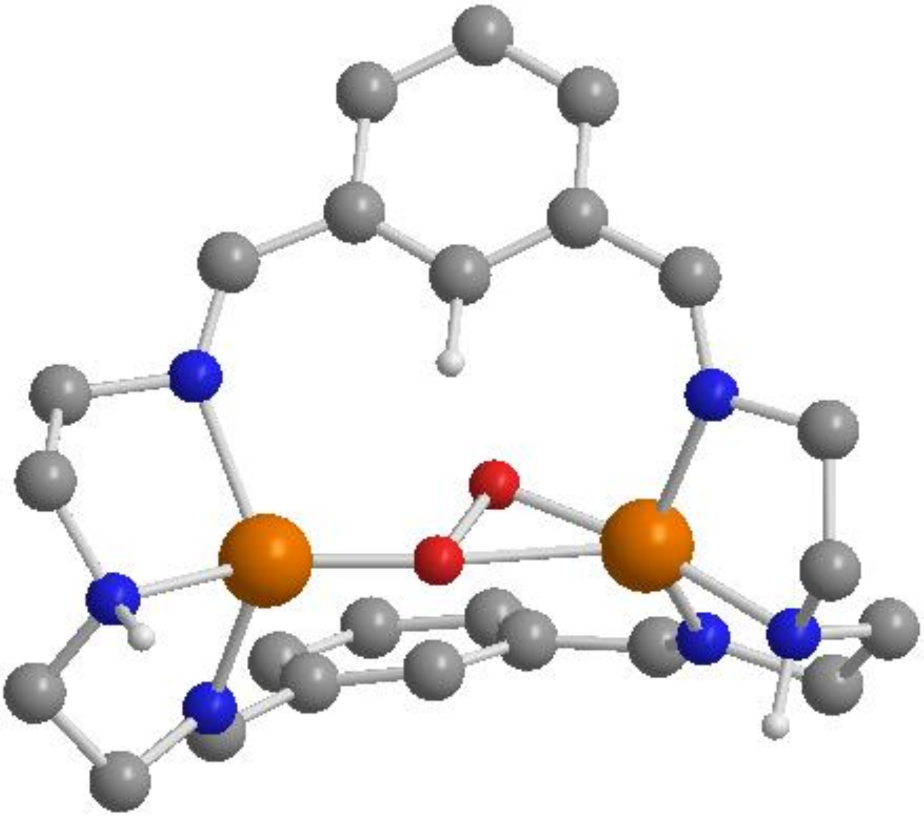

# TS-cd

| | | | |
|---|---|---|---|
| H | 0.401721 | -3.414788 | 4.609427 |
| H | -1.848768 | -3.150390 | 3.612312 |
| C | 0.302433 | -3.155398 | 3.560563 |
| H | 2.421983 | -3.210585 | 3.191653 |
| C | -0.964701 | -2.997416 | 2.998754 |
| C | 1.439800 | -3.030463 | 2.762773 |
| C | -1.102858 | -2.656232 | 1.640870 |
| C | 1.317476 | -2.688826 | 1.403714 |
| H | -3.178831 | -3.278962 | 1.478039 |
| C | 0.045982 | -2.501291 | 0.850606 |
| H | 3.173295 | -3.538691 | 0.607258 |
| C | -2.428373 | -2.618136 | 1.026979 |
| C | 2.467856 | -2.706767 | 0.489542 |
| H | -0.050593 | -2.336414 | -0.212733 |
| N | -2.758214 | -1.936891 | -0.019831 |
| N | 2.623790 | -1.879618 | -0.483887 |
| H | 4.230723 | -3.087057 | -1.207950 |
| H | -4.729602 | -2.778585 | 0.027308 |
| C | 3.596371 | -2.250944 | -1.528256 |
| C | -4.039891 | -2.281416 | -0.665649 |
| H | 3.006943 | -2.599398 | -2.387004 |
| H | -3.815612 | -2.994515 | -1.470751 |
| C | -2.403205 | 2.758782 | 0.361153 |
| H | -3.138516 | 3.552926 | 0.540509 |
| C | 2.576327 | 2.480552 | 0.932520 |
| H | 3.353159 | 3.148421 | 1.321609 |
| O | -0.398877 | -0.010144 | -0.030725 |
| C | 0.089108 | 2.515362 | 0.662494 |
| C | -1.173452 | 2.893640 | 1.144791 |
| C | 1.230724 | 2.742822 | 1.443571 |
| H | -2.249112 | 3.848985 | 2.760889 |
| C | -1.277211 | 3.519989 | 2.402070 |
| O | 0.160089 | -0.049745 | -1.335380 |
| C | 1.104040 | 3.357700 | 2.705057 |
| H | 1.992973 | 3.558280 | 3.297464 |
| Cu | 1.937270 | 0.026190 | -0.809980 |
| Cu | -2.099947 | -0.127005 | -0.743930 |
| C | -0.146936 | 3.726808 | 3.190473 |
| H | -0.238325 | 4.203308 | 4.160842 |
| N | -2.712042 | 1.833991 | -0.483611 |
| N | 2.914321 | 1.590318 | 0.063778 |
| H | -5.054076 | -0.390500 | -0.443486 |
| H | 5.142911 | -0.818433 | -1.124529 |
| C | -4.686784 | -1.029867 | -1.251010 |
| C | 4.463308 | -1.066860 | -1.944098 |
| N | 3.624042 | 0.121862 | -2.233296 |
| N | -3.652827 | -0.269737 | -2.005487 |
| H | -5.539434 | -1.288356 | -1.889384 |
| H | 5.086154 | -1.344045 | -2.803194 |
| H | 3.358976 | 0.109817 | -3.216742 |
| H | -3.411762 | -0.799785 | -2.845258 |
| H | -4.826497 | 1.795494 | -0.498245 |
| H | 4.886941 | 0.881283 | 0.094365 |
| C | -4.003780 | 2.021730 | -1.187928 |
| C | 4.313071 | 1.399498 | -1.938165 |
| C | 4.302975 | 1.649332 | -0.427155 |
| C | -4.052939 | 1.105629 | -2.406826 |
| H | 3.767060 | 2.201095 | -2.445142 |
| H | -3.332857 | 1.443780 | -3.157863 |
| H | 5.346927 | 1.409178 | -2.308487 |
| H | -5.051369 | 1.117051 | -2.859701 |
| H | -4.127573 | 3.060354 | -1.511122 |
| H | 4.766788 | 2.617731 | -0.209241 |
| H | 0.194742 | 2.136718 | -0.343351 |

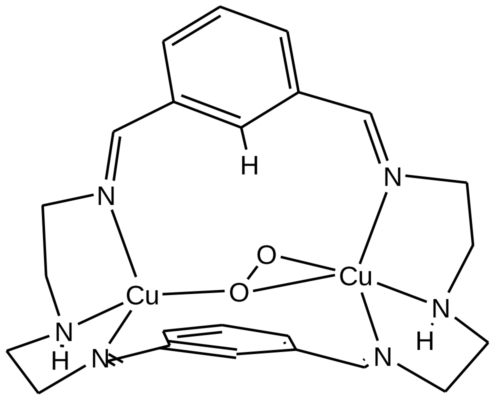


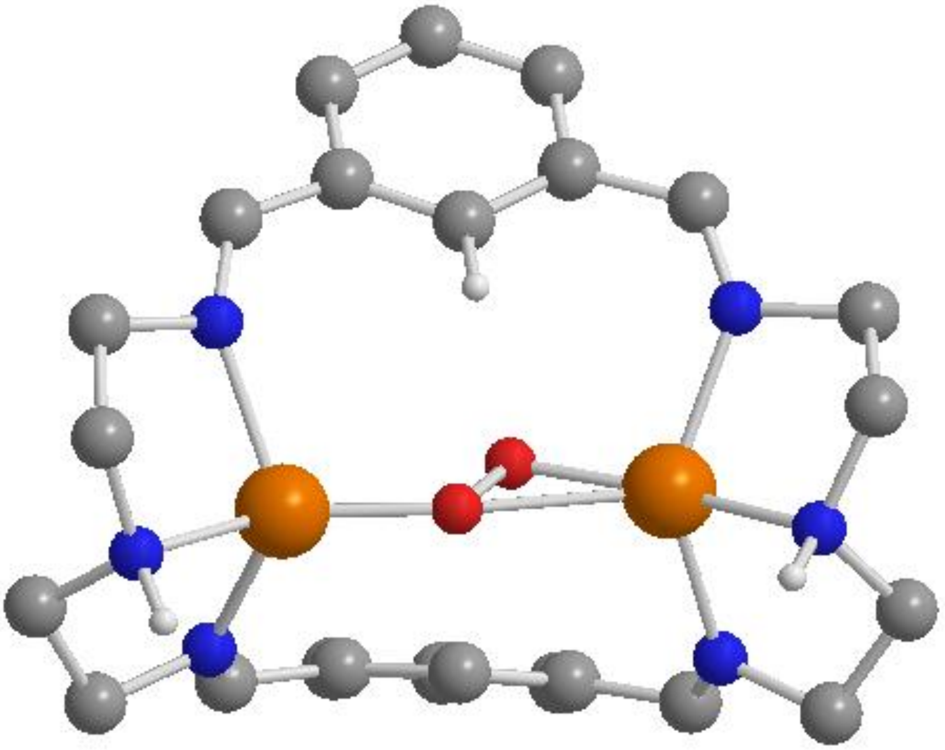

## d

| | | | |
|---|---|---|---|
| H | -5.505283 | -0.939935 | -0.246449 |
| H | -4.153801 | -1.339245 | -2.282121 |
| C | -4.450659 | -1.178476 | -0.155359 |
| H | -4.487411 | -1.179788 | 1.997565 |
| C | -3.686061 | -1.397007 | -1.302801 |
| C | -3.874053 | -1.306966 | 1.109414 |
| C | -2.318367 | -1.703629 | -1.190497 |
| C | -2.507342 | -1.614390 | 1.233478 |
| H | -1.985082 | -2.636843 | -3.147540 |
| C | -1.742819 | -1.819740 | 0.079546 |
| H | -2.506551 | -2.394361 | 3.280532 |
| C | -1.495705 | -2.065131 | -2.349195 |
| C | -1.882678 | -1.894214 | 2.529347 |
| H | -0.720277 | -2.153243 | 0.171290 |
| N | -0.232258 | -1.826195 | -2.421254 |
| N | -0.639390 | -1.673106 | 2.785189 |
| H | -0.847028 | -2.657594 | 4.677363 |
| H | -0.110268 | -2.968717 | -4.229974 |
| C | -0.070811 | -2.324305 | 3.976936 |
| C | 0.536147 | -2.536899 | -3.455350 |
| H | 0.458795 | -3.220816 | 3.626571 |
| H | 1.045976 | -3.370692 | -2.953552 |
| C | 0.451014 | 2.683228 | -2.318473 |
| H | 0.456759 | 3.422312 | -3.128702 |
| C | 0.215289 | 2.778882 | 2.619598 |
| H | 0.154349 | 3.550498 | 3.396854 |
| O | 0.377603 | -0.098668 | 0.199872 |
| C | 0.232308 | 2.670181 | 0.147535 |
| C | -0.251154 | 3.082483 | -1.098861 |
| C | -0.367336 | 3.130912 | 1.324562 |
| H | -1.686585 | 4.336023 | -2.126816 |
| C | -1.329814 | 3.984133 | -1.162325 |
| O | 1.747826 | -0.653728 | 0.298469 |
| C | -1.445236 | 4.032503 | 1.248828 |
| H | -1.891861 | 4.421938 | 2.160000 |
| Cu | 0.765070 | -0.275271 | 2.053040 |
| Cu | 1.005821 | -0.330970 | -1.580065 |
| C | -1.935359 | 4.439770 | 0.008367 |
| H | -2.766810 | 5.134796 | -0.045385 |
| N | 1.086566 | 1.574621 | -2.479515 |
| N | 0.822091 | 1.673239 | 2.879961 |
| H | 1.050713 | -0.874414 | -4.717674 |
| H | 0.348682 | -0.578606 | 5.159012 |
| C | 1.563065 | -1.605657 | -4.088248 |
| C | 0.904219 | -1.387580 | 4.679360 |
| N | 1.825723 | -0.781135 | 3.677804 |
| N | 2.296349 | -0.863100 | -3.023533 |
| H | 2.258276 | -2.163412 | -4.725752 |
| H | 1.464555 | -1.915767 | 5.459143 |
| H | 2.535084 | -1.471039 | 3.424185 |
| H | 3.010908 | -1.476924 | -2.628776 |
| H | 1.234706 | 1.215323 | -4.556151 |
| H | 0.775178 | 1.476967 | 4.985837 |
| C | 1.892863 | 1.461440 | -3.714110 |
| C | 2.509268 | 0.448587 | 4.164829 |
| C | 1.514259 | 1.606347 | 4.185843 |
| C | 2.952361 | 0.380351 | -3.515025 |
| H | 3.321394 | 0.666655 | 3.465198 |
| H | 3.668755 | 0.696458 | -2.751248 |
| H | 2.945206 | 0.294930 | 5.159158 |
| H | 3.501600 | 0.199085 | -4.446531 |
| H | 2.384456 | 2.410019 | -3.954103 |
| H | 2.052389 | 2.537399 | 4.392208 |
| H | 1.117036 | 2.054371 | 0.202723 |

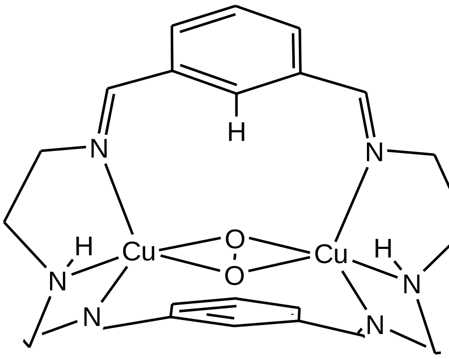


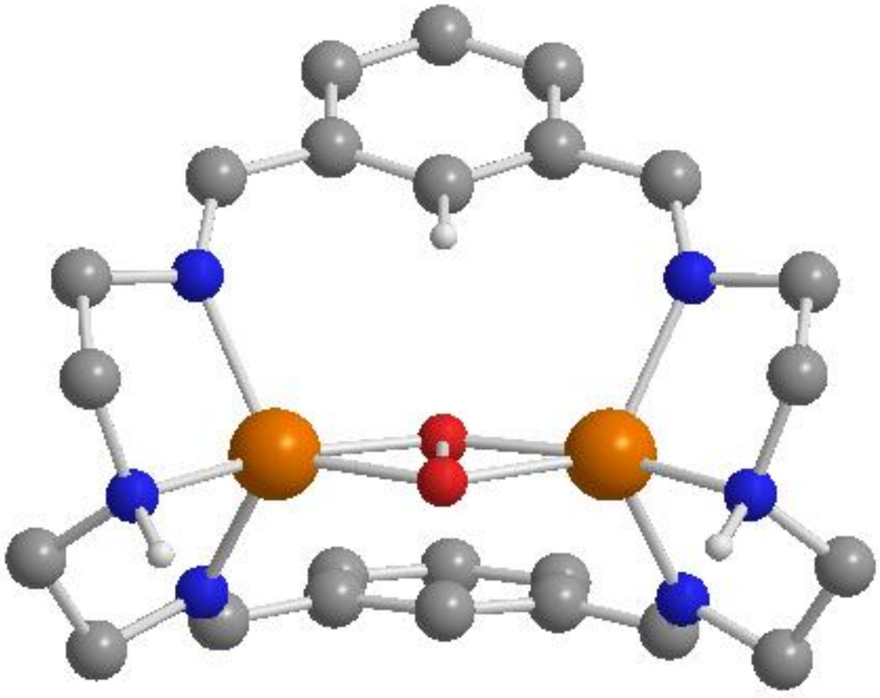

## TS-de

| | | | |
|---|---|---|---|
| H | -5.619533 | -0.816904 | -0.278169 |
| H | -4.262922 | -1.212280 | -2.310669 |
| C | -4.559411 | -1.029411 | -0.184608 |
| H | -4.601569 | -1.018738 | 1.969171 |
| C | -3.791411 | -1.243996 | -1.331824 |
| C | -3.982565 | -1.134430 | 1.083205 |
| C | -2.418106 | -1.519956 | -1.215641 |
| C | -2.609885 | -1.411280 | 1.209440 |
| H | -2.047221 | -2.443542 | -3.178719 |
| C | -1.843983 | -1.600309 | 0.055841 |
| H | -2.587450 | -2.147450 | 3.278276 |
| C | -1.568660 | -1.903880 | -2.350310 |
| C | -1.963040 | -1.698595 | 2.494183 |
| H | -0.810381 | -1.899776 | 0.149932 |
| N | -0.295758 | -1.728995 | -2.332000 |
| N | -0.697928 | -1.554788 | 2.676492 |
| H | -0.819460 | -2.493638 | 4.601242 |
| H | -0.058109 | -2.857224 | -4.140700 |
| C | -0.088898 | -2.226549 | 3.825269 |
| C | 0.521825 | -2.465606 | -3.293262 |
| H | 0.345900 | -3.165068 | 3.453793 |
| H | 0.939915 | -3.330425 | -2.759461 |
| C | 0.401005 | 2.604172 | -2.288902 |
| H | 0.353368 | 3.275369 | -3.154489 |
| C | 0.204454 | 2.706996 | 2.602795 |
| H | 0.093940 | 3.411516 | 3.435888 |
| O | 0.254055 | 0.367424 | 0.182671 |
| C | 0.225043 | 2.717053 | 0.152953 |
| C | -0.355469 | 2.979933 | -1.097807 |
| C | -0.453089 | 3.035498 | 1.339814 |
| H | -2.037197 | 3.887611 | -2.116217 |
| C | -1.591036 | 3.646375 | -1.155195 |
| O | 1.716719 | -1.039357 | 0.284081 |
| C | -1.688080 | 3.700891 | 1.267901 |
| H | -2.209194 | 3.984362 | 2.178336 |
| Cu | 0.898538 | -0.187335 | 1.765258 |
| Cu | 1.111407 | -0.210725 | -1.275879 |
| C | -2.250317 | 4.000629 | 0.024329 |
| H | -3.201938 | 4.519378 | -0.025646 |
| N | 1.162508 | 1.561583 | -2.338997 |
| N | 0.948198 | 1.661308 | 2.746413 |
| H | 1.235771 | -0.829734 | -4.483364 |
| H | 0.544436 | -0.507384 | 4.940900 |
| C | 1.650358 | -1.585508 | -3.813654 |
| C | 1.003533 | -1.355852 | 4.429927 |
| N | 1.903634 | -0.812954 | 3.366530 |
| N | 2.344102 | -0.870605 | -2.696490 |
| H | 2.375921 | -2.173929 | -4.386437 |
| H | 1.588923 | -1.913449 | 5.169795 |
| H | 2.543766 | -1.550388 | 3.064607 |
| H | 2.999332 | -1.513792 | -2.247568 |
| H | 1.512316 | 1.215580 | -4.402653 |
| H | 1.129705 | 1.489015 | 4.856456 |
| C | 2.080512 | 1.435958 | -3.491687 |
| C | 2.697198 | 0.358600 | 3.835713 |
| C | 1.771361 | 1.566728 | 3.971042 |
| C | 3.086868 | 0.331117 | -3.175236 |
| H | 3.465145 | 0.555520 | 3.081943 |
| H | 3.754424 | 0.650113 | -2.369423 |
| H | 3.198514 | 0.142279 | 4.786280 |
| H | 3.698770 | 0.093726 | -4.053152 |
| H | 2.614011 | 2.376764 | -3.661563 |
| H | 2.376944 | 2.471145 | 4.090972 |
| H | 1.252851 | 2.393101 | 0.202499 |

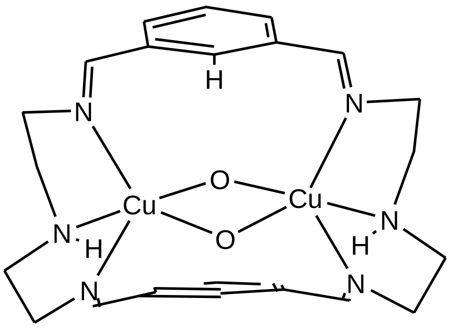


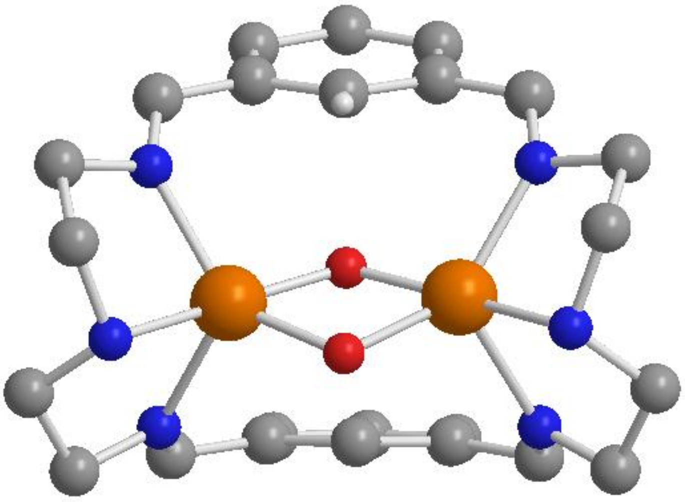

## e

| | | | |
|---|---|---|---|
| H | -5.757450 | -0.623141 | -0.032212 |
| H | -4.559969 | -0.920153 | -2.178045 |
| C | -4.694323 | -0.841243 | -0.028456 |
| H | -4.580973 | -0.947540 | 2.121385 |
| C | -4.015997 | -0.998591 | -1.240371 |
| C | -4.027923 | -1.014332 | 1.188093 |
| C | -2.639138 | -1.281624 | -1.239512 |
| C | -2.651215 | -1.297768 | 1.197105 |
| H | -2.408473 | -2.147958 | -3.248203 |
| C | -1.978415 | -1.420856 | -0.018781 |
| H | -2.444083 | -2.165821 | 3.207378 |
| C | -1.869742 | -1.642832 | -2.436055 |
| C | -1.894461 | -1.666992 | 2.398748 |
| H | -0.941893 | -1.725918 | -0.015259 |
| N | -0.591509 | -1.509704 | -2.486572 |
| N | -0.615767 | -1.540668 | 2.461737 |
| H | -0.564778 | -2.648609 | 4.294584 |
| H | -0.509963 | -2.627231 | -4.311967 |
| C | 0.098812 | -2.297272 | 3.492944 |
| C | 0.138943 | -2.277703 | -3.497502 |
| H | 0.510851 | -3.188256 | 2.999095 |
| H | 0.528942 | -3.169988 | -2.988451 |
| C | 0.013300 | 2.532301 | -2.491772 |
| H | -0.196410 | 3.168933 | -3.358176 |
| C | 0.055243 | 2.561825 | 2.494110 |
| H | -0.088179 | 3.262440 | 3.323898 |
| O | -0.104846 | 0.809891 | 0.013226 |
| C | -0.052724 | 2.236865 | 0.001267 |
| C | -0.668409 | 2.812995 | -1.259314 |
| C | -0.647728 | 2.823923 | 1.263942 |
| H | -2.129961 | 4.101890 | -2.143576 |
| C | -1.727529 | 3.699905 | -1.216734 |
| O | 1.720708 | -0.939603 | -0.020946 |
| C | -1.705508 | 3.713378 | 1.229541 |
| H | -2.093615 | 4.124548 | 2.158433 |
| Cu | 0.829239 | -0.090825 | 1.499358 |
| Cu | 0.856565 | -0.093617 | -1.460175 |
| C | -2.279499 | 4.124150 | 0.009230 |
| H | -3.118463 | 4.812071 | 0.013202 |
| N | 0.920185 | 1.604996 | -2.559655 |
| N | 0.903945 | 1.589761 | 2.602241 |
| H | 0.896887 | -0.696968 | -4.735241 |
| H | 0.805853 | -0.686666 | 4.720936 |
| C | 1.294179 | -1.467262 | -4.070920 |
| C | 1.229944 | -1.467969 | 4.086493 |
| N | 2.020846 | -0.805681 | 3.011898 |
| N | 2.059205 | -0.788585 | -2.988304 |
| H | 1.954617 | -2.105434 | -4.669225 |
| H | 1.877523 | -2.088216 | 4.717006 |
| H | 2.666170 | -1.480092 | 2.598077 |
| H | 2.710998 | -1.448327 | -2.561661 |
| H | 1.218243 | 1.362356 | -4.647680 |
| H | 1.233569 | 1.375951 | 4.682666 |
| C | 1.800393 | 1.528465 | -3.732761 |
| C | 2.785253 | 0.369213 | 3.509679 |
| C | 1.807221 | 1.521017 | 3.759123 |
| C | 2.803097 | 0.400275 | -3.481085 |
| H | 3.499830 | 0.650008 | 2.730559 |
| H | 3.507240 | 0.697022 | -2.698340 |
| H | 3.349045 | 0.129351 | 4.418961 |
| H | 3.376195 | 0.173118 | -4.387797 |
| H | 2.336062 | 2.475711 | -3.862201 |
| H | 2.362529 | 2.458898 | 3.870047 |
| H | 1.009492 | 2.544069 | -0.013933 |

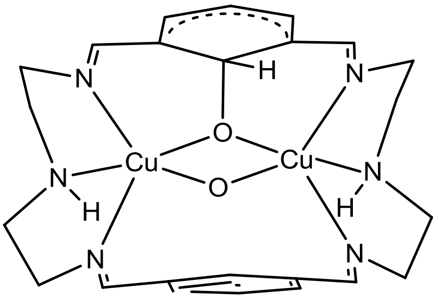


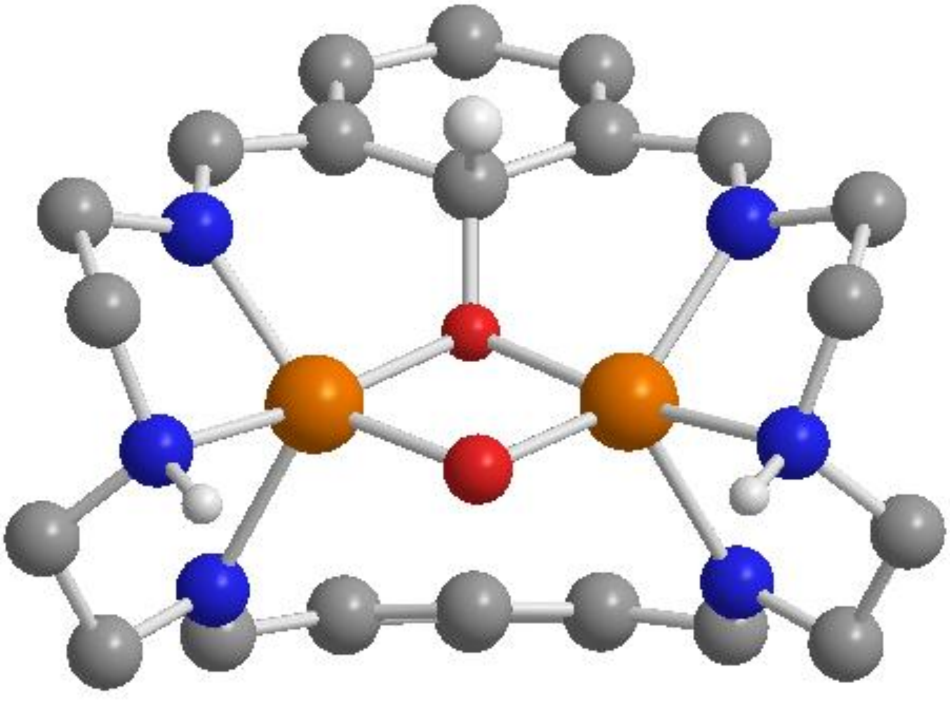

# TS-ef

| | | | |
|---|---|---|---|
| H | -5.482572 | -3.062660 | 0.114495 |
| H | -4.365506 | -2.862857 | -2.085679 |
| C | -4.503617 | -2.598874 | 0.051723 |
| H | -4.281994 | -2.438506 | 2.178567 |
| C | -3.875909 | -2.460902 | -1.201846 |
| C | -3.835821 | -2.234659 | 1.209156 |
| C | -2.642504 | -1.843517 | -1.336380 |
| C | -2.574349 | -1.597908 | 1.173212 |
| H | -2.425271 | -2.587027 | -3.357755 |
| C | -1.952422 | -1.279793 | -0.144703 |
| H | -2.481102 | -1.712515 | 3.316751 |
| C | -1.947015 | -1.940431 | -2.613673 |
| C | -1.877966 | -1.410213 | 2.453193 |
| H | -1.577320 | -2.396102 | 0.262596 |
| N | -0.796631 | -1.420850 | -2.853184 |
| N | -0.655204 | -1.053648 | 2.620471 |
| H | -0.172781 | -0.163418 | 4.443913 |
| H | -0.228157 | -1.020807 | -4.826778 |
| C | -0.135320 | -1.160104 | 3.994015 |
| C | -0.084646 | -1.805952 | -4.077507 |
| H | -0.751302 | -1.822341 | 4.614576 |
| H | -0.472768 | -2.739335 | -4.502522 |
| C | 0.029966 | 2.813099 | -2.414107 |
| H | 0.039005 | 3.555374 | -3.222842 |
| C | 0.587980 | 2.805301 | 2.488620 |
| H | 0.715646 | 3.531638 | 3.299970 |
| O | -1.048417 | -0.358645 | -0.227126 |
| C | 0.096160 | 2.712678 | 0.059598 |
| C | -0.399961 | 3.310250 | -1.101739 |
| C | -0.118321 | 3.303145 | 1.308727 |
| H | -1.534668 | 4.970278 | -1.901752 |
| C | -1.164591 | 4.486796 | -1.001309 |
| O | 1.547738 | -0.479832 | -0.138698 |
| C | -0.883938 | 4.481286 | 1.396288 |
| H | -1.033136 | 4.960530 | 2.360459 |
| Cu | 0.922385 | -0.099526 | 1.514845 |
| Cu | 0.399429 | -0.368751 | -1.548184 |
| C | -1.430458 | 5.050386 | 0.246863 |
| H | -2.023504 | 5.956241 | 0.318549 |
| N | 0.459317 | 1.623755 | -2.625682 |
| N | 1.114573 | 1.635449 | 2.605863 |
| H | 1.997992 | -2.080649 | -4.636076 |
| H | 1.730021 | -1.678963 | 4.966628 |
| C | 1.401691 | -1.962977 | -3.722684 |
| C | 1.305358 | -1.692696 | 3.954127 |
| N | 2.144556 | -0.928433 | 3.000040 |
| N | 1.871259 | -0.814384 | -2.909230 |
| H | 1.532530 | -2.864941 | -3.116856 |
| H | 1.286907 | -2.733567 | 3.617619 |
| H | 2.816679 | -1.561267 | 2.572264 |
| H | 2.641238 | -1.108846 | -2.310953 |
| H | 0.360891 | 0.889274 | -4.591103 |
| H | 1.313847 | 1.228479 | 4.675243 |
| C | 1.093161 | 1.342714 | -3.914235 |
| C | 2.886745 | 0.224322 | 3.561610 |
| C | 1.952963 | 1.410813 | 3.803196 |
| C | 2.277727 | 0.396562 | -3.670511 |
| H | 3.644717 | 0.503047 | 2.823345 |
| H | 3.025816 | 0.918337 | -3.067520 |
| H | 3.402402 | -0.040022 | 4.494431 |
| H | 2.744726 | 0.117943 | -4.623509 |
| H | 1.459020 | 2.254283 | -4.403160 |
| H | 2.552914 | 2.298904 | 4.029759 |
| H | 0.715649 | 1.828085 | -0.012088 |

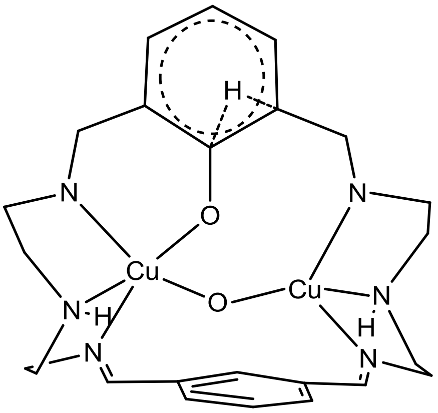


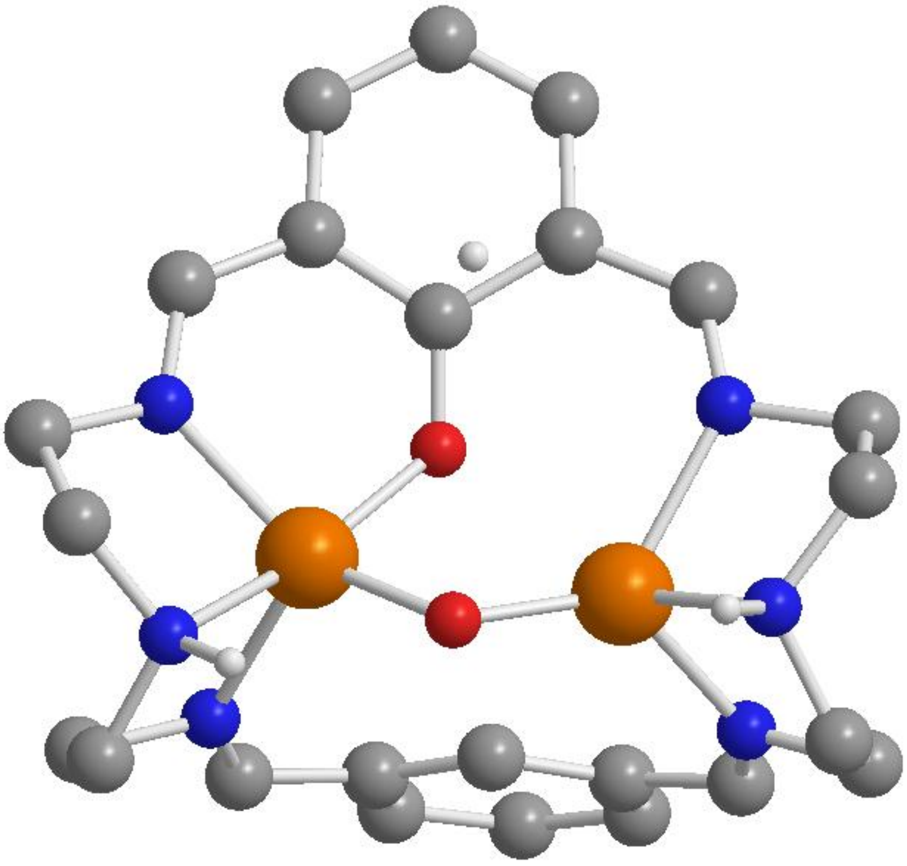

**f**

| | | | |
|---|---|---|---|
| H | -5.622067 | -2.932006 | -0.142056 |
| H | -4.304218 | -2.969200 | -2.249336 |
| C | -4.575062 | -2.650075 | -0.112838 |
| H | -4.473320 | -2.496673 | 2.007425 |
| C | -3.826934 | -2.602280 | -1.342401 |
| C | -3.949649 | -2.402203 | 1.059484 |
| C | -2.550857 | -2.094147 | -1.422049 |
| C | -2.474718 | -2.106043 | 1.091659 |
| H | -2.225091 | -2.818556 | -3.432057 |
| C | -1.957897 | -1.509477 | -0.210933 |
| H | -2.656517 | -1.659499 | 3.249074 |
| C | -1.792503 | -2.171895 | -2.660538 |
| C | -1.952308 | -1.563959 | 2.409011 |
| H | -2.014193 | -3.120180 | 1.029828 |
| N | -0.641993 | -1.627858 | -2.854937 |
| N | -0.761768 | -1.159300 | 2.580420 |
| H | -0.298501 | 0.215318 | 4.077012 |
| H | -0.162678 | -1.269335 | -4.873083 |
| C | -0.332050 | -0.871095 | 3.946735 |
| C | 0.099673 | -1.976678 | -4.078935 |
| H | -1.019895 | -1.273725 | 4.704195 |
| H | -0.162401 | -2.979379 | -4.434968 |
| C | -0.128477 | 2.826424 | -2.361786 |
| H | -0.198898 | 3.576592 | -3.160288 |
| C | 0.875397 | 2.795757 | 2.510132 |
| H | 0.974869 | 3.497267 | 3.346090 |
| O | -1.054086 | -0.672062 | -0.232930 |
| C | 0.239911 | 2.730268 | 0.093187 |
| C | -0.391532 | 3.322732 | -1.004138 |
| C | 0.125151 | 3.304595 | 1.365819 |
| H | -1.633790 | 4.962763 | -1.674824 |
| C | -1.161743 | 4.485897 | -0.819644 |
| O | 1.622512 | -0.247632 | -0.220273 |
| C | -0.650038 | 4.468776 | 1.536402 |
| H | -0.722153 | 4.931948 | 2.517160 |
| Cu | 1.368789 | -0.015814 | 1.505345 |
| Cu | 0.420069 | -0.405508 | -1.620239 |
| C | -1.309238 | 5.041552 | 0.450734 |
| H | -1.906022 | 5.937381 | 0.587309 |
| N | 0.233062 | 1.628571 | -2.637928 |
| N | 1.436918 | 1.638696 | 2.615345 |
| H | 2.189438 | -2.001981 | -4.673764 |
| H | 1.399109 | -1.300245 | 5.180721 |
| C | 1.595644 | -1.899946 | -3.757059 |
| C | 1.059958 | -1.481339 | 4.153862 |
| N | 2.061935 | -0.959587 | 3.176051 |
| N | 1.890377 | -0.633276 | -3.048405 |
| H | 1.866777 | -2.720509 | -3.085449 |
| H | 0.993021 | -2.564265 | 4.017139 |
| H | 2.637702 | -1.743017 | 2.871426 |
| H | 2.732789 | -0.738752 | -2.485677 |
| H | -0.071078 | 0.767377 | -4.520763 |
| H | 1.553563 | 1.379868 | 4.716607 |
| C | 0.692844 | 1.353050 | -3.998500 |
| C | 2.977437 | 0.083765 | 3.713766 |
| C | 2.225252 | 1.403704 | 3.849774 |
| C | 2.016017 | 0.575030 | -3.908384 |
| H | 3.796995 | 0.194273 | 2.996327 |
| H | 2.772157 | 1.222109 | -3.455863 |
| H | 3.409358 | -0.213648 | 4.676786 |
| H | 2.364561 | 0.303092 | -4.912252 |
| H | 0.854659 | 2.270234 | -4.579519 |
| H | 2.939589 | 2.217614 | 4.011300 |
| H | 0.878498 | 1.866754 | -0.055825 |

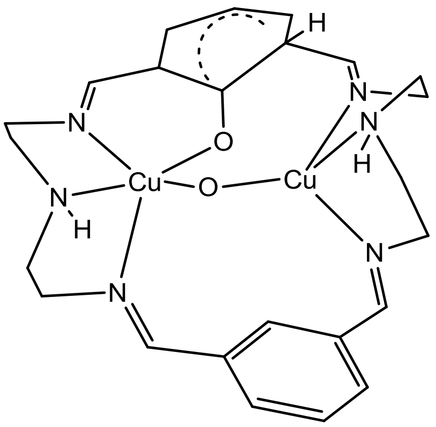


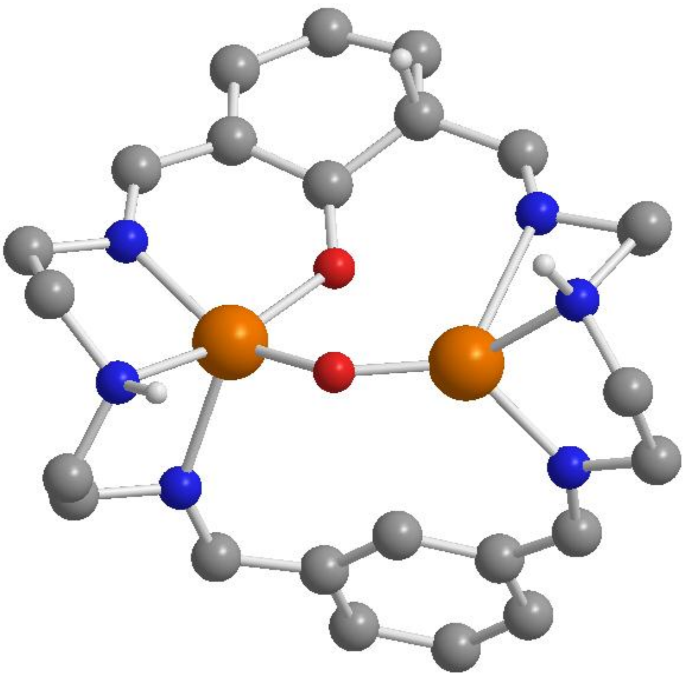

## TS-fg

| | | | |
|---|---|---|---|
| H | -5.011890 | -3.244555 | -1.442555 |
| H | -3.003436 | -3.537361 | -2.865107 |
| C | -4.049932 | -2.873534 | -1.104342 |
| H | -4.797342 | -2.245330 | 0.814809 |
| C | -2.921631 | -3.026241 | -1.909281 |
| C | -3.930406 | -2.297040 | 0.161358 |
| C | -1.676340 | -2.531045 | -1.477893 |
| C | -2.686562 | -1.818369 | 0.609259 |
| H | -0.493832 | -3.598073 | -2.972871 |
| C | -1.572281 | -1.914045 | -0.228519 |
| H | -3.199628 | -1.866738 | 2.730504 |
| C | -0.471572 | -2.752433 | -2.274866 |
| C | -2.476945 | -1.454336 | 2.015237 |
| H | -0.604298 | -1.580846 | 0.125280 |
| N | 0.588398 | -2.026014 | -2.195659 |
| N | -1.444888 | -0.823937 | 2.436855 |
| H | -1.175896 | 0.093437 | 4.300817 |
| H | 1.644839 | -3.388821 | -3.460690 |
| C | -1.120398 | -0.904249 | 3.856922 |
| C | 1.714385 | -2.364029 | -3.077348 |
| H | -1.799738 | -1.561029 | 4.415425 |
| H | 2.640919 | -2.284567 | -2.497636 |
| C | -0.619659 | 2.572411 | -1.829420 |
| H | -1.350178 | 3.239954 | -2.299241 |
| C | 0.510967 | 3.223702 | 3.065732 |
| H | 0.913142 | 3.914563 | 3.815218 |
| O | -1.124410 | 1.951722 | 1.020406 |
| C | -0.388860 | 2.925932 | 0.733923 |
| C | 0.153674 | 3.075434 | -0.639383 |
| C | 0.229191 | 3.771133 | 1.746804 |
| H | 1.048802 | 4.581486 | -1.992332 |
| C | 0.801493 | 4.368924 | -0.955306 |
| O | 1.048551 | 0.495724 | 0.035504 |
| C | 0.849363 | 4.947326 | 1.378739 |
| H | 1.247847 | 5.601273 | 2.152181 |
| Cu | 0.166745 | 0.535659 | 1.743049 |
| Cu | 0.838880 | -0.027029 | -1.692656 |
| C | 1.087224 | 5.282027 | 0.010541 |
| H | 1.564957 | 6.224300 | -0.235285 |
| N | -0.310067 | 1.461896 | -2.369806 |
| N | 0.514799 | 1.955286 | 3.275507 |
| H | 0.873628 | -1.599510 | -4.909035 |
| H | 0.663286 | -1.541139 | 4.947813 |
| C | 1.705581 | -1.365022 | -4.238781 |
| C | 0.310126 | -1.469808 | 3.912794 |
| N | 1.251234 | -0.641569 | 3.091798 |
| N | 1.508053 | 0.018787 | -3.707108 |
| H | 2.630605 | -1.433360 | -4.820080 |
| H | 0.299568 | -2.479731 | 3.494054 |
| H | 1.876395 | -1.261633 | 2.579570 |
| H | 2.414627 | 0.483708 | -3.701890 |
| H | -1.312601 | 0.148268 | -3.651824 |
| H | 0.499981 | 1.054044 | 5.180547 |
| C | -0.744116 | 1.083257 | -3.712433 |
| C | 2.088784 | 0.275974 | 3.913808 |
| C | 1.218472 | 1.422270 | 4.440352 |
| C | 0.564384 | 0.876741 | -4.499625 |
| H | 2.872993 | 0.675589 | 3.263808 |
| H | 1.038258 | 1.848341 | -4.665744 |
| H | 2.566752 | -0.261994 | 4.741530 |
| H | 0.361664 | 0.435895 | -5.479960 |
| H | -1.375214 | 1.843052 | -4.188634 |
| H | 1.840707 | 2.177773 | 4.934638 |
| H | 0.908951 | 2.159109 | -0.417317 |

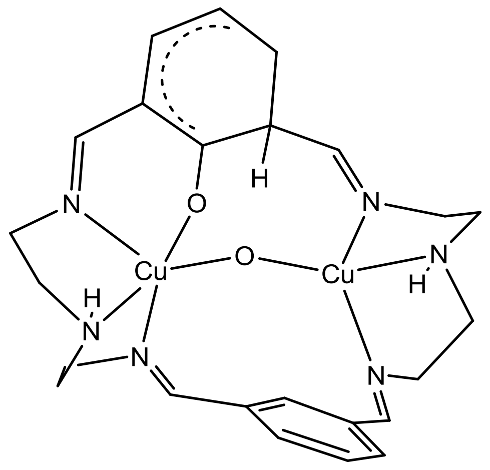

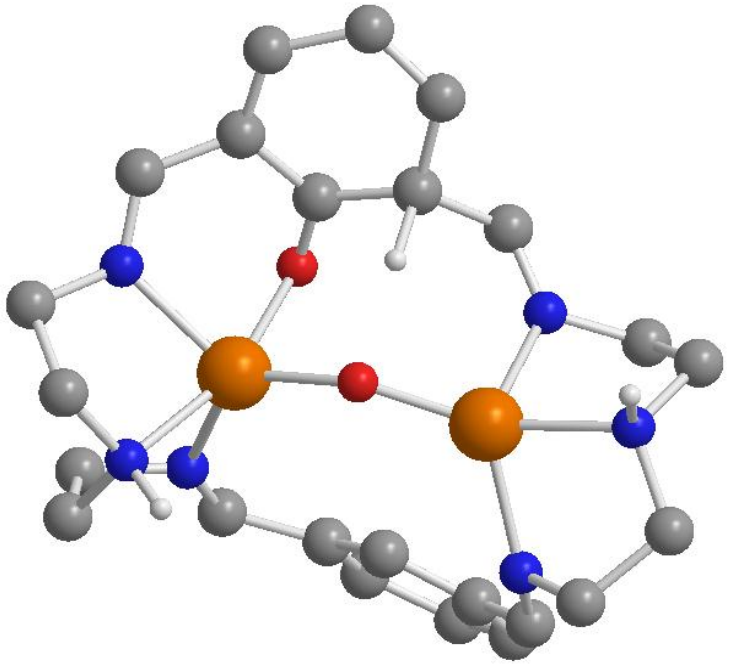

## g

| | | | |
|---|---|---|---|
| H | -5.703534 | -1.411600 | -0.001468 |
| H | -4.488418 | -1.611146 | -2.148410 |
| C | -4.623734 | -1.520414 | 0.000429 |
| H | -4.494252 | -1.594783 | 2.150269 |
| C | -3.936441 | -1.621187 | -1.212232 |
| C | -3.939758 | -1.611954 | 1.215686 |
| C | -2.537825 | -1.761192 | -1.214935 |
| C | -2.541145 | -1.751881 | 1.223214 |
| H | -2.252508 | -2.590982 | -3.234370 |
| C | -1.860533 | -1.812663 | 0.005274 |
| H | -2.260803 | -2.566748 | 3.249470 |
| C | -1.748086 | -2.054113 | -2.420129 |
| C | -1.754540 | -2.035552 | 2.432659 |
| H | -0.796206 | -2.000707 | 0.007444 |
| N | -0.488609 | -1.806976 | -2.473136 |
| N | -0.495327 | -1.787379 | 2.487089 |
| H | -0.327731 | -2.864302 | 4.331735 |
| H | -0.317253 | -2.896552 | -4.310005 |
| C | 0.294352 | -2.418198 | 3.543436 |
| C | 0.303206 | -2.445624 | -3.523174 |
| H | 0.852878 | -3.239387 | 3.072034 |
| H | 0.859969 | -3.264011 | -3.044860 |
| C | -0.425977 | 2.402795 | -2.458522 |
| H | -0.596076 | 3.148130 | -3.242684 |
| C | -0.437511 | 2.422047 | 2.437196 |
| H | -0.611281 | 3.173556 | 3.214624 |
| O | -0.004090 | 0.923003 | -0.003634 |
| C | -0.770185 | 1.991614 | -0.009772 |
| C | -1.159326 | 2.621255 | -1.233642 |
| C | -1.165028 | 2.630943 | 1.207192 |
| H | -2.409066 | 4.110805 | -2.156413 |
| C | -2.111907 | 3.659696 | -1.212841 |
| O | 1.590110 | -0.972497 | 0.007156 |
| C | -2.117476 | 3.669175 | 1.173725 |
| H | -2.419051 | 4.127724 | 2.112295 |
| Cu | 0.770685 | -0.079074 | 1.550218 |
| Cu | 0.776532 | -0.091497 | -1.546069 |
| C | -2.644638 | 4.142436 | -0.022693 |
| H | -3.387722 | 4.931657 | -0.027566 |
| N | 0.474595 | 1.495827 | -2.654499 |
| N | 0.462029 | 1.516613 | 2.644759 |
| H | 0.718711 | -0.759052 | -4.778934 |
| H | 0.705871 | -0.723815 | 4.790143 |
| C | 1.278803 | -1.455940 | -4.152512 |
| C | 1.267743 | -1.423642 | 4.168622 |
| N | 1.986376 | -0.633744 | 3.128650 |
| N | 1.996332 | -0.660798 | -3.115860 |
| H | 1.992015 | -1.977756 | -4.801078 |
| H | 1.980350 | -1.940917 | 4.821462 |
| H | 2.775344 | -1.178059 | 2.778399 |
| H | 2.782652 | -1.204799 | -2.759202 |
| H | 0.743819 | 1.313466 | -4.746238 |
| H | 0.721697 | 1.348008 | 4.738768 |
| C | 1.323931 | 1.580308 | -3.855103 |
| C | 2.492389 | 0.664925 | 3.654912 |
| C | 1.305440 | 1.610880 | 3.848819 |
| C | 2.507659 | 0.632736 | -3.649580 |
| H | 3.179691 | 1.075430 | 2.908962 |
| H | 3.193124 | 1.046881 | -2.903951 |
| H | 3.045337 | 0.533213 | 4.592536 |
| H | 3.063951 | 0.492831 | -4.584031 |
| H | 1.691743 | 2.601389 | -4.001585 |
| H | 1.669981 | 2.633772 | 3.990730 |
| H | 1.627459 | -1.940211 | 0.011207 |

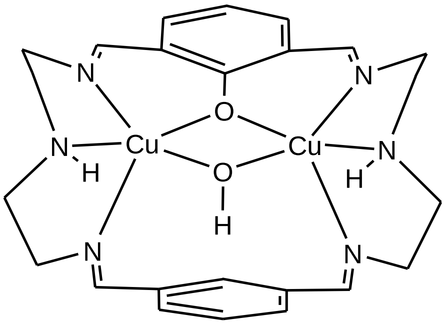


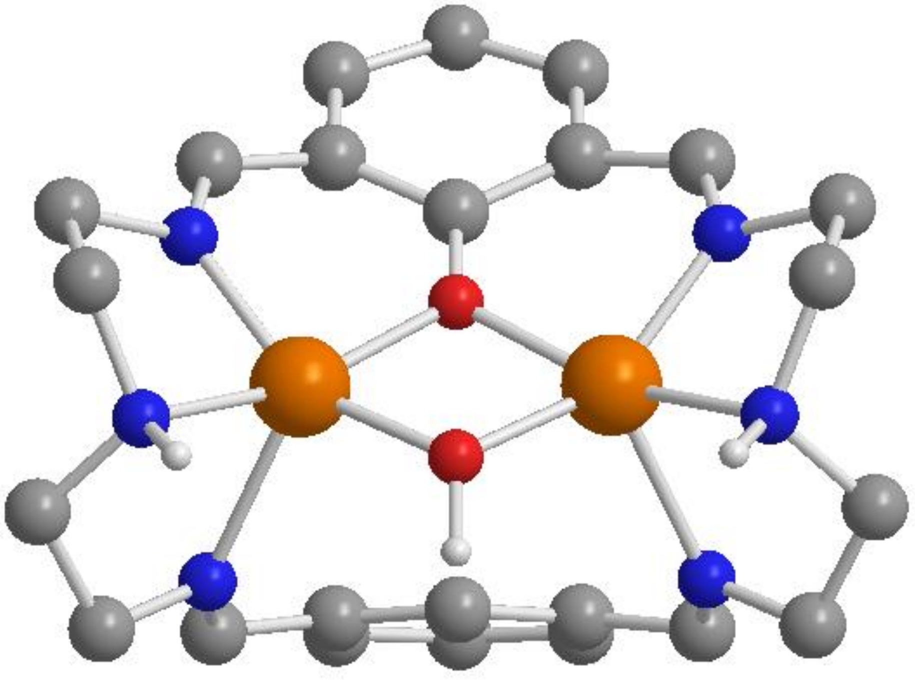

# TS-di

| | | | |
|---|---|---|---|
| H | -5.601690 | -0.809114 | -0.241959 |
| H | -4.253690 | -1.189784 | -2.283195 |
| C | -4.541395 | -1.023557 | -0.154906 |
| H | -4.574142 | -1.036355 | 1.998850 |
| C | -3.778265 | -1.229062 | -1.306521 |
| C | -3.958989 | -1.142287 | 1.108981 |
| C | -2.404462 | -1.507088 | -1.199114 |
| C | -2.586086 | -1.421158 | 1.227350 |
| H | -2.067913 | -2.417941 | -3.171030 |
| C | -1.823149 | -1.597932 | 0.069092 |
| H | -2.568189 | -2.183187 | 3.286514 |
| C | -1.571499 | -1.887323 | -2.347322 |
| C | -1.941194 | -1.722702 | 2.511083 |
| H | -0.788560 | -1.897352 | 0.156746 |
| N | -0.297449 | -1.720093 | -2.353238 |
| N | -0.678392 | -1.574489 | 2.699995 |
| H | -0.807923 | -2.535718 | 4.612953 |
| H | -0.114229 | -2.831370 | -4.178325 |
| C | -0.072224 | -2.247194 | 3.849499 |
| C | 0.491446 | -2.452463 | -3.343376 |
| H | 0.384097 | -3.173842 | 3.473884 |
| H | 0.916536 | -3.325893 | -2.829328 |
| C | 0.440949 | 2.612267 | -2.299852 |
| H | 0.404355 | 3.297420 | -3.155162 |
| C | 0.189572 | 2.708448 | 2.601837 |
| H | 0.069982 | 3.424892 | 3.423497 |
| O | 0.365181 | 0.311976 | 0.192640 |
| C | 0.245980 | 2.723496 | 0.146729 |
| C | -0.322873 | 2.981712 | -1.108196 |
| C | -0.448202 | 3.030863 | 1.325224 |
| H | -1.998498 | 3.881362 | -2.144269 |
| C | -1.562002 | 3.641158 | -1.178564 |
| O | 1.647947 | -1.021289 | 0.293734 |
| C | -1.686871 | 3.689531 | 1.241903 |
| H | -2.219592 | 3.966706 | 2.147541 |
| Cu | 0.920310 | -0.183102 | 1.818683 |
| Cu | 1.125827 | -0.230551 | -1.332782 |
| C | -2.237004 | 3.988780 | -0.006407 |
| H | -3.191294 | 4.501645 | -0.066028 |
| N | 1.187192 | 1.561048 | -2.363908 |
| N | 0.921197 | 1.659765 | 2.776173 |
| H | 1.186407 | -0.805391 | -4.529487 |
| H | 0.514949 | -0.526321 | 4.986886 |
| C | 1.613702 | -1.576269 | -3.885155 |
| C | 0.996856 | -1.364708 | 4.480119 |
| N | 1.911060 | -0.802590 | 3.439448 |
| N | 2.345166 | -0.889108 | -2.776362 |
| H | 2.312884 | -2.163888 | -4.490834 |
| H | 1.573057 | -1.919199 | 5.229606 |
| H | 2.577766 | -1.525276 | 3.161570 |
| H | 3.007201 | -1.547597 | -2.361648 |
| H | 1.508748 | 1.240065 | -4.434590 |
| H | 1.037346 | 1.484581 | 4.888340 |
| C | 2.091943 | 1.437820 | -3.527921 |
| C | 2.662957 | 0.389381 | 3.924503 |
| C | 1.708162 | 1.578339 | 4.026495 |
| C | 3.088736 | 0.314230 | -3.247208 |
| H | 3.449126 | 0.600210 | 3.193504 |
| H | 3.774150 | 0.611899 | -2.448148 |
| H | 3.140525 | 0.192058 | 4.891507 |
| H | 3.683007 | 0.089752 | -4.140780 |
| H | 2.637037 | 2.373260 | -3.690693 |
| H | 2.290238 | 2.494725 | 4.168940 |
| H | 1.266839 | 2.379696 | 0.206706 |

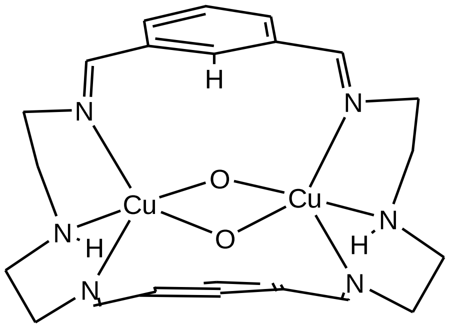


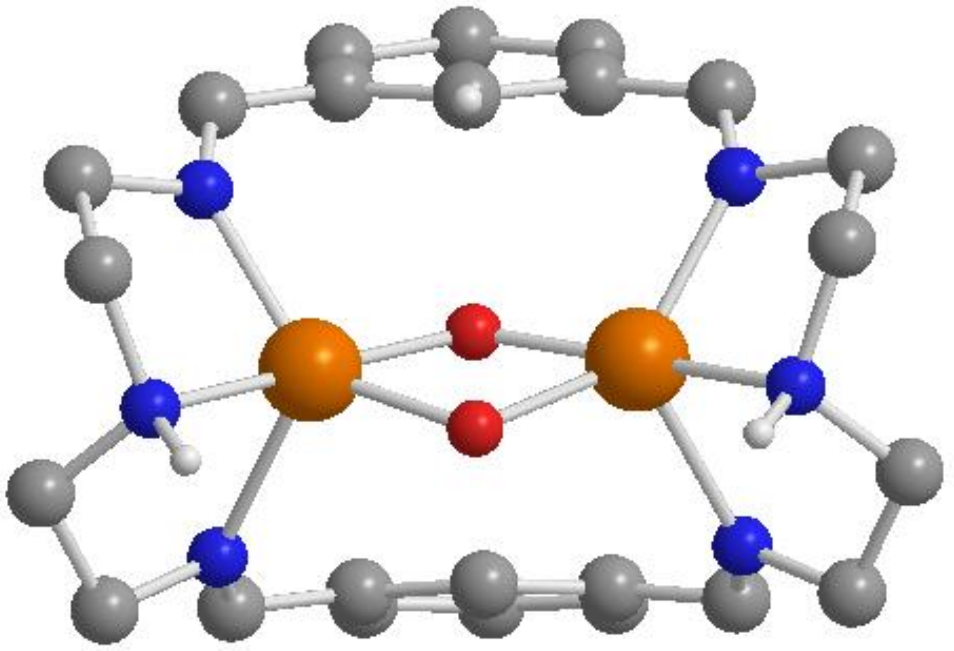

## i

| | | | |
|---|---|---|---|
| H | -5.655235 | -0.772923 | -0.241963 |
| H | -4.310428 | -1.142549 | -2.286320 |
| C | -4.593883 | -0.982892 | -0.156300 |
| H | -4.627543 | -0.998486 | 1.998082 |
| C | -3.832280 | -1.182301 | -1.310985 |
| C | -4.011374 | -1.100805 | 1.108494 |
| C | -2.457575 | -1.454711 | -1.204643 |
| C | -2.637450 | -1.374109 | 1.224122 |
| H | -2.077902 | -2.374995 | -3.170439 |
| C | -1.879031 | -1.544902 | 0.063525 |
| H | -2.572142 | -2.151526 | 3.282598 |
| C | -1.604143 | -1.838317 | -2.337169 |
| C | -1.967811 | -1.683357 | 2.493696 |
| H | -0.843784 | -1.841383 | 0.149545 |
| N | -0.329662 | -1.681490 | -2.296388 |
| N | -0.697988 | -1.546056 | 2.636288 |
| H | -0.730288 | -2.533700 | 4.540962 |
| H | -0.055892 | -2.814457 | -4.097823 |
| C | -0.039274 | -2.244095 | 3.736612 |
| C | 0.500666 | -2.438509 | -3.227518 |
| H | 0.381145 | -3.171031 | 3.322138 |
| H | 0.875960 | -3.314329 | -2.680153 |
| C | 0.395833 | 2.574177 | -2.287568 |
| H | 0.342485 | 3.210895 | -3.178462 |
| C | 0.144732 | 2.673827 | 2.584651 |
| H | 0.006223 | 3.345517 | 3.440154 |
| O | 0.226588 | 0.506616 | 0.181557 |
| C | 0.200303 | 2.715554 | 0.143492 |
| C | -0.377125 | 2.964030 | -1.116722 |
| C | -0.503320 | 3.015668 | 1.325803 |
| H | -2.070016 | 3.830079 | -2.150370 |
| C | -1.625639 | 3.603046 | -1.185069 |
| O | 1.744992 | -1.092033 | 0.300575 |
| C | -1.751006 | 3.653713 | 1.238514 |
| H | -2.291883 | 3.919641 | 2.142671 |
| Cu | 0.935389 | -0.150463 | 1.668439 |
| Cu | 1.124198 | -0.190014 | -1.182504 |
| C | -2.302892 | 3.946855 | -0.011766 |
| H | -3.263783 | 4.446842 | -0.072085 |
| N | 1.187385 | 1.549464 | -2.287996 |
| N | 0.925083 | 1.647641 | 2.701786 |
| H | 1.303620 | -0.828571 | -4.396058 |
| H | 0.636579 | -0.554806 | 4.869807 |
| C | 1.670903 | -1.589751 | -3.705834 |
| C | 1.076271 | -1.384730 | 4.314402 |
| N | 1.938902 | -0.801572 | 3.237054 |
| N | 2.347337 | -0.882351 | -2.570497 |
| H | 2.405241 | -2.199302 | -4.244235 |
| H | 1.698700 | -1.959035 | 5.009823 |
| H | 2.578537 | -1.520872 | 2.891768 |
| H | 2.971581 | -1.535629 | -2.091741 |
| H | 1.615603 | 1.197378 | -4.340175 |
| H | 1.148815 | 1.434324 | 4.807517 |
| C | 2.146232 | 1.412265 | -3.405582 |
| C | 2.723092 | 0.371181 | 3.722661 |
| C | 1.769950 | 1.549678 | 3.911860 |
| C | 3.127324 | 0.300248 | -3.040612 |
| H | 3.469258 | 0.606696 | 2.958429 |
| H | 3.772685 | 0.620141 | -2.217401 |
| H | 3.248886 | 0.133519 | 4.654513 |
| H | 3.763624 | 0.036380 | -3.893064 |
| H | 2.694367 | 2.348597 | -3.552051 |
| H | 2.351784 | 2.468473 | 4.039033 |
| H | 1.251735 | 2.479495 | 0.203144 |

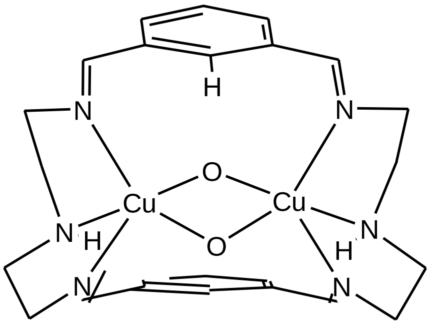

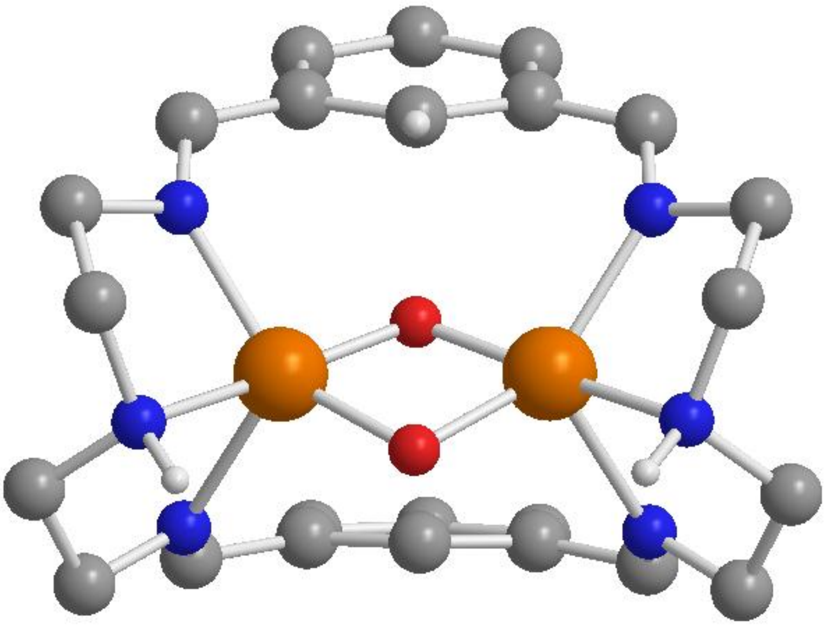

## TS-fh

| | | | |
|---|---|---|---|
| H | -5.724692 | -1.785997 | -0.772417 |
| H | -4.185736 | -2.168445 | -2.676102 |
| C | -4.652716 | -1.809225 | -0.605202 |
| H | -4.831294 | -1.597175 | 1.530206 |
| C | -3.785220 | -2.012045 | -1.677618 |
| C | -4.146462 | -1.691528 | 0.691330 |
| C | -2.393146 | -2.038371 | -1.462974 |
| C | -2.759820 | -1.727800 | 0.918965 |
| H | -1.932789 | -2.858143 | -3.432655 |
| C | -1.892381 | -1.878166 | -0.165374 |
| H | -2.865840 | -2.216728 | 3.052618 |
| C | -1.488720 | -2.360294 | -2.563644 |
| C | -2.196897 | -1.815390 | 2.277713 |
| H | -0.829781 | -1.961206 | 0.025379 |
| N | -0.219354 | -2.133843 | -2.558899 |
| N | -0.965001 | -1.572780 | 2.525423 |
| H | -1.194313 | -2.175699 | 4.571682 |
| H | 0.012065 | -3.123676 | -4.440974 |
| C | -0.427949 | -2.066450 | 3.790583 |
| C | 0.608170 | -2.626142 | -3.667099 |
| H | -0.027764 | -3.071118 | 3.591580 |
| H | 1.304657 | -3.369216 | -3.258122 |
| C | -0.798500 | 2.238524 | -2.113682 |
| H | -1.644793 | 1.666553 | -2.495804 |
| C | 0.767350 | 2.873219 | 2.581567 |
| H | 1.054017 | 3.653773 | 3.295252 |
| O | -0.131988 | 1.181256 | 0.332464 |
| C | -0.307036 | 2.437280 | 0.339389 |
| C | -0.715700 | 3.106432 | -0.893619 |
| C | -0.030403 | 3.315798 | 1.457392 |
| H | -1.721758 | 4.854830 | -1.738408 |
| C | -1.262776 | 4.436034 | -0.846870 |
| O | 1.457295 | -1.076411 | 0.268510 |
| C | -0.469147 | 4.637996 | 1.411715 |
| H | -0.289474 | 5.272364 | 2.277190 |
| Cu | 0.832554 | 0.048381 | 1.632166 |
| Cu | 0.973869 | -1.106283 | -1.398536 |
| C | -1.128575 | 5.192015 | 0.294043 |
| H | -1.485002 | 6.215371 | 0.326447 |
| N | 0.371758 | 2.296354 | -2.635722 |
| N | 1.215460 | 1.674888 | 2.741844 |
| H | 0.677683 | -0.838014 | -4.855169 |
| H | 0.277823 | -0.219315 | 4.627212 |
| C | 1.369358 | -1.439408 | -4.261179 |
| C | 0.695724 | -1.174622 | 4.298736 |
| N | 1.679896 | -0.896973 | 3.224086 |
| N | 1.911984 | -0.578852 | -3.159622 |
| H | 2.170779 | -1.775291 | -4.928247 |
| H | 1.185606 | -1.639206 | 5.164421 |
| H | 2.084242 | -1.769594 | 2.881129 |
| H | 2.845309 | -0.933607 | -2.936500 |
| H | 0.060017 | 1.062391 | -4.311765 |
| H | 1.620207 | 1.483303 | 4.807348 |
| C | 0.861447 | 1.651481 | -3.850417 |
| C | 2.758540 | 0.027030 | 3.648296 |
| C | 2.153537 | 1.419473 | 3.852150 |
| C | 2.138021 | 0.827479 | -3.612952 |
| H | 3.502190 | 0.049686 | 2.845897 |
| H | 2.754236 | 1.327183 | -2.860372 |
| H | 3.255035 | -0.314591 | 4.564758 |
| H | 2.707520 | 0.812253 | -4.550434 |
| H | 1.115411 | 2.460054 | -4.544712 |
| H | 2.948431 | 2.172848 | 3.872041 |
| H | 0.559534 | 3.166012 | -1.536639 |

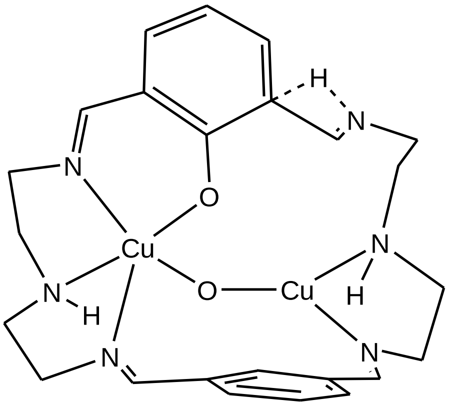


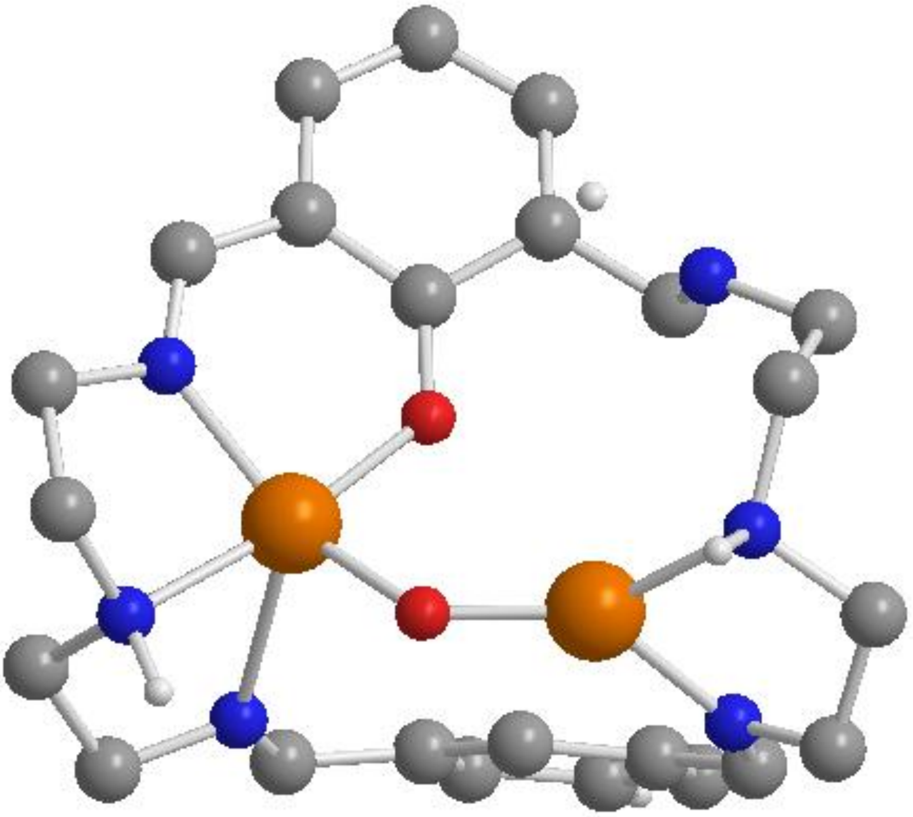

## h

| | | | |
|---|---|---|---|
| H | -5.269504 | -0.415340 | -0.091381 |
| H | -4.194314 | -1.040492 | -2.231054 |
| C | -4.225183 | -0.706047 | -0.109559 |
| H | -3.987867 | -0.619345 | 2.028500 |
| C | -3.610635 | -1.043159 | -1.314004 |
| C | -3.500932 | -0.807598 | 1.075039 |
| C | -2.251728 | -1.409414 | -1.378990 |
| C | -2.145623 | -1.181002 | 1.060022 |
| H | -2.462427 | -2.207253 | -3.375386 |
| C | -1.497809 | -1.420286 | -0.182000 |
| H | -1.979279 | -1.436277 | 3.228331 |
| C | -1.715940 | -1.941514 | -2.619070 |
| C | -1.430715 | -1.483557 | 2.282256 |
| H | 0.099803 | -1.879446 | 0.913810 |
| N | -0.471266 | -2.187114 | -2.855435 |
| N | -0.198858 | -1.885496 | 2.250932 |
| H | -0.234995 | -2.286002 | 4.319317 |
| H | -0.202891 | -2.255526 | -4.956923 |
| C | 0.428312 | -2.422652 | 3.455556 |
| C | -0.121127 | -2.920201 | -4.089482 |
| H | 0.546844 | -3.506207 | 3.315214 |
| H | -0.810510 | -3.755140 | -4.253381 |
| C | 1.792030 | 1.665877 | -2.780713 |
| H | 1.859276 | 2.327589 | -3.654739 |
| C | 3.504644 | 1.546821 | 1.895826 |
| H | 3.899233 | 2.190072 | 2.689782 |
| O | -0.196037 | -1.731497 | -0.178192 |
| C | 2.539669 | 1.599934 | -0.414622 |
| C | 1.999890 | 2.321312 | -1.480478 |
| C | 2.873955 | 2.246859 | 0.783282 |
| H | 1.384949 | 4.275639 | -2.177803 |
| C | 1.783071 | 3.705166 | -1.342622 |
| O | 2.526252 | -1.777855 | -0.576760 |
| C | 2.645580 | 3.631864 | 0.909063 |
| H | 2.918793 | 4.144021 | 1.828035 |
| Cu | 2.832392 | -1.230394 | 1.038580 |
| Cu | 1.115043 | -1.654461 | -1.769073 |
| C | 2.087810 | 4.352227 | -0.145408 |
| H | 1.916691 | 5.418899 | -0.044367 |
| N | 1.612966 | 0.407234 | -2.937233 |
| N | 3.635700 | 0.268420 | 2.024556 |
| H | 1.686009 | -3.823089 | -4.892231 |
| H | 1.688797 | -0.709018 | 3.832670 |
| C | 1.307339 | -3.446499 | -3.934106 |
| C | 1.780982 | -1.797952 | 3.797275 |
| N | 2.852778 | -2.148290 | 2.826961 |
| N | 2.174037 | -2.378470 | -3.380875 |
| H | 1.317692 | -4.274164 | -3.217697 |
| H | 2.068773 | -2.143715 | 4.799277 |
| H | 2.852635 | -3.162847 | 2.706625 |
| H | 3.003515 | -2.795468 | -2.961880 |
| H | 0.643548 | -0.378056 | -4.614115 |
| H | 3.910629 | 0.247992 | 4.128919 |
| C | 1.657248 | -0.103835 | -4.306541 |
| C | 4.213383 | -1.744136 | 3.290476 |
| C | 4.338559 | -0.218654 | 3.234168 |
| C | 2.590204 | -1.323971 | -4.344580 |
| H | 4.933443 | -2.210614 | 2.610641 |
| H | 3.597324 | -1.002970 | -4.065412 |
| H | 4.419439 | -2.112613 | 4.302244 |
| H | 2.637909 | -1.728954 | -5.363108 |
| H | 2.024267 | 0.645067 | -5.020928 |
| H | 5.397842 | 0.056397 | 3.193159 |
| H | 2.759747 | 0.548999 | -0.555023 |

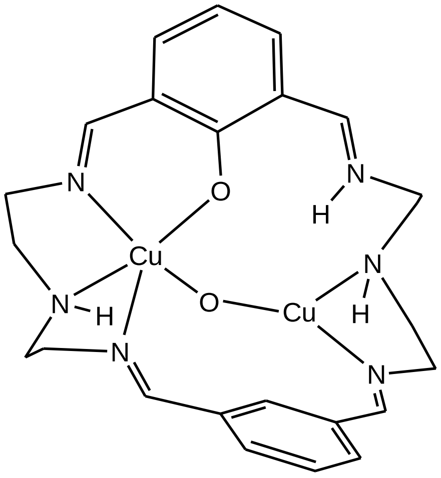

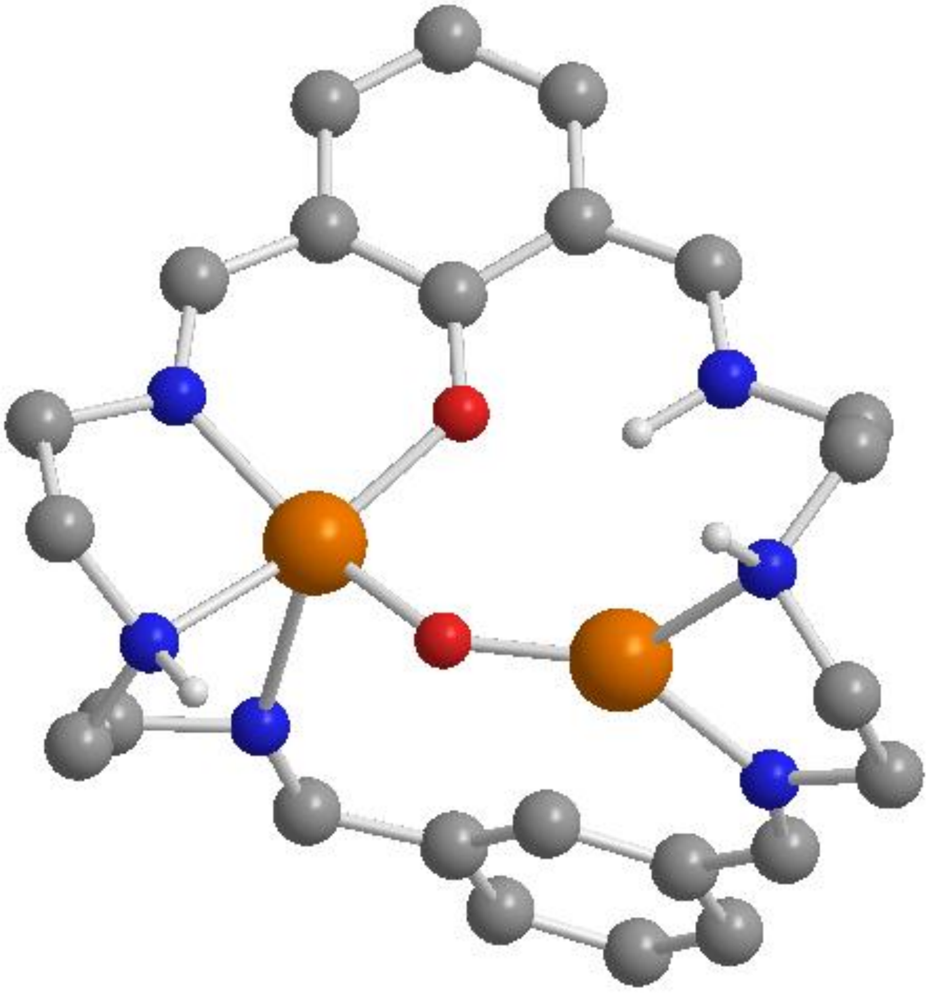

# TS-hg

| | | | |
|---|---|---|---|
| H | -5.724692 | -1.785997 | -0.772417 |
| H | -4.185736 | -2.168445 | -2.676102 |
| C | -4.652716 | -1.809225 | -0.605202 |
| H | -4.831294 | -1.597175 | 1.530206 |
| C | -3.785220 | -2.012045 | -1.677618 |
| C | -4.146462 | -1.691528 | 0.691330 |
| C | -2.393146 | -2.038371 | -1.462974 |
| C | -2.759820 | -1.727800 | 0.918965 |
| H | -1.932789 | -2.858143 | -3.432655 |
| C | -1.892381 | -1.878166 | -0.165374 |
| H | -2.865840 | -2.216728 | 3.052618 |
| C | -1.488720 | -2.360294 | -2.563644 |
| C | -2.196897 | -1.815390 | 2.277713 |
| H | -0.829781 | -1.961206 | 0.025379 |
| N | -0.219354 | -2.133843 | -2.558899 |
| N | -0.965001 | -1.572780 | 2.525423 |
| H | -1.194313 | -2.175699 | 4.571682 |
| H | 0.012065 | -3.123676 | -4.440974 |
| C | -0.427949 | -2.066450 | 3.790583 |
| C | 0.608170 | -2.626142 | -3.667099 |
| H | -0.027764 | -3.071118 | 3.591580 |
| H | 1.304657 | -3.369216 | -3.258122 |
| C | -0.798500 | 2.238524 | -2.113682 |
| H | -1.644793 | 1.666553 | -2.495804 |
| C | 0.767350 | 2.873219 | 2.581567 |
| H | 1.054017 | 3.653773 | 3.295252 |
| O | -0.131988 | 1.181256 | 0.332464 |
| C | -0.307036 | 2.437280 | 0.339389 |
| C | -0.715700 | 3.106432 | -0.893619 |
| C | -0.030403 | 3.315798 | 1.457392 |
| H | -1.721758 | 4.854830 | -1.738408 |
| C | -1.262776 | 4.436034 | -0.846870 |
| O | 1.457295 | -1.076411 | 0.268510 |
| C | -0.469147 | 4.637996 | 1.411715 |
| H | -0.289474 | 5.272364 | 2.277190 |
| Cu | 0.832554 | 0.048381 | 1.632166 |
| Cu | 0.973869 | -1.106283 | -1.398536 |
| C | -1.128575 | 5.192015 | 0.294043 |
| H | -1.485002 | 6.215371 | 0.326447 |
| N | 0.371758 | 2.296354 | -2.635722 |
| N | 1.215460 | 1.674888 | 2.741844 |
| H | 0.677683 | -0.838014 | -4.855169 |
| H | 0.277823 | -0.219315 | 4.627212 |
| C | 1.369358 | -1.439408 | -4.261179 |
| C | 0.695724 | -1.174622 | 4.298736 |
| N | 1.679896 | -0.896973 | 3.224086 |
| N | 1.911984 | -0.578852 | -3.159622 |
| H | 2.170779 | -1.775291 | -4.928247 |
| H | 1.185606 | -1.639206 | 5.164421 |
| H | 2.084242 | -1.769594 | 2.881129 |
| H | 2.845309 | -0.933607 | -2.936500 |
| H | 0.060017 | 1.062391 | -4.311765 |
| H | 1.620207 | 1.483303 | 4.807348 |
| C | 0.861447 | 1.651481 | -3.850417 |
| C | 2.758540 | 0.027030 | 3.648296 |
| C | 2.153537 | 1.419473 | 3.852150 |
| C | 2.138021 | 0.827479 | -3.612952 |
| H | 3.502190 | 0.049686 | 2.845897 |
| H | 2.754236 | 1.327183 | -2.860372 |
| H | 3.255035 | -0.314591 | 4.564758 |
| H | 2.707520 | 0.812253 | -4.550434 |
| H | 1.115411 | 2.460054 | -4.544712 |
| H | 2.948431 | 2.172848 | 3.872041 |
| H | 0.559534 | 3.166012 | -1.536639 |

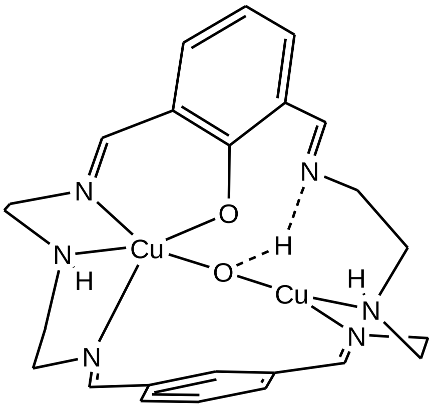


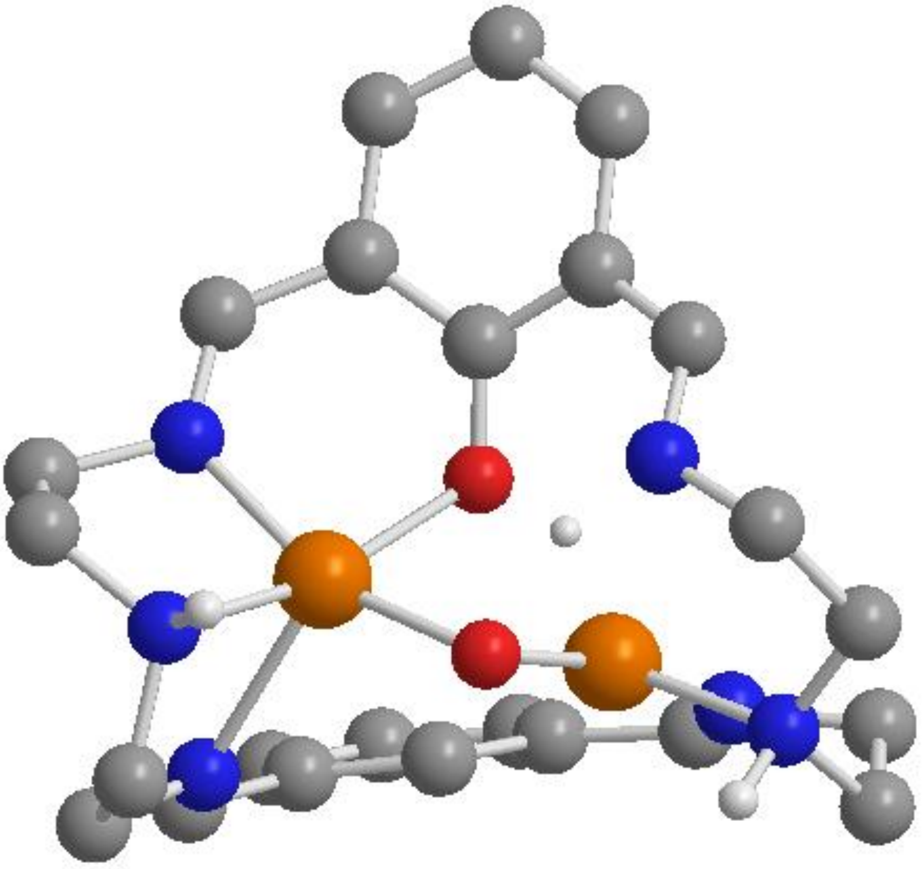

## TS-ej

| | | | |
|---|---|---|---|
| H | -5.551275 | -0.460243 | 0.717526 |
| H | -4.658172 | -0.542004 | -1.587824 |
| C | -4.504082 | -0.695787 | 0.556053 |
| H | -4.123781 | -1.070164 | 2.643990 |
| C | -3.995318 | -0.727824 | -0.746609 |
| C | -3.694955 | -1.026621 | 1.646334 |
| C | -2.643027 | -1.039072 | -0.963356 |
| C | -2.336930 | -1.323358 | 1.438853 |
| H | -2.755367 | -1.763582 | -3.036136 |
| C | -1.823835 | -1.314314 | 0.137011 |
| H | -1.839255 | -2.595472 | 3.176950 |
| C | -2.069973 | -1.346783 | -2.285099 |
| C | -1.432281 | -1.869493 | 2.463334 |
| H | -0.802403 | -1.635975 | -0.035563 |
| N | -0.805692 | -1.300976 | -2.500101 |
| N | -0.171471 | -1.624868 | 2.442313 |
| H | 0.232880 | -3.185063 | 3.834240 |
| H | -1.025050 | -2.230851 | -4.419749 |
| C | 0.761439 | -2.482236 | 3.178019 |
| C | -0.280676 | -2.071639 | -3.626618 |
| H | 1.282851 | -3.065458 | 2.408777 |
| H | -0.021325 | -3.061861 | -3.226497 |
| C | 0.454057 | 2.562762 | -2.641827 |
| H | 0.441339 | 3.272690 | -3.475871 |
| C | -0.476648 | 2.412679 | 2.324696 |
| H | -0.852924 | 3.061805 | 3.122386 |
| O | 0.935907 | 1.345970 | -0.029180 |
| C | -0.022229 | 2.431294 | -0.162864 |
| C | -0.348989 | 2.919298 | -1.480692 |
| C | -0.816266 | 2.828412 | 0.969580 |
| H | -1.585366 | 4.289807 | -2.582718 |
| C | -1.367725 | 3.873088 | -1.602972 |
| O | 1.307569 | -1.078084 | -0.045840 |
| C | -1.824719 | 3.778937 | 0.772828 |
| H | -2.394208 | 4.127940 | 1.630097 |
| Cu | 0.787898 | -0.139395 | 1.429105 |
| Cu | 1.028305 | -0.099105 | - 1.548782 |
| C | -2.111256 | 4.286987 | -0.496726 |
| H | -2.901472 | 5.019912 | -0.619698 |
| N | 1.190516 | 1.504716 | -2.706272 |
| N | 0.265163 | 1.402012 | 2.625852 |
| H | 0.670924 | -0.530684 | -4.773930 |
| H | 1.313894 | -1.322125 | 4.914298 |
| C | 0.965085 | -1.426218 | -4.222831 |
| C | 1.776793 | -1.663150 | 3.982671 |
| N | 2.251880 | -0.481282 | 3.219238 |
| N | 1.931301 | -1.016754 | -3.167881 |
| H | 1.439309 | -2.107710 | -4.939275 |
| H | 2.613034 | -2.309744 | 4.268398 |
| H | 3.207934 | -0.631882 | 2.907450 |
| H | 2.448888 | -1.832310 | -2.838187 |
| H | 1.572622 | 1.356505 | -4.786515 |
| H | 0.077994 | 0.504345 | 4.507343 |
| C | 2.122081 | 1.338771 | -3.838237 |
| C | 2.169430 | 0.773426 | 3.990885 |
| C | 0.712018 | 1.251323 | 4.015110 |
| C | 2.883103 | 0.021544 | -3.639913 |
| H | 2.792365 | 1.525024 | 3.495505 |
| H | 3.647880 | 0.141315 | -2.866750 |
| H | 2.534460 | 0.662343 | 5.021280 |
| H | 3.384817 | -0.272189 | -4.570001 |
| H | 2.830994 | 2.173402 | -3.861480 |
| H | 0.618092 | 2.190090 | 4.572150 |
| H | 1.164001 | 2.680092 | 0.060763 |

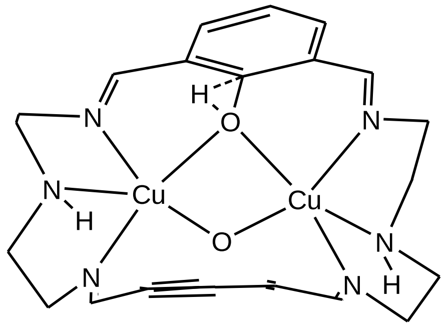


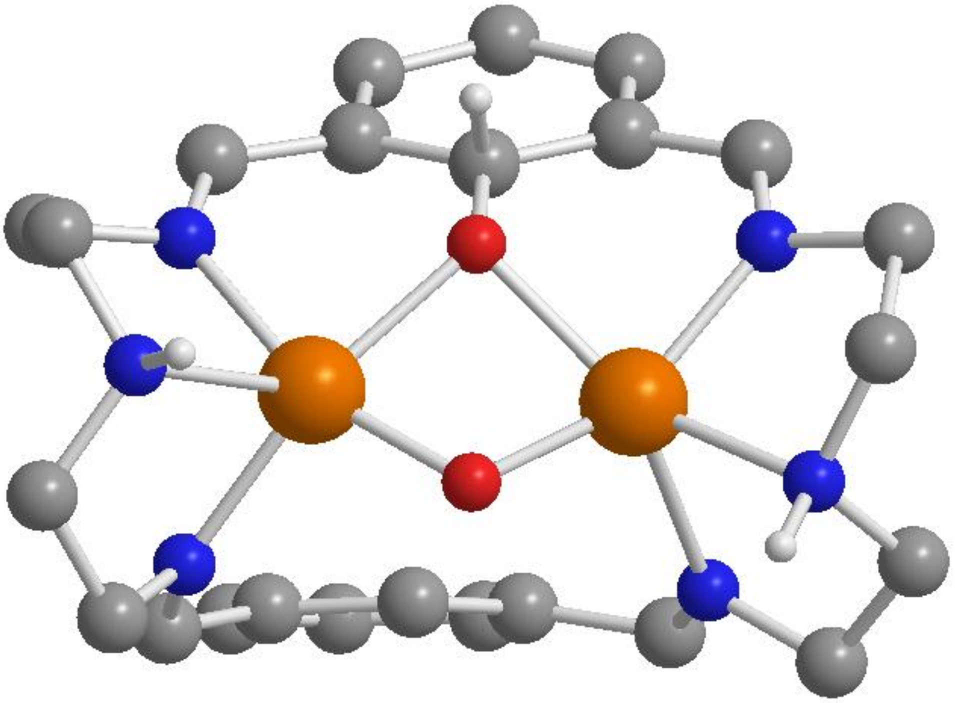

**j**

| | | | |
|---|---|---|---|
| H | -5.603742 | -3.246205 | -0.258422 |
| H | -4.252202 | -3.104131 | -2.328466 |
| C | -4.601555 | -2.833672 | -0.226204 |
| H | -4.614219 | -2.540192 | 1.906173 |
| C | -3.843197 | -2.741038 | -1.389348 |
| C | -4.046050 | -2.429150 | 0.986258 |
| C | -2.542348 | -2.196859 | -1.380181 |
| C | -2.748943 | -1.891936 | 1.047508 |
| H | -2.171112 | -2.886168 | -3.386078 |
| C | -2.020593 | -1.741972 | -0.153440 |
| H | -2.747930 | -1.748607 | 3.224910 |
| C | -1.741899 | -2.266695 | -2.591596 |
| C | -2.138151 | -1.565427 | 2.331954 |
| H | -0.514217 | -1.050671 | 0.862618 |
| N | -0.581304 | -1.733853 | -2.783608 |
| N | -0.933589 | -1.118081 | 2.431211 |
| H | -0.312799 | 0.211285 | 3.907662 |
| H | -0.191637 | -1.398919 | -4.832809 |
| C | -0.373153 | -0.871994 | 3.755770 |
| C | 0.133064 | -2.071736 | -4.031231 |
| H | -1.027347 | -1.273585 | 4.542143 |
| H | -0.092863 | -3.094263 | -4.352056 |
| C | -0.146883 | 2.814031 | -2.300796 |
| H | -0.277802 | 3.531869 | -3.121416 |
| C | 1.016744 | 2.904114 | 2.560056 |
| H | 1.039471 | 3.573874 | 3.426455 |
| O | -0.780009 | -1.205354 | -0.116161 |
| C | 0.377458 | 2.822339 | 0.132719 |
| C | -0.310584 | 3.373247 | -0.950410 |
| C | 0.303965 | 3.423269 | 1.398190 |
| H | -1.590431 | 4.987941 | -1.612885 |
| C | -1.073043 | 4.542399 | -0.767147 |
| O | 1.707671 | -0.165993 | -0.225624 |
| C | -0.467087 | 4.591405 | 1.566276 |
| H | -0.513531 | 5.073384 | 2.539410 |
| Cu | 1.643081 | 0.189982 | 1.473900 |
| Cu | 0.498782 | -0.535441 | -1.584237 |
| C | -1.161412 | 5.139319 | 0.489750 |
| H | -1.751222 | 6.039959 | 0.624644 |
| N | 0.179522 | 1.601695 | -2.558322 |
| N | 1.590874 | 1.751561 | 2.667955 |
| H | 2.197057 | -2.007341 | -4.707909 |
| H | 1.287777 | -1.494485 | 4.979761 |
| C | 1.632891 | -1.921485 | -3.771357 |
| C | 1.010567 | -1.515811 | 3.918960 |
| N | 2.102854 | -0.882765 | 3.111434 |
| N | 1.891374 | -0.627993 | -3.092373 |
| H | 1.970543 | -2.715773 | -3.098076 |
| H | 0.945907 | -2.565325 | 3.618674 |
| H | 2.717730 | -1.636548 | 2.805695 |
| H | 2.784540 | -0.672152 | -2.604743 |
| H | -0.274855 | 0.639085 | -4.357272 |
| H | 1.473439 | 1.407571 | 4.754713 |
| C | 0.509550 | 1.283643 | -3.947330 |
| C | 2.955092 | 0.097823 | 3.850080 |
| C | 2.235221 | 1.440607 | 3.966417 |
| C | 1.872226 | 0.573313 | -3.972687 |
| H | 3.874456 | 0.219278 | 3.267781 |
| H | 2.634544 | 1.264698 | -3.603941 |
| H | 3.233572 | -0.280476 | 4.840445 |
| H | 2.135923 | 0.300879 | -5.001820 |
| H | 0.563028 | 2.178543 | -4.581433 |
| H | 2.955605 | 2.219143 | 4.238844 |
| H | 1.020724 | 1.967150 | -0.035116 |

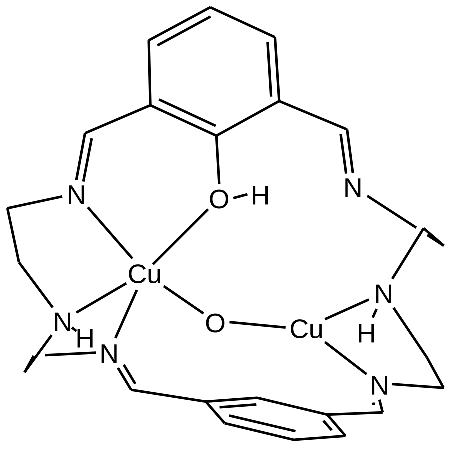


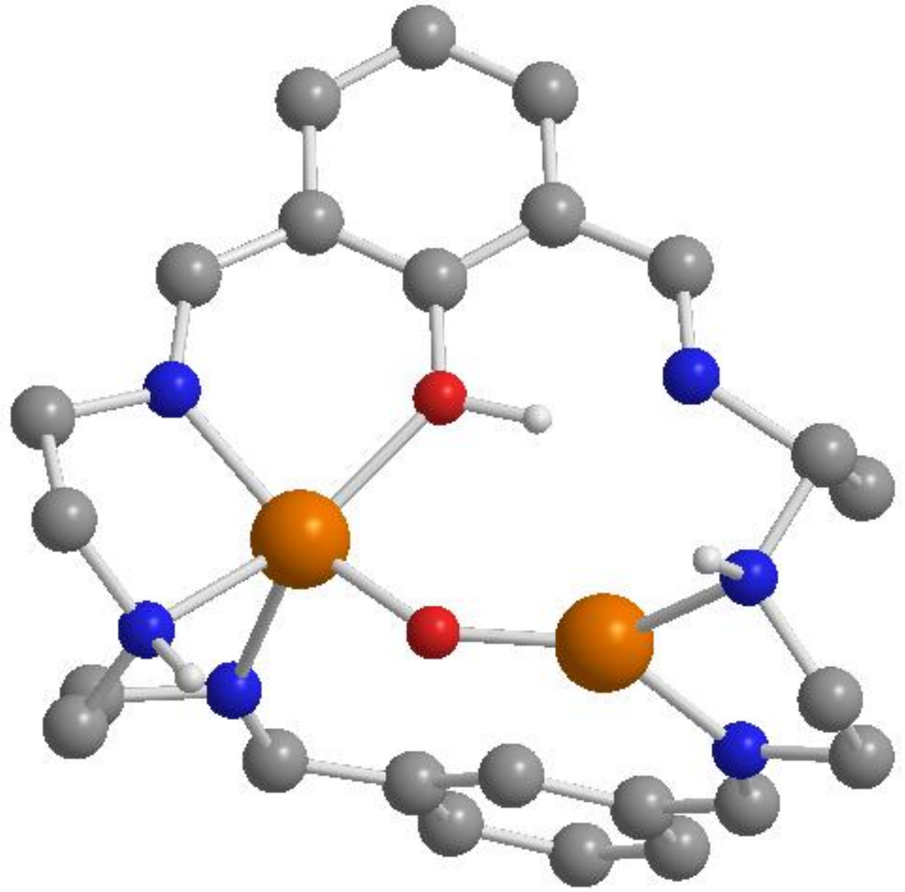

## TS-jh

| | | | |
|---|---|---|---|
| H | -5.269504 | -0.415340 | -0.091381 |
| H | -4.194314 | -1.040492 | -2.231054 |
| C | -4.225183 | -0.706047 | -0.109559 |
| H | -3.987867 | -0.619345 | 2.028500 |
| C | -3.610635 | -1.043159 | -1.314004 |
| C | -3.500932 | -0.807598 | 1.075039 |
| C | -2.251728 | -1.409414 | -1.378990 |
| C | -2.145623 | -1.181002 | 1.060022 |
| H | -2.462427 | -2.207253 | -3.375386 |
| C | -1.497809 | -1.420286 | -0.182000 |
| H | -1.979279 | -1.436277 | 3.228331 |
| C | -1.715940 | -1.941514 | -2.619070 |
| C | -1.430715 | -1.483557 | 2.282256 |
| H | 0.099803 | -1.879446 | 0.913810 |
| N | -0.471266 | -2.187114 | -2.855435 |
| N | -0.198858 | -1.885496 | 2.250932 |
| H | -0.234995 | -2.286002 | 4.319317 |
| H | -0.202891 | -2.255526 | -4.956923 |
| C | 0.428312 | -2.422652 | 3.455556 |
| C | -0.121127 | -2.920201 | -4.089482 |
| H | 0.546844 | -3.506207 | 3.315214 |
| H | -0.810510 | -3.755140 | -4.253381 |
| C | 1.792030 | 1.665877 | -2.780713 |
| H | 1.859276 | 2.327589 | -3.654739 |
| C | 3.504644 | 1.546821 | 1.895826 |
| H | 3.899233 | 2.190072 | 2.689782 |
| O | -0.196037 | -1.731497 | -0.178192 |
| C | 2.539669 | 1.599934 | -0.414622 |
| C | 1.999890 | 2.321312 | -1.480478 |
| C | 2.873955 | 2.246859 | 0.783282 |
| H | 1.384949 | 4.275639 | -2.177803 |
| C | 1.783071 | 3.705166 | -1.342622 |
| O | 2.526252 | -1.777855 | -0.576760 |
| C | 2.645580 | 3.631864 | 0.909063 |
| H | 2.918793 | 4.144021 | 1.828035 |
| Cu | 2.832392 | -1.230394 | 1.038580 |
| Cu | 1.115043 | -1.654461 | -1.769073 |
| C | 2.087810 | 4.352227 | -0.145408 |
| H | 1.916691 | 5.418899 | -0.044367 |
| N | 1.612966 | 0.407234 | -2.937233 |
| N | 3.635700 | 0.268420 | 2.024556 |
| H | 1.686009 | -3.823089 | -4.892231 |
| H | 1.688797 | -0.709018 | 3.832670 |
| C | 1.307339 | -3.446499 | -3.934106 |
| C | 1.780982 | -1.797952 | 3.797275 |
| N | 2.852778 | -2.148290 | 2.826961 |
| N | 2.174037 | -2.378470 | -3.380875 |
| H | 1.317692 | -4.274164 | -3.217697 |
| H | 2.068773 | -2.143715 | 4.799277 |
| H | 2.852635 | -3.162847 | 2.706625 |
| H | 3.003515 | -2.795468 | -2.961880 |
| H | 0.643548 | -0.378056 | -4.614115 |
| H | 3.910629 | 0.247992 | 4.128919 |
| C | 1.657248 | -0.103835 | -4.306541 |
| C | 4.213383 | -1.744136 | 3.290476 |
| C | 4.338559 | -0.218654 | 3.234168 |
| C | 2.590204 | -1.323971 | -4.344580 |
| H | 4.933443 | -2.210614 | 2.610641 |
| H | 3.597324 | -1.002970 | -4.065412 |
| H | 4.419439 | -2.112613 | 4.302244 |
| H | 2.637909 | -1.728954 | -5.363108 |
| H | 2.024267 | 0.645067 | -5.020928 |
| H | 5.397842 | 0.056397 | 3.193159 |
| H | 2.759747 | 0.548999 | -0.555023 |

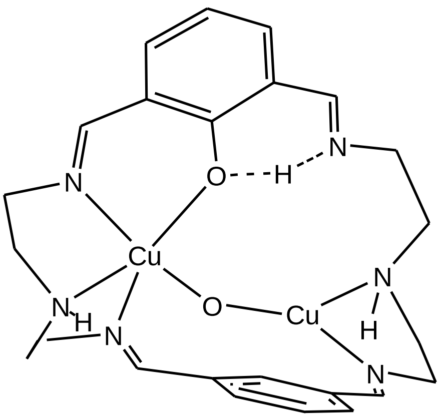


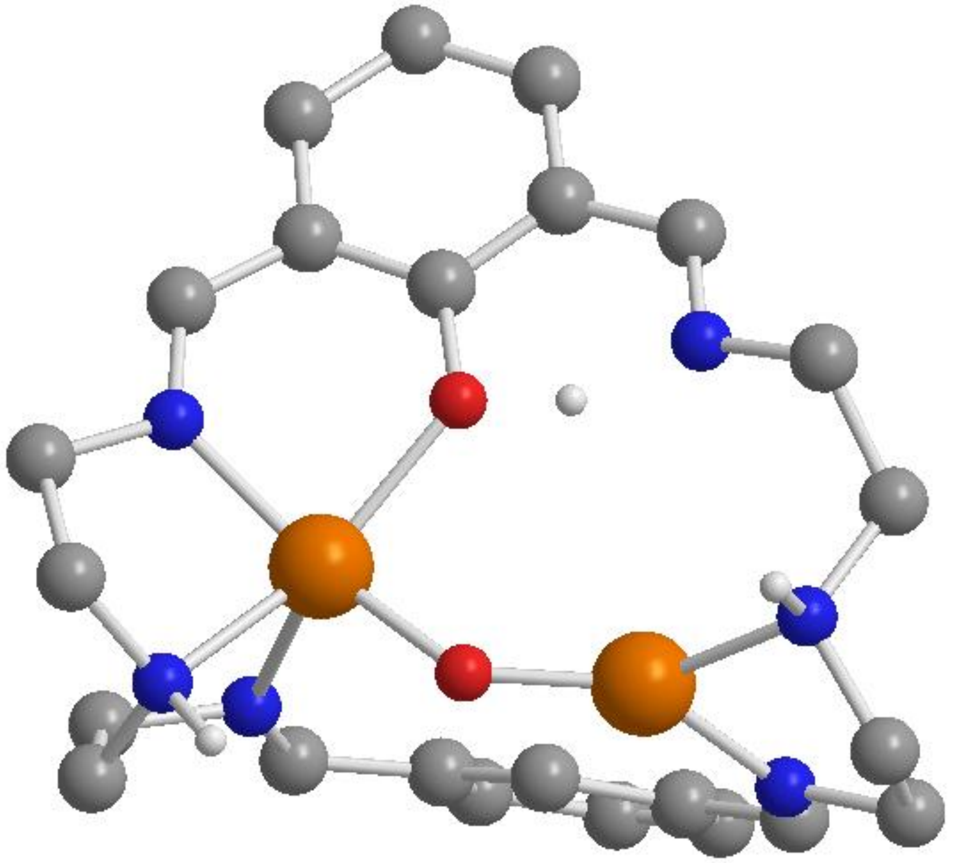

## TS-jg

| | | | |
|---|---|---|---|
| H | -4.578476 | 0.075687 | 1.071886 |
| H | -3.969106 | 0.001257 | -1.332296 |
| C | -3.646993 | -0.383621 | 0.759817 |
| H | -3.082988 | -0.964145 | 2.750966 |
| C | -3.301266 | -0.425447 | -0.588738 |
| C | -2.800318 | -0.963546 | 1.701473 |
| C | -2.096378 | -1.014692 | -1.008980 |
| C | -1.586002 | -1.566285 | 1.325142 |
| H | -2.645586 | -1.067542 | -3.114962 |
| C | -1.246699 | -1.592623 | -0.042439 |
| H | -1.342888 | -2.684255 | 3.176840 |
| C | -1.785991 | -1.097987 | -2.436514 |
| C | -0.778079 | -2.259679 | 2.336154 |
| H | 0.617832 | -2.261562 | 0.122025 |
| N | -0.604241 | -1.233961 | -2.927836 |
| N | 0.495082 | -2.420760 | 2.266210 |
| H | 0.402921 | -3.690868 | 3.985033 |
| H | 0.079205 | -0.700487 | -4.826035 |
| C | 1.127320 | -3.289354 | 3.263600 |
| C | -0.438719 | -1.537665 | -4.349223 |
| H | 1.561533 | -4.142638 | 2.725803 |
| H | -1.404145 | -1.661080 | -4.853777 |
| C | 2.435982 | 1.122695 | -3.087273 |
| H | 2.877064 | 1.622283 | -3.958035 |
| C | 2.497282 | 1.867555 | 1.813461 |
| H | 2.660710 | 2.669711 | 2.540924 |
| O | -0.171545 | -2.294030 | -0.496452 |
| C | 2.310549 | 1.476376 | -0.643400 |
| C | 2.146758 | 1.982283 | -1.935877 |
| C | 2.180873 | 2.322843 | 0.463757 |
| H | 1.747293 | 3.748116 | -3.126796 |
| C | 1.842351 | 3.344148 | -2.122224 |
| O | 2.162485 | -1.507971 | -0.331950 |
| C | 1.857331 | 3.679908 | 0.267431 |
| H | 1.772757 | 4.346231 | 1.122085 |
| Cu | 2.332655 | -1.164367 | 1.406339 |
| Cu | 1.121375 | -1.361294 | -1.884645 |
| C | 1.669962 | 4.180862 | -1.020159 |
| H | 1.425402 | 5.227899 | -1.165505 |
| N | 2.254182 | -0.149446 | -3.122754 |
| N | 2.642962 | 0.649301 | 2.214427 |
| H | 0.688424 | -3.001797 | -5.501342 |
| H | 1.763073 | -1.729131 | 4.598358 |
| C | 0.379013 | -2.844222 | -4.459389 |
| C | 2.219691 | -2.534132 | 4.016531 |
| N | 3.195133 | -1.921264 | 3.069910 |
| N | 1.535386 | -2.848533 | -3.529727 |
| H | -0.269503 | -3.678625 | -4.177201 |
| H | 2.732754 | -3.200945 | 4.719052 |
| H | 3.854403 | -2.646752 | 2.782965 |
| H | 1.676123 | -3.797057 | -3.190469 |
| H | 2.155077 | -0.614623 | -5.183825 |
| H | 2.223311 | 0.365562 | 4.257390 |
| C | 2.779915 | -0.855335 | -4.315936 |
| C | 3.963292 | -0.794015 | 3.671511 |
| C | 3.102150 | 0.465024 | 3.608425 |
| C | 2.815317 | -2.364447 | -4.082315 |
| H | 4.871584 | -0.653751 | 3.077987 |
| H | 3.591513 | -2.602953 | -3.348382 |
| H | 4.259848 | -1.015219 | 4.703439 |
| H | 3.076731 | -2.863609 | -5.025994 |
| H | 3.792445 | -0.505441 | -4.542328 |
| H | 3.674896 | 1.329772 | 3.959251 |
| H | 2.608079 | 0.442513 | -0.508395 |

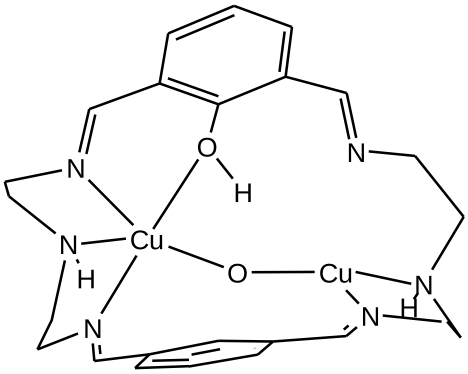


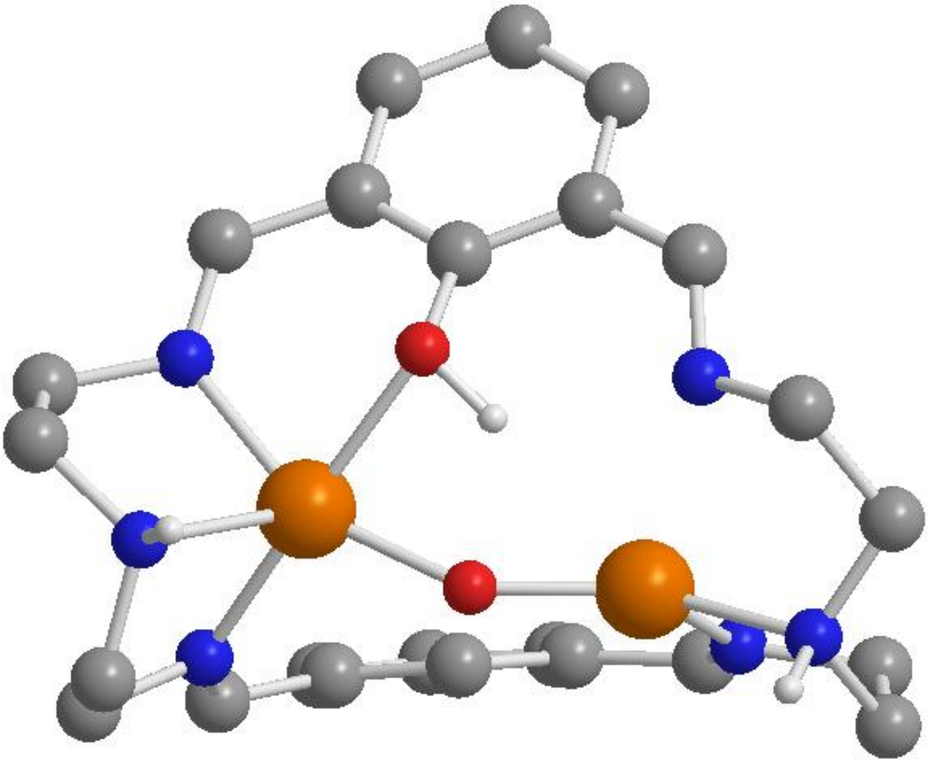

## d-withoutH

| | | | |
|---|---|---|---|
| H | -5.744274 | -1.762874 | 0.002653 |
| H | -4.516680 | -1.887530 | -2.145159 |
| C | -4.658945 | -1.792337 | 0.003544 |
| H | -4.519762 | -1.872622 | 2.153059 |
| C | -3.965954 | -1.845788 | -1.208629 |
| C | -3.967714 | -1.837374 | 1.217043 |
| C | -2.562243 | -1.875284 | -1.212508 |
| C | -2.563994 | -1.866687 | 1.223097 |
| H | -2.214733 | -2.785203 | -3.190923 |
| C | -1.874634 | -1.855594 | 0.005740 |
| H | -2.218120 | -2.764206 | 3.207498 |
| C | -1.752012 | -2.162001 | -2.410388 |
| C | -1.755252 | -2.144874 | 2.423991 |
| H | -0.790985 | -1.926469 | 0.006762 |
| N | -0.515463 | -1.838465 | -2.461148 |
| N | -0.519290 | -1.819072 | 2.474784 |
| H | -0.197713 | -2.993506 | 4.235892 |
| H | -0.193099 | -3.022583 | -4.215685 |
| C | 0.352949 | -2.482259 | 3.430776 |
| C | 0.357057 | -2.508488 | -3.412015 |
| H | 0.889138 | -3.253974 | 2.860814 |
| H | 0.890523 | -3.278571 | -2.837277 |
| C | -0.436468 | 2.529528 | -2.465429 |
| H | -0.520331 | 3.286797 | -3.255088 |
| C | -0.452155 | 2.547481 | 2.442472 |
| H | -0.541601 | 3.310395 | 3.226071 |
| O | -0.241565 | 1.030042 | -0.005097 |
| C | -0.833766 | 2.180233 | -0.011370 |
| C | -1.130513 | 2.860561 | -1.240181 |
| C | -1.138470 | 2.869476 | 1.210467 |
| H | -2.164546 | 4.504573 | -2.161389 |
| C | -1.929227 | 4.017180 | -1.217627 |
| O | 1.450529 | -0.895588 | 0.006656 |
| C | -1.937040 | 4.025828 | 1.174358 |
| H | -2.178500 | 4.520057 | 2.113001 |
| Cu | 0.677616 | -0.045418 | 1.446538 |
| Cu | 0.684320 | -0.056585 | -1.443371 |
| C | -2.389713 | 4.568916 | -0.025114 |
| H | -3.017607 | 5.452940 | -0.030363 |
| N | 0.342833 | 1.516561 | -2.659346 |
| N | 0.326598 | 1.536528 | 2.648817 |
| H | 0.861301 | -0.921224 | -4.762495 |
| H | 0.851764 | -0.888132 | 4.775485 |
| C | 1.372564 | -1.544641 | -4.025329 |
| C | 1.365291 | -1.513177 | 4.041247 |
| N | 1.961120 | -0.618381 | 3.023188 |
| N | 1.969627 | -0.647214 | -3.010339 |
| H | 2.145618 | -2.108061 | -4.564583 |
| H | 2.139185 | -2.072438 | 4.583576 |
| H | 2.731715 | -1.080420 | 2.542928 |
| H | 2.735844 | -1.110702 | -2.524451 |
| H | 0.603986 | 1.100167 | -4.708586 |
| H | 0.576449 | 1.130764 | 4.701367 |
| C | 1.176123 | 1.508233 | -3.865966 |
| C | 2.393703 | 0.677744 | 3.587526 |
| C | 1.152032 | 1.538097 | 3.860735 |
| C | 2.411778 | 0.643033 | -3.580836 |
| H | 3.022872 | 1.170988 | 2.840240 |
| H | 3.039864 | 1.137648 | -2.833558 |
| H | 2.984781 | 0.558341 | 4.506342 |
| H | 3.006786 | 0.514637 | -4.495886 |
| H | 1.490399 | 2.520224 | -4.148935 |
| H | 1.459281 | 2.552945 | 4.141179 |

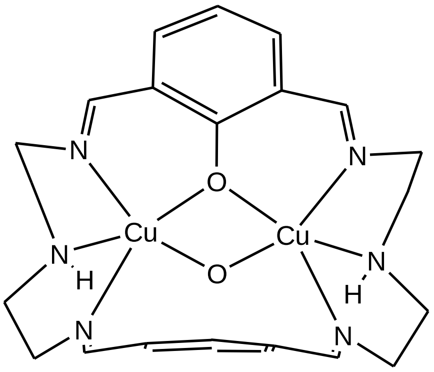


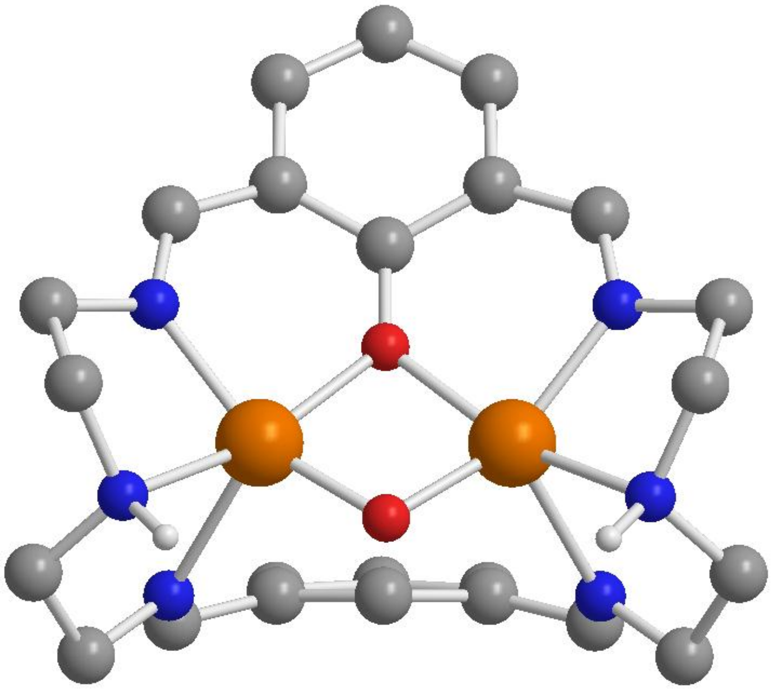

**m**

| | | | |
|---|---|---|---|
| H | -0.401785 | 4.130238 | 4.308042 |
| H | 1.846631 | 3.616290 | 3.409386 |
| C | -0.301169 | 3.731848 | 3.303775 |
| H | -2.416091 | 3.811275 | 2.903870 |
| C | 0.966321 | 3.437027 | 2.797816 |
| C | -1.436876 | 3.547348 | 2.513555 |
| C | 1.104081 | 2.919404 | 1.497819 |
| C | -1.310592 | 3.022886 | 1.214687 |
| H | 3.215382 | 3.376939 | 1.229059 |
| C | -0.039214 | 2.733699 | 0.716574 |
| H | -3.208857 | 3.673868 | 0.334692 |
| C | 2.413596 | 2.713318 | 0.882404 |
| C | -2.444948 | 2.887744 | 0.298441 |
| H | 0.066928 | 2.414270 | -0.310468 |
| N | 2.654032 | 1.893841 | -0.086926 |
| N | -2.535582 | 1.956128 | -0.590461 |
| H | -4.218300 | 2.986880 | -1.419079 |
| H | 4.657543 | 2.650142 | -0.197089 |
| C | -3.533155 | 2.163899 | -1.657089 |
| C | 3.911501 | 2.144375 | -0.822597 |
| H | -2.976669 | 2.455278 | -2.558327 |
| H | 3.666451 | 2.826757 | -1.648436 |
| C | 2.479047 | -2.766872 | 0.645853 |
| H | 3.289279 | -3.479924 | 0.842374 |
| C | -2.438354 | -2.549602 | 1.183818 |
| H | -3.248400 | -3.162674 | 1.595603 |
| O | 0.040229 | 0.051505 | 1.180823 |
| C | 0.030781 | -2.630731 | 0.969634 |
| C | 1.295875 | -2.915943 | 1.487655 |
| C | -1.115387 | -2.808243 | 1.747100 |
| H | 2.385506 | -3.695274 | 3.189634 |
| C | 1.408074 | -3.437885 | 2.790249 |
| O | 0.013892 | 0.015539 | -0.158050 |
| C | -0.990896 | -3.327137 | 3.048720 |
| H | -1.879112 | -3.498467 | 3.651173 |
| Cu | -1.910367 | -0.002495 | -0.625406 |
| Cu | 1.907510 | 0.019872 | -0.665500 |
| C | 0.267925 | -3.627497 | 3.569747 |
| H | 0.357642 | -4.027426 | 4.574341 |
| N | 2.622222 | -1.911957 | -0.311729 |
| N | -2.712566 | -1.711332 | 0.242973 |
| H | 4.865174 | 0.235993 | -0.563760 |
| H | -4.971222 | 0.684034 | -1.065719 |
| C | 4.485887 | 0.851557 | -1.383499 |
| C | -4.320582 | 0.887263 | -1.920792 |
| N | -3.388655 | -0.261208 | -2.071192 |
| N | 3.402796 | 0.099447 | -2.064074 |
| H | 5.324827 | 1.059941 | -2.058897 |
| H | -4.962789 | 1.002851 | -2.801985 |
| H | -3.035317 | -0.263326 | -3.028096 |
| H | 3.149394 | 0.600279 | -2.916716 |
| H | 4.727699 | -1.892473 | -0.564447 |
| H | -4.700199 | -1.018075 | 0.195853 |
| C | 3.830541 | -2.137098 | -1.146097 |
| C | -4.042057 | -1.567071 | -1.799476 |
| C | -4.088574 | -1.789704 | -0.288425 |
| C | 3.757541 | -1.294630 | -2.414948 |
| H | -3.439432 | -2.351068 | -2.267639 |
| H | 2.968979 | -1.675820 | -3.071447 |
| H | -5.051479 | -1.612718 | -2.227139 |
| H | 4.708655 | -1.351727 | -2.959686 |
| H | 3.908656 | -3.192024 | -1.427512 |
| H | -4.551983 | -2.758030 | -0.070395 |
| H | -0.064293 | -2.317610 | -0.060030 |

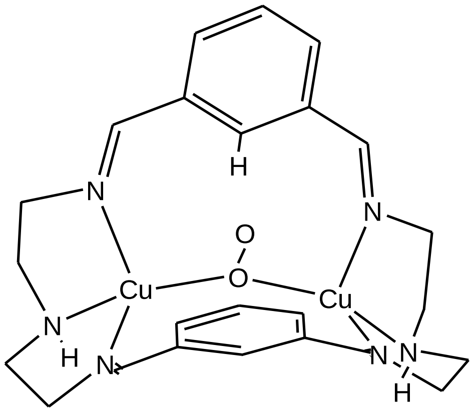


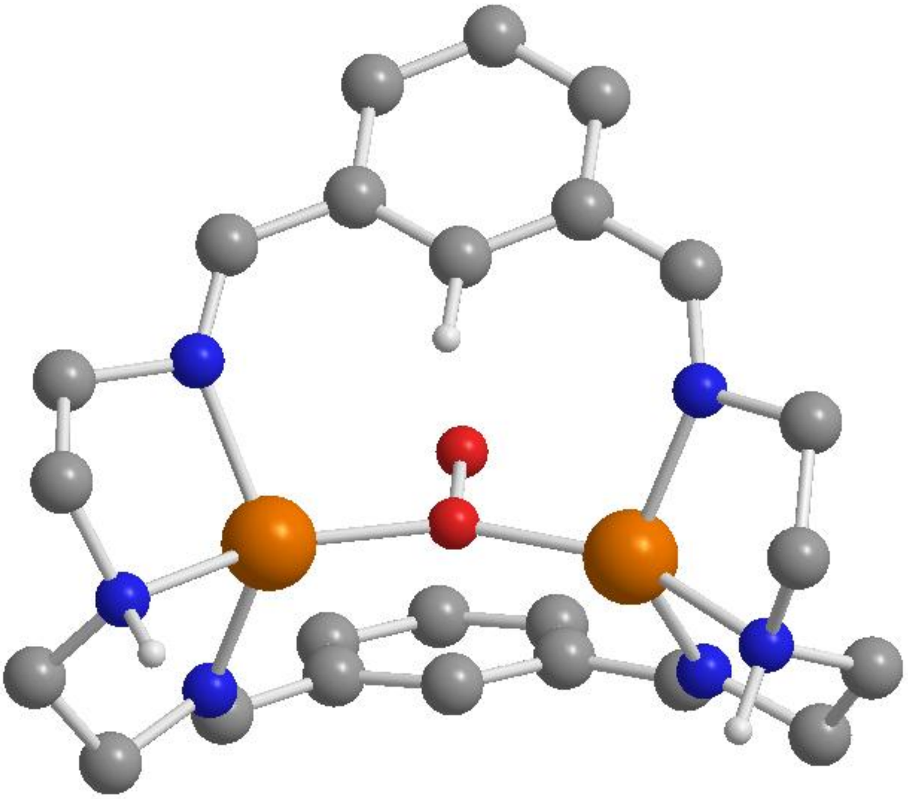

## n

| | | | |
|---|---|---|---|
| H | -0.585621 | 3.901544 | 4.320608 |
| H | 1.696899 | 3.366896 | 3.523179 |
| C | -0.440510 | 3.521233 | 3.314738 |
| H | -2.525120 | 3.676919 | 2.796345 |
| C | 0.844494 | 3.210930 | 2.867105 |
| C | -1.533937 | 3.386891 | 2.457936 |
| C | 1.040628 | 2.720836 | 1.563278 |
| C | -1.350448 | 2.884649 | 1.156863 |
| H | 3.170975 | 3.159570 | 1.473026 |
| C | -0.063641 | 2.561075 | 0.721240 |
| H | -3.183616 | 3.623469 | 0.198661 |
| C | 2.383058 | 2.552374 | 1.009882 |
| C | -2.439042 | 2.818349 | 0.177326 |
| H | 0.088944 | 2.251801 | -0.301347 |
| N | 2.665755 | 1.834388 | -0.024108 |
| N | -2.504321 | 1.909612 | -0.733772 |
| H | -4.116099 | 2.977332 | -1.648596 |
| H | 4.697257 | 2.515715 | 0.055106 |
| C | -3.466801 | 2.111467 | -1.828760 |
| C | 3.973058 | 2.097813 | -0.655501 |
| H | -2.881525 | 2.323329 | -2.733563 |
| H | 3.806152 | 2.857406 | -1.431972 |
| C | 2.534957 | -2.542359 | 0.625304 |
| H | 3.391320 | -3.163602 | 0.914814 |
| C | -2.438137 | -2.265531 | 1.389254 |
| H | -3.231088 | -2.800965 | 1.922833 |
| O | 0.126871 | -0.601017 | 1.029094 |
| C | 0.068058 | -2.057627 | 1.032958 |
| C | 1.390022 | -2.621281 | 1.490924 |
| C | -1.102568 | -2.484659 | 1.880202 |
| H | 2.467308 | -3.666058 | 3.017865 |
| C | 1.497740 | -3.292915 | 2.697286 |
| O | 0.023317 | -0.128289 | -0.356281 |
| C | -0.915304 | -3.165422 | 3.067971 |
| H | -1.777399 | -3.443411 | 3.669209 |
| Cu | -1.889483 | -0.041624 | -0.698444 |
| Cu | 1.888681 | 0.035927 | -0.799782 |
| C | 0.372654 | -3.534981 | 3.508581 |
| H | 0.490892 | -4.053012 | 4.454278 |
| N | 2.593825 | -1.856121 | -0.477756 |
| N | -2.745175 | -1.558700 | 0.346563 |
| H | 4.841431 | 0.134058 | -0.510085 |
| H | -4.992107 | 0.744941 | -1.183326 |
| C | 4.532281 | 0.831505 | -1.293350 |
| C | -4.310585 | 0.856203 | -2.031456 |
| N | -3.436145 | -0.346164 | -2.079511 |
| N | 3.462522 | 0.182948 | -2.091036 |
| H | 5.415035 | 1.059678 | -1.903267 |
| H | -4.924993 | 0.943434 | -2.935234 |
| H | -3.109077 | -0.467805 | -3.037606 |
| H | 3.275217 | 0.760179 | -2.911990 |
| H | 4.693779 | -2.052675 | -0.754030 |
| H | -4.718569 | -0.831703 | 0.298099 |
| C | 3.765236 | -2.138317 | -1.333491 |
| C | -4.135178 | -1.586636 | -1.654323 |
| C | -4.136284 | -1.660406 | -0.125903 |
| C | 3.773444 | -1.195892 | -2.536260 |
| H | -3.584603 | -2.440024 | -2.061125 |
| H | 2.991612 | -1.487917 | -3.244035 |
| H | -5.161094 | -1.629029 | -2.041168 |
| H | 4.738536 | -1.252434 | -3.055255 |
| H | 3.712079 | -3.171178 | -1.695873 |
| H | -4.609644 | -2.592796 | 0.201917 |
| H | -0.110776 | -2.348329 | -0.007741 |

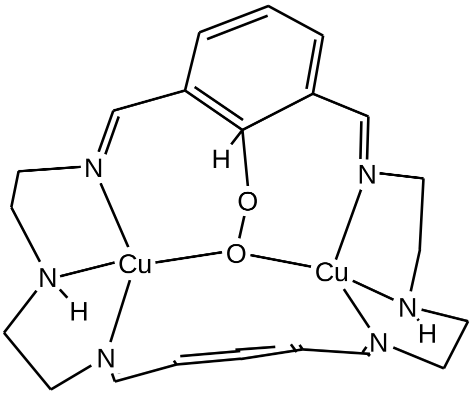


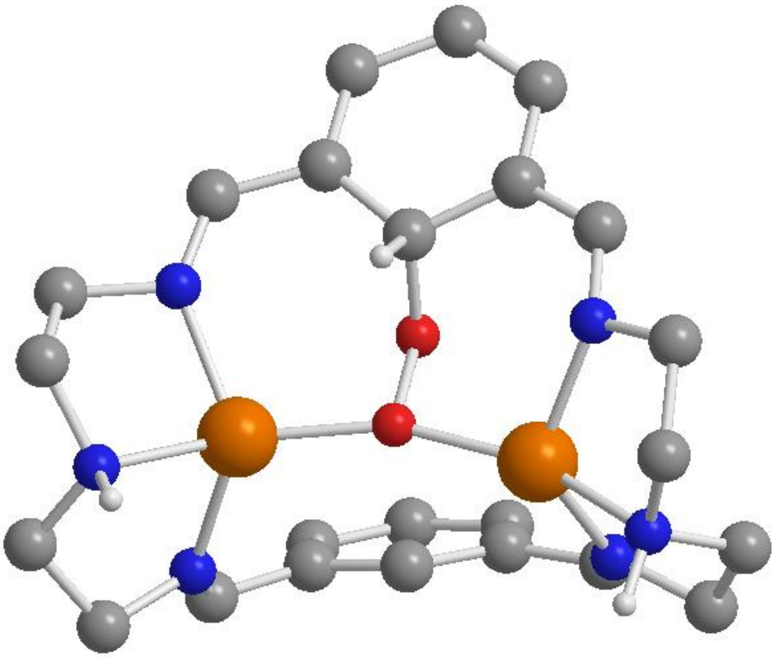

## mn

| | | | |
|---|---|---|---|
| H | 1.255413 | -3.820702 | 4.263833 |
| H | -1.124265 | -3.424765 | 3.703936 |
| C | 0.984735 | -3.478134 | 3.270518 |
| H | 3.015341 | -3.487693 | 2.553935 |
| C | -0.356729 | -3.252156 | 2.954088 |
| C | 1.975918 | -3.288056 | 2.307853 |
| C | -0.716186 | -2.816562 | 1.666958 |
| C | 1.626712 | -2.831430 | 1.022692 |
| H | -2.844957 | -3.215596 | 1.889850 |
| C | 0.282927 | -2.631047 | 0.708662 |
| H | 3.423578 | -3.387987 | -0.111657 |
| C | -2.117177 | -2.666409 | 1.279044 |
| C | 2.617630 | -2.648187 | -0.039675 |
| H | 0.004223 | -2.368211 | -0.300178 |
| N | -2.522668 | -2.010059 | 0.246399 |
| N | 2.556700 | -1.699143 | -0.911707 |
| H | 4.157784 | -2.620843 | -1.987903 |
| H | -4.575710 | -2.447669 | 0.691885 |
| C | 3.476360 | -1.765050 | -2.062585 |
| C | -3.918332 | -2.260060 | -0.166274 |
| H | 2.862579 | -1.910630 | -2.961039 |
| H | -3.920192 | -3.171293 | -0.780458 |
| C | -2.651892 | 2.365962 | 0.121988 |
| H | -3.621701 | 2.845618 | 0.305558 |
| C | 2.145790 | 2.637575 | 1.249159 |
| H | 2.876616 | 3.293548 | 1.735516 |
| O | -0.109863 | 0.348968 | 0.966350 |
| C | -0.262733 | 2.385043 | 0.767548 |
| C | -1.619150 | 2.660339 | 1.077724 |
| C | 0.764219 | 2.835889 | 1.637534 |
| H | -2.969321 | 3.503274 | 2.546548 |
| C | -1.930982 | 3.299618 | 2.297774 |
| O | -0.026157 | -0.030497 | -0.346235 |
| C | 0.424139 | 3.489165 | 2.822780 |
| H | 1.207140 | 3.843632 | 3.488505 |
| Cu | 1.818528 | 0.159477 | -0.781452 |
| Cu | -1.818428 | -0.260252 | -0.879621 |
| C | -0.922554 | 3.700370 | 3.165366 |
| H | -1.170513 | 4.202480 | 4.094252 |
| N | -2.517502 | 1.627036 | -0.949136 |
| N | 2.557441 | 1.791395 | 0.360362 |
| H | -4.645691 | -0.231478 | -0.354319 |
| H | 4.956940 | -0.398439 | -1.317631 |
| C | -4.455924 | -1.096847 | -0.995559 |
| C | 4.266929 | -0.465237 | -2.163668 |
| N | 3.339608 | 0.696739 | -2.089948 |
| N | -3.420610 | -0.717796 | -1.989169 |
| H | -5.401935 | -1.370044 | -1.478580 |
| H | 4.865734 | -0.442461 | -3.081551 |
| H | 2.986300 | 0.882534 | -3.028780 |
| H | -3.271658 | -1.509879 | -2.617007 |
| H | -4.598108 | 1.859779 | -1.358950 |
| H | 4.533474 | 1.068549 | 0.340691 |
| C | -3.651356 | 1.724067 | -1.899110 |
| C | 4.004594 | 1.930364 | -1.581780 |
| C | 3.964424 | 1.920871 | -0.053400 |
| C | -3.706587 | 0.492263 | -2.797781 |
| H | 3.446638 | 2.793170 | -1.956704 |
| H | -2.930525 | 0.548041 | -3.567615 |
| H | 5.035507 | 2.006088 | -1.948493 |
| H | -4.677997 | 0.427024 | -3.302916 |
| H | -3.511357 | 2.613399 | -2.523639 |
| H | 4.431093 | 2.834067 | 0.333863 |
| H | -0.008569 | 2.189878 | -0.267511 |

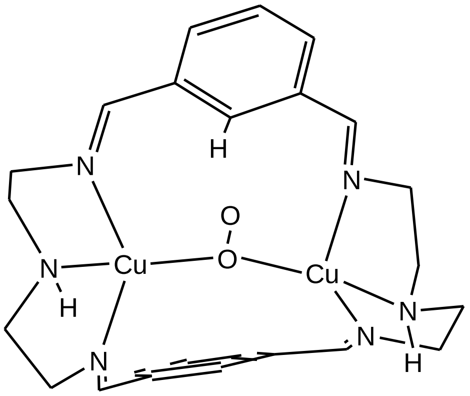


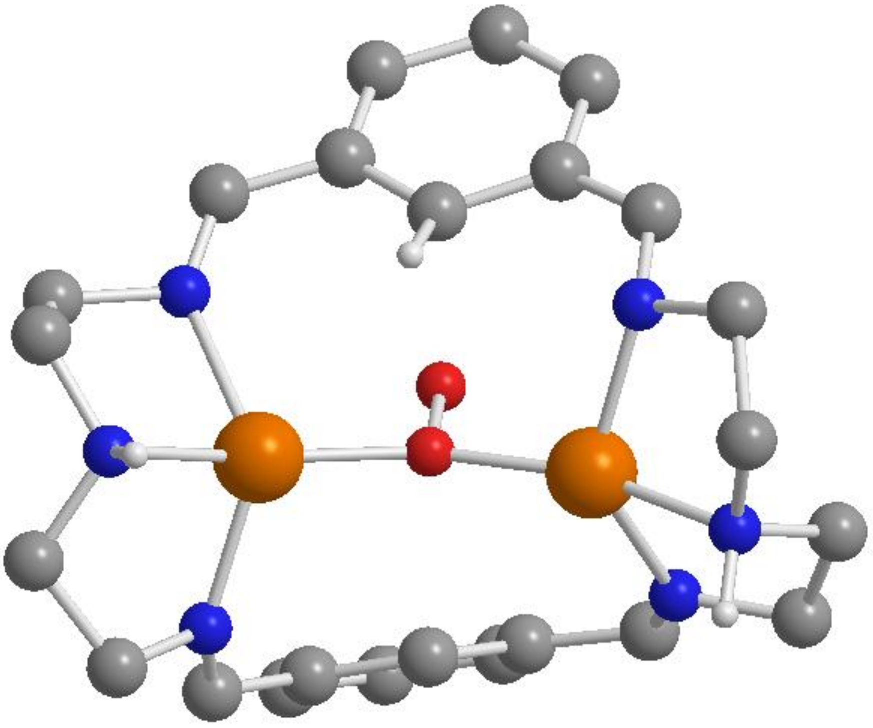

# nh

| | | | |
|---|---|---|---|
| H | 1.455773 | -4.219657 | 4.000430 |
| H | -0.925452 | -3.642560 | 3.635075 |
| C | 1.152426 | -3.727991 | 3.081939 |
| H | 3.115649 | -3.825529 | 2.202288 |
| C | -0.184588 | -3.388237 | 2.881556 |
| C | 2.089040 | -3.492630 | 2.074568 |
| C | -0.586574 | -2.754832 | 1.689565 |
| C | 1.705744 | -2.836133 | 0.891315 |
| H | -2.662054 | -3.284334 | 1.985239 |
| C | 0.374614 | -2.450455 | 0.718657 |
| H | 3.360317 | -3.478890 | -0.403049 |
| C | -2.007085 | -2.650573 | 1.375697 |
| C | 2.633567 | -2.675276 | -0.235259 |
| H | 0.066717 | -1.997607 | -0.215413 |
| N | -2.521394 | -2.001995 | 0.383943 |
| N | 2.574882 | -1.673248 | -1.037907 |
| H | 4.032213 | -2.609115 | -2.291772 |
| H | -4.494147 | -2.674987 | 0.893088 |
| C | 3.416690 | -1.702202 | -2.242425 |
| C | -3.895751 | -2.419970 | 0.010174 |
| H | 2.743767 | -1.707819 | -3.109210 |
| H | -3.801198 | -3.331956 | -0.594388 |
| C | -2.774889 | 2.283002 | 0.257731 |
| H | -3.717187 | 2.754420 | 0.561359 |
| C | 2.225781 | 2.411220 | 1.291774 |
| H | 2.932582 | 3.115720 | 1.741011 |
| O | -0.283214 | 0.684891 | 0.858203 |
| C | -0.298283 | 2.097401 | 0.868495 |
| C | -1.619053 | 2.715707 | 1.004019 |
| C | 0.834700 | 2.762528 | 1.506187 |
| H | -2.747273 | 4.331390 | 1.862848 |
| C | -1.764947 | 3.877332 | 1.767761 |
| O | -0.088263 | 0.500594 | -0.662742 |
| C | 0.615327 | 3.946906 | 2.213067 |
| H | 1.464523 | 4.465674 | 2.649305 |
| Cu | 1.919449 | 0.204498 | -0.731978 |
| Cu | -2.048403 | -0.342740 | -0.655251 |
| C | -0.666577 | 4.479242 | 2.382437 |
| H | -0.802490 | 5.381358 | 2.970250 |
| N | -2.752549 | 1.527388 | -0.807252 |
| N | 2.686649 | 1.460505 | 0.544826 |
| H | -4.876182 | -0.504291 | -0.161692 |
| H | 5.046551 | -0.541540 | -1.469228 |
| C | -4.595704 | -1.339594 | -0.809487 |
| C | 4.307619 | -0.461720 | -2.272564 |
| N | 3.501904 | 0.767819 | -2.039978 |
| N | -3.647949 | -0.837145 | -1.830483 |
| H | -5.516970 | -1.734275 | -1.256111 |
| H | 4.859368 | -0.407193 | -3.218118 |
| H | 3.241949 | 1.157404 | -2.944364 |
| H | -3.464199 | -1.581751 | -2.504282 |
| H | -4.888392 | 1.574820 | -0.925070 |
| H | 4.603465 | 0.599265 | 0.503687 |
| C | -4.011253 | 1.563097 | -1.586986 |
| C | 4.252580 | 1.806661 | -1.281793 |
| C | 4.125322 | 1.545091 | 0.226124 |
| C | -4.063590 | 0.385271 | -2.551859 |
| H | 3.807043 | 2.778971 | -1.513765 |
| H | -3.346690 | 0.534680 | -3.364721 |
| H | 5.309712 | 1.843609 | -1.575251 |
| H | -5.064172 | 0.291094 | -2.992618 |
| H | -4.031576 | 2.498918 | -2.156811 |
| H | 4.624940 | 2.348672 | 0.780647 |
| H | -0.096627 | 1.844324 | -0.479575 |

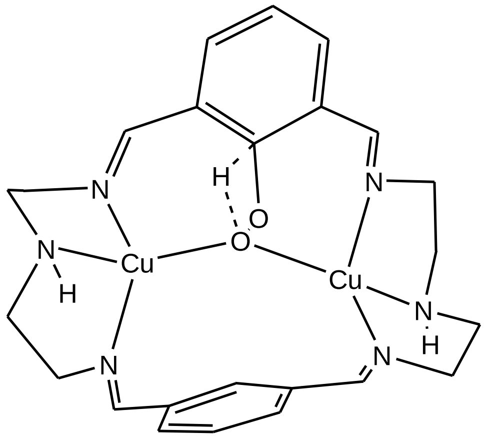


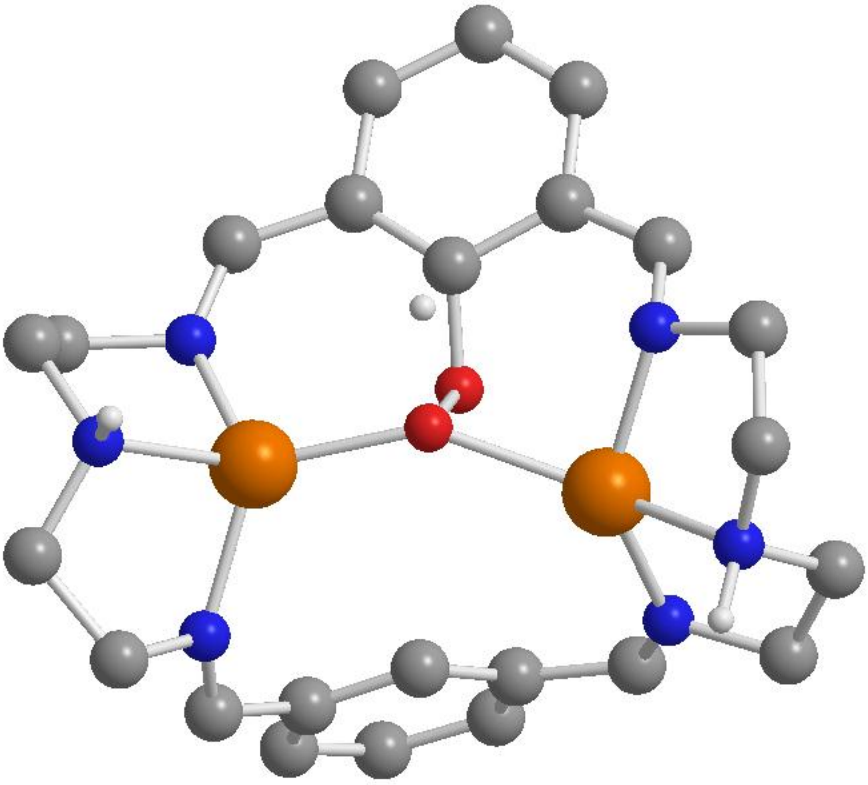

# Xh

| | | | |
|---|---|---|---|
| H | -5.293027 | -2.406894 | 1.365754 |
| H | -4.844796 | -1.690144 | -0.962735 |
| C | -4.273784 | -2.222470 | 1.042640 |
| H | -3.405039 | -2.855609 | 2.906983 |
| C | -4.020263 | -1.804613 | -0.263992 |
| C | -3.211468 | -2.462234 | 1.912406 |
| C | -2.701646 | -1.569040 | -0.694568 |
| C | -1.889004 | -2.204413 | 1.502117 |
| H | -3.221716 | -1.741210 | -2.794491 |
| C | -1.644704 | -1.740243 | 0.206976 |
| H | -0.943465 | -3.334212 | 3.114425 |
| C | -2.425032 | -1.402150 | -2.121696 |
| C | -0.770659 | -2.529132 | 2.390078 |
| H | -0.624210 | -1.588275 | -0.119519 |
| N | -1.297655 | -1.043553 | -2.623729 |
| N | 0.375219 | -1.945611 | 2.358957 |
| H | 1.137278 | -3.330843 | 3.801047 |
| H | -1.845560 | -0.929170 | -4.692011 |
| C | 1.413527 | -2.397815 | 3.295692 |
| C | -1.073115 | -1.369067 | -4.050269 |
| H | 2.329862 | -2.583196 | 2.722859 |
| H | -1.128293 | -2.458263 | -4.164605 |
| C | 1.360648 | 2.820347 | -2.572331 |
| H | 1.882860 | 3.546787 | -3.205494 |
| C | -0.818653 | 2.567405 | 1.955161 |
| H | -1.375583 | 3.203823 | 2.650767 |
| O | -0.902079 | 1.432138 | -1.167154 |
| C | -0.101432 | 2.349964 | -0.543121 |
| C | 0.466514 | 3.324145 | -1.564955 |
| C | -0.771010 | 3.067013 | 0.610863 |
| H | 0.389062 | 5.284481 | -2.422651 |
| C | 0.033646 | 4.635753 | -1.625242 |
| O | 1.157967 | 0.173789 | -0.007053 |
| C | -1.238258 | 4.364003 | 0.436407 |
| H | -1.820415 | 4.832099 | 1.227451 |
| Cu | 0.869199 | -0.020649 | 1.776984 |
| Cu | 0.263428 | 0.118257 | -1.929001 |
| C | -0.880156 | 5.141538 | -0.681355 |
| H | -1.248188 | 6.158007 | -0.771335 |
| N | 1.541468 | 1.552456 | -2.730909 |
| N | -0.189199 | 1.546889 | 2.458531 |
| H | 0.262952 | 0.187050 | -4.734345 |
| H | 0.740635 | -1.204834 | 4.955482 |
| C | 0.311234 | -0.872399 | -4.472732 |
| C | 1.628454 | -1.282976 | 4.321163 |
| N | 1.801514 | 0.018618 | 3.610873 |
| N | 1.249960 | -1.008818 | -3.325639 |
| H | 0.668028 | -1.418187 | -5.354398 |
| H | 2.485268 | -1.495015 | 4.969145 |
| H | 2.798870 | 0.163173 | 3.448880 |
| H | 1.337496 | -1.995450 | -3.077593 |
| H | 2.182263 | 1.342857 | -4.732892 |
| H | -0.806556 | 0.557300 | 4.214287 |
| C | 2.505044 | 1.066712 | -3.720829 |
| C | 1.263162 | 1.194625 | 4.359714 |
| C | -0.186790 | 1.416819 | 3.925257 |
| C | 2.606251 | -0.452531 | -3.567372 |
| H | 1.863622 | 2.069079 | 4.094126 |
| H | 3.207719 | -0.701191 | -2.688695 |
| H | 1.339829 | 1.044073 | 5.442140 |
| H | 3.074155 | -0.905036 | -4.449945 |
| H | 3.490885 | 1.519589 | -3.564576 |
| H | -0.601171 | 2.303855 | 4.417744 |
| H | 0.774222 | 1.651363 | -0.112480 |

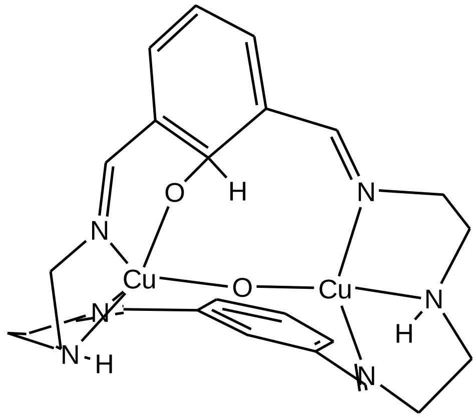


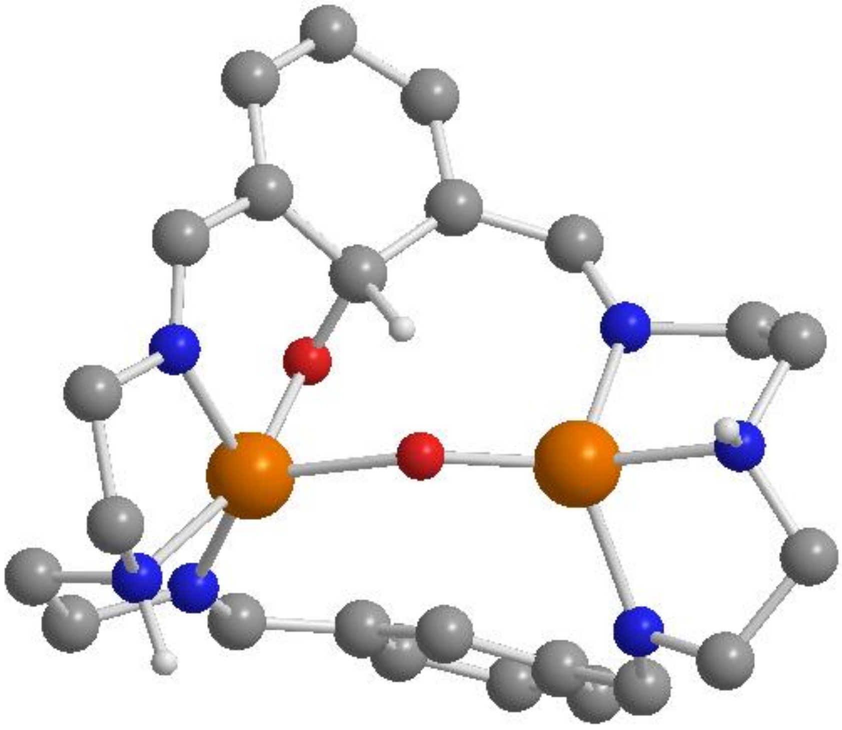

## References

[1] Becke, A. D. *J. Chem. Phys.* **1993**, *98*, 5648-5652.
[2] Lee, C.; Yang, W.; Parr, R. G. *Phys. Rev. B*, **1988**, *37*, 785-789.
[3] Stevens, P. J.; Devlin, F. J.; Chabalowski, C. F.; Frisch, M. J. *Phys. Chem.* **1994**, *98*, 11623-11627.
[4] (a) Hehre, W. J.; Ditchfield, R.; Pople, J. A. *J. Chem. Phys*. **1972**, *56*, 2257-2261. (b) Hehre, W. J.; Radom, L.; Schleyer, P. v. R.; Pople, J. A. Ab Initio Molecular Orbital Theory; Wiley: New York, 1986. Apart from 6-31-G(d) basis sets a single point energy calculation was done with the 6-31-G(d,p) and no qualitative change was observed apart from smaller barriers when the hydrogen atom is involved in the corresponding transition states.
[5] Frisch, M. J.; Trucks, G. W.; Schlegel, H. B.; Scuseria, G. E.; Robb, M. A.; Cheeseman, J. R.; J. A. Montgomery, J.; Vreven, T.; Kudin, K. N.; Burant, J. C.; Millam, J. M.; Iyengar, S. S.; Tomasi, J.; Barone, V.; Mennucci, B.; Cossi, M.; Scalmani, G.; Rega, N.; Petersson, G. A.; Nakatsuji, H.; Hada, M.; Ehara, M.; Toyota, K.; Fukuda, R.; Hasegawa, J.; Ishida, M.; Nakajima, T.; Honda, Y.; Kitao, O.; Nakai, H.; Klene, M.; Li, X.; Knox, J. E.; Hratchian, H. P.; Cross, J. B.; Adamo, C.; Jaramillo, J.; Gomperts, R.; Stratmann, R. E.; Yazyev, O.; Austin, A. J.; Cammi, R.; Pomelli, C.; Ochterski, J. W.; Ayala, P. Y.; Morokuma, K.; Voth, G. A.; Salvador, P.; Dannenberg, J. J.; Zakrzewski, V. G.; Dapprich, S.; Daniels, A. D.; Strain, M. C.; Farkas, Ö.; Malick, D. K.; Rabuck, A. D.; Raghavachari, K.; Foresman, J. B.; Ortiz, J. V.; Cui, Q.; Baboul, A. G.; Clifford, S.; Cioslowski, J.; Stefanov, B. B.; Liu, G.; Liashenko, A.; Piskorz, P.; Komaromi, I.; Martin, R. L.; Fox, D. J.; Keith, T.; Al-Laham, M. A.; Peng, C. Y.; Nanayakkara, A.; Challacombe, M.; Gill, P. M. W.; Johnson, B.; Chen, W.; Wong, M. W.; Gonzalez, C.; Pople, J. A.; 03 ed.; Gaussian, Inc: Pittsburgh PA, 2003.
[6] (a) Barone, V.; Cossi, M. *J. Phys. Chem. A*, **1998**, *102*, 1995-2001. (b) Tomasi, J.; Persico, M. *Chem. Rev*. **1994**, *94*, 2027-2094.
